# Supplementary material for: Cohort profile: a national, population-based cohort of children born after assisted conception in the UK (1992–2009): methodology and birthweight analysis
Source: BMJ Open. 2021 Jul 19;11(7):e050931. doi: 10.1136/bmjopen-2021-050931 (PMC8291329; doi:10.1136/bmjopen-2021-050931)
Supplement: Supplementary data [file bmjopen-2021-050931supp001.pdf]

Supplementary Files

S1: Completeness of variables (for the purpose of linkage and analysis) on the HFEA register

Variable	Useable* entry	Entry 'Null'	No entry	Other**	% useable out of total no. of entries
DOB	110,204	374	0	18	99.6%
Sex of Child<br>*Male*<br>*Female*	110,484<br>*56,265*<br>*54,219*	112	0	0	99.9%
BW	109, 157	534	0	905 records with BW <600g ***	98.7%
Child's Surname	12,332	97,671	0	593	11.2%
Child's Forename	11,402	97,670	0	1524	10.3%
Child's Town of Birth	70,738	39,689	61	108	64.0%
Child's District of Birth	18,833	91,452	298	13	17.0%
Child's Town ***or*** District of Birth	71,650	38,787	51	108	64.8%
Country of Birth	67554	38,861	0	4181 cases born outside of Great Britain	61.1%
Mother's Surname	110,596	0	0	0	100%
Mother's Forename	110,588	0	8	0	100.0%
Mother's Surname at Birth	51,237	58,714	209	436	46.3%
Mother's Forename at Birth	3,323	106,895	377	1	3.0%
Mother's DOB	110,569	1	0	26	100.0%
Father's Surname	110,450	138	8	0	99.9%
Father's Forename	110,384	202	8	2	99.8%
Father's DOB	110,282	257	0	57	99.7%
Mother's Town or District of Birth	62,505	47,045	0	1046	56.5%
Mother's Country of Birth	64,396	46,190	0	10	58.2%
Father's Town or District of Birth	61,143	48,354	0	1099	55.3%
Father's Country of Birth	63,100	47,485	0	11	57.1%
Treatment Cycle Start Date	110,596	0	0	0	100%
Treatment Centre No/ Name	110,594	0	0	2	100.0%

HFEA: Human Fertilization and Embryology Authority; DOB: date of birth; BW: birth weight

* 'Useable' data refers to records which contain data that appears useable in some analyses, including initial quality control analyses.

** 'Other' refers to unusable information for the variable in question, most commonly variations of 'Not known', 'Unknown' etc.

***905 records with documented BWs less than 600g were considered as invalid information for the purpose of deterministic linkage. Whilst a small number of these BWs (<600g) are likely to be correct, many more are likely to be data errors within the HFEA register. All actual recorded BWs were used when manually viewing potential matches.

This table was shared with us by HFEA at the start of the study.

S2- Flow diagram showing derivation of File 2 in Figure 1.

302, 487 Human Fertilisation & Embryology (HFEA) records relating to women treated with ART, 1991-2010, were available for linkage. These were transferred to NHS-digital & National Records of Scotland for linkage to NHS central registries of England & Wales and Scotland (EWS).

13,017 duplicates

- 11,290 - 2 or more distinct unique record numbers relating to the same individual
- 1,727 - duplicate unique record numbers.
- 1,722: Exact duplicate unique record numbers
- 5: Both had an exact duplicate unique record numbers and two distinct unique record numbers

22,683 'No trace' on linkage to NHSCR

- 8, 854 - HFEA recorded address outside England, Wales and Scotland (EWS) (no possibility of linkage to NHSCR)
- 13,829 - EWS based women, no trace on NHSCR

3 records - noted to have possible linkage error after data quality checks

266,784 of 280,616 eligible records successfully linked.

- ***<u>Linkage rate = 95.07% *</u>***

* Denominator excludes duplicate records and records of women based overseas who had treatment in the UK.

242 records - contained no clinical fertility data

122 records - embarked from EWS before treatment date, thus no outcome data available

266,420 of 280,616 records successfully linked and containing both fertility and outcome data.

- 94.94%

1,678 records **-** treatment date missing

52 records – DOB missing

8,904 - Women had a cancer diagnosis prior to the first calendar year of treatment.

255,786 of 280,616 potentially eligible records entered into the cohort analysis

- 91.15%

HFEA: Human Fertilization and Embryology Authority; DOB: date of birth; EWS: England, Wales & Scotland; NHSCR: National Health Service Central Register

S3: Order of pregnancy (HFEA-ONS cohort)

No. of pregnancies	Order of pregnancy	ART		Spontaneously conceived siblings	
		Singleton pregnancy	Twin Pregnancy	Singleton pregnancy	Twin Pregnancy
**2 (n=8441)**	1	5135	1182	2095	29
	2	1596	528	6161	156
**3 (n=1336)**	1	918	107	304	7
	2	453	96	777	10
	3	126	26	1155	29
**4 (n=125)**	1	83	9	33	0
	2	42	5	77	1
	3	19	6	99	1
	4	7	4	113	1
**5 (n=12)**	1	8	1	2	1
	2	5	1	6	0
	3	2	0	10	0
	4	0	0	12	0
	5	0	0	12	0
**6 (n=1)**	1	1	0	0	0
	2	0	0	1	0
	3	0	0	1	0
	4	0	0	1	0
	5	0	0	1	0
	6	0	0	1	0
**7 (n=1)**	1	0	1	0	0
	2	0	0	1	0
	3	0	0	1	1
	4	0	0	0	0
	5	0	0	1	0
	6	0	0	1	0
	7	0	0	1	0

HFEA: Human Fertilization and Embryology Authority; ONS: Office for National Statistics.

S4: Multiple regression coefficients for adjusted mean BW- ART vs NCP (HFEA-ONS)

Variables	BW Coefficient	95% Confidence interval
Embryo transfer status		
Fresh	-131.4	(-139.5 - -123.4)
Frozen	35.24	(18.97 - 51.52)
Sex		
Male	119.1	(112.7 - 125.5)
Maternal age group		
25-29	66.68	(55.89 - 77.48)
30-34	111.9	(102.2 - 121.6)
35-39	129.2	(118.7 - 139.7)
40-44	132.3	(118.0 - 146.5)
>=45	72.66	(-13.46 - 158.8)
**Constant**	3,215	(3,207 - 3,223)
**Observations**	129,304	
**R-squared**	0.021	

BW: birth weight; NCP: naturally conceived population controls; HFEA: Human Fertilization and Embryology Authority; ONS: Office for National Statistics.

S5: Multiple regression coefficients for adjusted mean BW- sART vs NCS (HFEA-ONS)

VARIABLES	BW coefficient	95% Confidence interval
Embryo transfer status		
Fresh	-54.69	(-72.64 - -36.73)
Frozen	138.6	(101.3 - 175.9)
Sex		
Male	115.6	(97.78 - 133.4)
Maternal age group		
25-29	-43.23	(-114.2 – 27.74)
30-34	-98.50	(-176.5 - -20.46)
35-39	-117.1	(-206.8 - -27.41)
40-44	-161.8	(-266.1 - -57.52)
>=45	-198.7	(-409.2 – 11.79)
Order of pregnancy		
2	165.6	(142.2 – 188.9)
>=3	237.0	(194.4 – 279.6)
**Constant**	3,337	(3,263 - 3,411)
**Observations**	22,086	
**R-squared**	0.081	

BW: birth weight; sART: ART with siblings; NCS- naturally conceived siblings; HFEA: Human Fertilization and Embryology Authority; ONS: Office for National Statistics.

S6: Multiple regression coefficients for adjusted mean BW- ART vs NCP (HFEA-ONS-HES cohort)

Variables	BW coefficient	95% confidence interval
Embryo transfer status		
Fresh	-152.2	(-162.2 - -142.3)
Frozen	2.855	(-16.61 - 22.32)
Sex		
Male	120.6	(112.7 - 128.6)
Maternal age group		
25-29	57.25	(43.52 - 70.99)
30-34	86.40	(73.80 - 99.00)
35-39	91.20	(77.62 - 104.8)
40-44	98.57	(80.37 - 116.8)
45-49	4.103	(-63.56 - 71.77)
Missing	94.69	(60.19 - 129.2)
IMD at earliest appointment		
2	25.95	(8.336 - 43.57)
3	56.66	(38.69 - 74.64)
4	58.78	(40.66 - 76.91)
5	75.13	(56.93 - 93.34)
6	93.33	(75.06 - 111.6)
7	93.21	(75.17 - 111.2)
8	111.8	(93.76 - 129.8)
9	117.3	(99.48 - 135.1)
10	134.7	(116.5 - 152.8)
Derived ethnicity		
Asian/Asian British	-247.8	(-264.2 - -231.4)
Black/African/Caribbean/Black British	-138.2	(-161.2 - -115.2)
Chinese	-120.1	(-195.7 - -44.43)
Mixed/multiple ethnic groups	-66.13	(-94.92 - -37.33)
Not stated/Not known	5.034	(-5.060 - 15.13)
Other ethnic group	-49.19	(-82.25 - -16.13)
**Constant**	3,177	(3,162 - 3,192)
**Observations**	85,366	
**R-squared**	0.040	

BW: birth weight; NCP- naturally conceived population controls; HFEA: Human Fertilization and Embryology Authority; ONS: Office for National Statistics; HES: Hospital Episode Statistics; IMD: Index of Multiple Deprivation

S7: Multiple regression coefficients for adjusted mean BW- sART vs NCS (HFEA-ONS-HES cohort)

Variables	BW coefficient	95% Confidence interval
Embryo transfer status		
Fresh	-57.01	(-75.79 - -38.23)
Frozen	152.1	(113.5 - 190.7)
Sex		
Male	115.6	(96.95 – 134.2)
Maternal age group		
25-29	-28.73	(-104.3 - -46.84)
30-34	-81.27	(-164.9 – 2.335)
35-39	-96.01	(-192.0 - -0.010)
40-44	-135.4	(-246.9 - -23.89)
>=45	-141.6	(-308.8 - -25.69)
Order of pregnancy		
2	152.2	(127.8 – 176.6)
>=3	229.6	(184.3 – 274.8)
**Constant**	3,332	(3,252 - 3,412)
**Observations**	19180	
**R-squared**	0.09	

BW: birth weight; sART: ART with siblings; NCS- naturally conceived siblings; HFEA: Human Fertilization and Embryology Authority; ONS: Office for National Statistics.

S8: Distribution of maternal age in records born before 1997 and not linked to HES

	ART	CONTROL	SIBLING
Maternal age group			
<=25	91 (0.79%)	5804 (15.57%)	84 (2.45%)
25-29	1360 (11.84%)	5496 (14.74%)	179 (5.22%)
30-34	4781 (41.62%)	7592 (20.37%)	636 (18.55%)
35-39	4318 (37.59%)	4738 (12.71%)	873 (25.46%)
40-44	921 (8.02%)	1160 (3.11%)	357 (10.41%)
>=45	11 (0.10%)	132 (0.35%)	27 (0.79%)
Missing	5 (0.04%)	12357 (33.15%)	1273 (37.12%)

HES: Hospital Episode Statistics
