# Supplementary material for: Cohort profile: a national, population-based cohort of children born after assisted conception in the UK (1992–2009): methodology and birthweight analysis
Source: BMJ Open. 2021 Jul 19;11(7):e050931. doi: 10.1136/bmjopen-2021-050931 (PMC8291329; doi:10.1136/bmjopen-2021-050931)
Supplement: Author's manuscript [file bmjopen-2021-050931.draft_revisions.pdf]

BMJ Open is committed to open peer review. As part of this commitment we make the peer review history of every article we publish publicly available.

When an article is published we post the peer reviewers' comments and the authors' responses online. We also post the versions of the paper that were used during peer review. These are the versions that the peer review comments apply to.

The versions of the paper that follow are the versions that were submitted during the peer review process. They are not the versions of record or the final published versions. They should not be cited or distributed as the published version of this manuscript.

BMJ Open is an open access journal and the full, final, typeset and author-corrected version of record of the manuscript is available on our site with no access controls, subscription charges or pay-per-view fees (http://bmjopen.bmj.com).

If you have any questions on BMJ Open's open peer review process please email
info.bmjopen@bmj.com

BMJ Open

Cohort Profile: A national, population based cohort of children born after assisted conception in the UK (1992-2009) - Methodology and birthweight analysis

Journal:	*BMJ Open*
Manuscript ID	bmjopen-2021-050931
Article Type:	Cohort profile
Date Submitted by the Author:	03-Mar-2021

Complete List of Authors:	Purkayastha, Mitana; UCL Great Ormond Street Institute of Child Health Population Policy and Practice<br>Roberts, Stephen; University of Manchester, Centre for Biostatistics,Institute of Population Health<br>Gardiner, Julian; University of Oxford, Education; UCL Great Ormond Street Institute of Child Health Population Policy and Practice,<br>Brison, Daniel; Central Manchester University Hospitals NHS Foundation Trust, Department of Reproductive Medicine<br>Nelson, Scott; University of Glasgow, School of Medicine<br>Lawlor, Deborah; University of Bristol Bristol Population Health Science Institute, MRC Integrative Epidemiology Unit<br>Luke, Barbara ; Michigan State University,<br>Sutcliffe, Alastair; UCL Great Ormond Street Institute of Child Health Population Policy and Practice

Keywords:	Subfertility < GYNAECOLOGY, EPIDEMIOLOGY, PAEDIATRICS

SCHOLARONE™
Manuscripts

BMJ

I, the Submitting Author has the right to grant and does grant on behalf of all authors of the Work (as defined in the below author licence), an exclusive licence and/or a non-exclusive licence for contributions from authors who are: i) UK Crown employees; ii) where BMJ has agreed a CC-BY licence shall apply, and/or iii) in accordance with the terms applicable for US Federal Government officers or employees acting as part of their official duties; on a worldwide, perpetual, irrevocable, royalty-free basis to BMJ Publishing Group Ltd ("BMJ") its licensees and where the relevant Journal is co-owned by BMJ to the co-owners of the Journal, to publish the Work in this journal and any other BMJ products and to exploit all rights, as set out in our licence.

The Submitting Author accepts and understands that any supply made under these terms is made by BMJ to the Submitting Author unless you are acting as an employee on behalf of your employer or a postgraduate student of an affiliated institution which is paying any applicable article publishing charge ("APC") for Open Access articles. Where the Submitting Author wishes to make the Work available on an Open Access basis (and intends to pay the relevant APC), the terms of reuse of such Open Access shall be governed by a Creative Commons licence – details of these licences and which Creative Commons licence will apply to this Work are set out in our licence referred to above.

Other than as permitted in any relevant BMJ Author's Self Archiving Policies, I confirm this Work has not been accepted for publication elsewhere, is not being considered for publication elsewhere and does not duplicate material already published. I confirm all authors consent to publication of this Work and authorise the granting of this licence.

For peer review only

Cohort Profile: A national, population based cohort of children born after assisted conception in the UK (1992-2009) - Methodology and birthweight analysis

Purkayastha M[a] [ORCID ID: https://orcid.org/0000-0002-1870-8838; email: m.purkayastha@ucl.ac.uk]

Roberts S.A[b] [ORCID ID: https://orcid.org/0000-0002-7477-7731; email: Steve.Roberts@manchester.ac.uk]

Gardiner J[c] [ORCID ID: https://orcid.org/0000-0001-8988-3812; email: julian.gardiner@education.ox.ac.uk]

Brison D[d] [ORCID ID: https://orcid.org/0000-0002-4307-1293; email: daniel.brison@manchester.ac.uk]

Nelson S.M[e] [ORCID ID: https://orcid.org/0000-0002-9099-3047; email: Scott.Nelson@glasgow.ac.uk]

Lawlor D.A[f] [ORCID ID: https://orcid.org/0000-0002-6793-2262; email: D.A.Lawlor@bristol.ac.uk]

Luke B[g] [ORCID ID: https://orcid.org/0000-0002-0405-8133; email: barbara.luke@hc.msu.edu]

Sutcliffe A.G[a] [ORCID ID: https://orcid.org/0000-0001-8542-6155; email: a.sutcliffe@ucl.ac.uk]

Author Affiliations:

[a] Great Ormond Street Institute of Child Health, University College London

[b] Division of Population Health, Health Services Research & Primary Care, University of Manchester

[c] Department of Education, University of Oxford

[d] Division of Developmental Biology & Medicine, University of Manchester

[e] School of Medicine, Dentistry & Nursing, Reproductive & Maternal Medicine, University of Glasgow

[f] Bristol Population Health Science Institute, University of Bristol

[g] Obstetrics, Gynaecology and Reproductive Biology,College Of Human Medicine, Michigan State University

Corresponding Author:

Mitana Purkayastha

30 Guilford Street

Population, Policy, and Practice

UCL Great Ormond Street Institute of Child Health

London WC1N 1DP

Email: m.purkayastha@ucl.ac.uk

Word count: 5548

For peer review only

ABSTRACT

Purpose: To generate a large cohort of children born after assisted reproductive technology (ART) in the UK between 1992 and 2009, their naturally conceived siblings (NCS), and matched naturally conceived population (NCP) controls and linking this with health outcome data to allow exploration of the effects of ART. The effects of fresh and frozen embryo transfer on birth weight were analysed to test the validity of the cohort.

Participants: Children recorded on the Human Fertilisation & Embryology Authority (HFEA) register as being born after ART between 1992 and 2009, their NCS, and matched NCP controls linked to Office for National Statistics birth registration dataset (HFEA-ONS cohort). This cohort was further linked to the UK Hospital Episode Statistics database to allow monitoring of the child's post-natal health outcomes up to 2015 (HFEA-ONS-HES sub-cohort).

Findings to date: The HFEA-ONS cohort consisted of 75348 children born after non-donor ART carried out in the UK between 1st April 1992 and 31st July 2009 and successfully linked to birth registration records, 14763 NCS and 164823 matched NCP controls. The HFEA-ONS-HES sub-cohort included 63877 ART, 11343 NCS, and 127544 matched NCP controls further linked to health outcome data. The exemplar analysis showed that children born after fresh embryo transfers were lighter (BW difference: -131g, 95%CI: -140, -123) and those born after frozen embryo transfers were heavier (BW difference: 35g, 95%CI: 19, 52) than the NCP controls. The within-sibling analyses were directionally consistent with the population control analyses, but attenuated markedly for the fresh vs natural conception (BW difference: -54g; 95% CI: -72, -36) and increased markedly for the frozen vs natural conception (BW difference: 152g; 95% CI: 113, 190) analyses.

Future plans: To utilise this cohort to explore the relationship between ART conception and short- and long-term health outcomes in offspring.

Keywords: assisted reproductive technology; birth weight; naturally conceived siblings; naturally conceived population controls; record linkage

STRENGTHS AND LIMITATIONS OF THIS STUDY

- Meticulous linkage of robust, routinely collected administrative health data to yield a large cohort that is nationally unique, thus increasing the generalisability, accuracy, and precision of results from subsequent analyses.
- Linkage to the hospital admissions and outpatient database provides long-term mortality and morbidity outcome data on offspring for use in longitudinal research, policy planning and strategic development.
- Identification of naturally conceived siblings as well as matched naturally conceived population controls allows exploration of the association of ART with adverse offspring outcomes while accounting for parental factors related to sub-fertility which may confound these associations.
- Comparison of findings between the two approaches mentioned above increases confidence in findings.
- The validity of the cohort was tested by means of an exemplar analysis.

For peer review only

INTRODUCTION

Assisted reproductive technology (ART) usage has increased annually since the first live birth in 1978, with over 8 million children born after ART globally (1). This increased utilization of ART has occurred concurrently with developments in our understanding of the impact of the early life environment on long term health (2-4) and most families with ART conceived children reported potential general health risks to their children as their paramount concern (5, 6), resulting in a concomitant increase in focus on the potential adverse short- and long-term effects on offspring. The inability to distinguish the relative importance of the effects of ART treatment factors and parental sub-fertility is a common limitation of many ART follow-up studies (7-9). This issue has been addressed in prospective cohorts by identifying control populations of children born naturally to parents with established subfertility [different from infertility in terms of the time of unwanted non-conception (10)]. One such well-known cohort provided the first evidence of differences in blood pressure and growth parameters in ART children (11, 12). Further evidence comes from within-ART comparisons showing that early life environment (e.g. embryo culture medium composition) is associated with changes in fetal growth, birth weight (BW) and child growth (13, 14), and studies of embryo cryopreservation have made a similar point more recently (15). These small studies have been essential in both directing and validating the associations identified from large population studies, including prospective cohort studies of ART conceptions only (e.g a Swedish study that followed-up 30,959 children born after ART) (16) or large, record linkage studies (e.g. one South Australian record linkage study compared 6163 ART and 302,811 naturally conceived (NC) offspring, while another study in Denmark compared 33,139 ART children to 555828 NC children from the population) (17, 18). The Committee of Nordic ART and Safety (CoNARTaS), created in 2008, utilised medical registry data from Denmark, Finland, Norway and Sweden to establish a large cohort of children conceived after ART or natural conception as well as women with at least one delivery after ART or natural conception, with the aim of examining long-term health outcomes in children born after ART (19-21).

While these large studies using conventional multivariable approaches to explore the association of ART with adverse offspring outcomes are essential in order to extrapolate effect sizes and risk estimates to the general ART population (22-24), they cannot fully account for parental factors related to sub-fertility which may confound these associations. This has been addressed in record linkage studies that used within sibling analyses (where comparisons are made between ART and their NC siblings) to better control for factors related to sub-fertility and other family confounders under the assumption that these parental factors would be the same (or very similar) within sibling

For peer review only - http://bmjopen.bmj.com/site/about/guidelines.xhtml

groups (23, 25). However, such analyses typically have lower statistical power due to restricted numbers, can be biased if there is individual level confounding, and the subset of ART children with siblings may not be representative of the ART population as a whole. Therefore, a comparison of the two approaches is valuable as similar results from both would increase confidence in findings (25-27).

In the United Kingdom, the proportion of children born after ART has increased from approximately 0.5% in 1992 to 2% in 2017 (28). Since August 1991 (29), it has been compulsory for every fertility clinic in the UK to report details of all treatments carried out to the Human Fertilisation & Embryology Authority (HFEA) (30, 31). Due to the mandatory nature of reporting, the completeness and quality of data related to some couple characteristics (e.g. age, duration of infertility) and ART procedures is high, enabling research analysing treatment outcomes including success rates and perinatal outcomes such as gestation, BW, and congenital anomalies (32-37). However, the quality of perinatal outcome data available on the HFEA register is questionable as it is patient reported via the ART clinics (38). Hann et al (2018) (39) showed that 1 in 15 (6%) BWs recorded on the HFEA register were incorrect compared to data recorded directly by the delivering maternity unit. Moreover, the HFEA register contains no information on the health outcomes of children beyond the immediate perinatal period.

A change in the law in 2009 (40) made it possible for researchers to use patient identifying data contained in the HFEA register from 1991-2009 for the purpose of linkage to offspring health outcome databases, thus allowing the largest ever population studies of ART childhood cancer (41) and early child growth (39). However, further studies have been limited by extensive data governance requirements and high associated costs, and prospective monitoring of outcomes in this population as they progress from childhood into adulthood has been prevented thus far by the paucity of identifiable information necessary for a variety of linkages.

Consequently, the primary objective of this study was to substantially enhance the research value of the HFEA register by utilising electronic record linkage methodology to establish a cohort of children consisting of those born after ART in the UK between 1992 and 2009, their naturally conceived siblings (NCS) and matched naturally conceived population (NCP) controls to allow exploration of outcomes present on the birth registration dataset and subsequent linkage to other datasets. The secondary objective was to create a sub-cohort of children born after ART in the UK, NCS and matched NCP controls and linking this with information on their postnatal health outcomes up to 2015. These databases will be made available to all researchers (subject to necessary approvals), allowing more precise risk estimates of associations of ART with many potential childhood (and maternal) outcomes. Finally, the third objective of this study was to validate the cohort and sub-

For peer review only

cohort produced by comparing the effects of fresh and frozen embryo transfer versus natural conception on singleton BW. Our previous linkage from the HFEA 1991-2009 cohort to maternity and child growth records confirmed evidence in the field that children born after frozen embryo transfers are heavier and those born after fresh embryo transfers are lighter than those that are naturally conceived (NC), and that these differences in BW are further associated with differences in child growth up to the age of 5 (39). However, this study was only able to examine the subset of ART children born in Scotland and the dataset created was not readily linkable to other datasets.

COHORT DESCRIPTION

Databases used

Human Fertilization & Embryology Authority Register (HFEA): The HFEA, an 'arm's length body' of the Department of Health, acts as an independent regulator of fertility treatment and research using human embryos in the UK (31). The HFEA Act 1990 (29) made prospective collection and storage of baseline information and birth outcomes on the HFEA register mandatory, although 'consent for disclosure of information for research' was not collected from patients who underwent treatment at a licensed fertility clinic prior to September 2009. The reliability and completeness of information relating to women who have undergone treatment on the HFEA register is considerably high (approximately 99.9%, 100% & 99.9% completeness for forename, surname, & DOB, respectively; Supplementary table S1), although it contains little identifiable information on children born after ART (e.g 11.2% and 10.3% completeness for child's surname and forename, respectively; Supplementary table S1).

Details of the data accessed from HFEA for this study are discussed below (see Linkage section).

Birth Registration Database*:* In the UK, all births are legally required to be registered by the Local Registration Service in partnership with the General Register Office (GRO) in England and Wales, making it the most complete data source available (42). The birth registration dataset is managed by ONS in England and Wales, while Scottish birth records are held by National Records for Scotland (NRS) who work closely with NHS Digital to ensure daily record transfers between the organisations using a secure and closed electronic system.

This study used the birth registration dataset to identify all children born to women known to have undergone ART as well as their NCS, and details of the data accessed have been discussed below (see Linkage section).

For peer review only

Hospital Episode Statistics (HES): Hospital Episode Statistics (HES) is a data warehouse containing details of all admissions, outpatient appointments and A & E attendances at NHS hospitals in England (43). Although the HES data warehouse has been operating since 1990 (44), linkage of the HES admitted patient care (APC) episodes longitudinally to the same individuals only commenced in 1997/98 when the patient's NHS number became a mandated return from hospitals (45). Furthermore, linkage of this APC dataset to other HES datasets such as outpatient, A & E, and adult critical care only commenced much later in 2003/2004, 2007/2008 and 2008/2009, respectively (45). This study linked the HES database to the ART children, NCS, and matched NCP control groups, and the data accessed for this purpose have been discussed below (see Linkage section). Although the current paper only uses demographic data (ethnicity, IMD) and BW from HES to carry out an exemplar analysis, further analyses of longitudinal health outcomes in these cohorts are already underway and will be published shortly.

Medical Integrated Database and Administration System (MIDAS): MIDAS is a system used by NHS Digital's Data Linkage and Extract Service (DLES) to replace the functionality previously provided by the Central Health Register Information System (CHRIS) application, specifically for the administration of information used within research and data linkage functions. It was developed to provide continuity for the existing list cleaning, flagging, tracing and cohort management services to maintain support for approved research and audit projects, National Cancer Registration and the ONS Longitudinal Study. It contains up to date demographic GP registration data via a daily feed from the Secondary User Service, standard demographic identifiers such as names and addresses, information on patients who have exited from and returned to the NHS, as well as cancer and death registration details. MIDAS can be considered as a window into the Personal Demographics Service database, providing bespoke data extracts in addition to regular patient tracking services. Extracts are provided on an individual as-and-when basis, and are subject to the regular DLES approvals process.

In this study, the cohort produced by Linkage 1 (discussed later) was traced on MIDAS for further demographic information (NHS number where necessary, postcode etc.) that would allow subsequent linkage to HES. Additionally, the matched NCP controls were also identified using MIDAS.

Inclusion and Exclusion criteria

ART: defined as "treatments or procedures that include in vitro handling of both human oocytes and sperm or embryos, for the purpose of reproduction" (46). This study included all children born after

For peer review only

non-donor ART conducted in the UK between 1st April 1992 and 31st July 2009, in keeping with legislative changes that influenced the ability to link HFEA data to other health datasets (39, 41). It was estimated that only 0.2% of patients receiving ART during the study period retrospectively withdrew consent (47).

NCS**:** All naturally conceived (NC) children born to women with at least one child born following ART were included. This comprised of both full (both parents the same) and maternal half-siblings (same mother different father), but identification of paternal half-siblings was not possible.

NCP controls**:** The birth registration dataset and MIDAS were used to identify two matched [by month and year of birth, sex, and multiplicity] NCP controls per ART child (ART: control = 1:2).

All children conceived in the UK after ART but born outside of England, Wales & Scotland, those born after ART to women who permanently lived outside the UK but travelled to the UK for ART treatment, and those born in Northern Ireland were excluded as it would not be possible to link them to ONS birth records. Additionally, siblings born outside of the study period (as their conception status could not be verified) as well as those born outside of England, Wales & Scotland were also excluded. Cases that had withdrawn consent for their data to be used for research and children born after donor ART were excluded in compliance with HFEA legislation which prevents the viewing of identifiable data relating to these children by any third party.

Linkage

As described above, the HFEA Register contains very few identifiers for children born after ART, thus limiting linkage to other nationally held datasets. Therefore, this study initially linked HFEA records of children born to women known to have undergone ART to nationally held birth records to add identifiers and NHS numbers which would enable further linkage with a variety of other datasets (shown in Figures 1 & 2).

Linkage 1: HFEA-ONS

Step 1 [1 & 2 from Figure 1]: The HFEA first identified children born after ART and assigned them pseudo-anonymised unique record numbers (URN). Maternal unique record numbers (mURN; created from a previous study (47) [Supplementary Table S2] were added to the file, which was encrypted and sent securely to NHS Digital.

For peer review only

This file contained the following variables: date of birth (DOB) of child, BW of child, sex of child, forename of mother, surname of mother, previous name(s) of mother, multiplicity (e.g., singleton, twin), and mother's DOB.

Step 2 [3 & 4 from figure 1]: NHS Digital created file 2 [from a previous study (47); Supplementary Table S2] containing extracts of women who had undergone ART within the specified time period and securely transferred it to ONS. This file contained the NHS Number, DOB of possible mother, surname of possible mother, forename of possible mother, other name(s) of possible mother, and the HFEA mURN.

ONS then used these variables to link to birth records to create an extract of all children born to these mothers within the specified time frame. This extract (File 3) contained the mothers' details as supplied in File 2, surname of the child, forename of the child, DOB of the child, NHS number of the child (if available), BW of the child, and sex of the child. This file was then securely transferred back to NHS Digital.

Step 3 [5, 6, 7, 8 & 9 from figure 1]: NHS Digital then matched files 1 & 3 to determine children born to these mothers after ART and their NCS. This linkage was carried out using DOB of child, NHS number of child (if available), BW of child, sex of child, surname of child (where available on HFEA file 1), and forename of child (where available on HFEA file 1).

The ART and sibling cohorts were then traced using NHS Digital's national Medical Integrated Database and Administration System (MIDAS) to update demographic information (including NHS number, postcode) and current status (emigrations/ deaths etc.) and allow subsequent linkage to medical records.

Step 4 [10 &11 from figure 1]: NHS Digital used MIDAS to identify 2 controls for every cohort member, matched for month and year of birth, sex, and multiplicity (singleton/ multiple birth).

Step 5 [14 from figure 1]: NHS Digital produced updated demographic information on all ART children, siblings and controls (as obtained from MIDAS) and provided de-identified outputs plus non-identifiable deprivation scores to the research team at University College London (UCL).

Linkage 2: HFEA-ONS-HES

Step 6 [15 on figure 2]: NHS Digital then linked all cohorts produced by linkage 1 to HES using NHS number, postcode, and DOB, and shared with the research team at UCL.

Step 7 [16 on figure 2]: HFEA staff extracted relevant anonymised fertility data and this was shared with the research team at UCL who linked these two files (from steps 6 and 7) using the URNs.

Step 8 [17 on figure 2]: For the purpose of this second linkage, UCL researchers excluded records of children born before 1st April 1997 to coincide with the start of HES monitoring as well as triplets

For peer review only

and higher order births from all groups (along with associated ART and NC births) to produce the final cohorts for health outcome analysis.

Step 9 [18 on figure 2]: NHS Digital produced file 4 (shown in Figure 1) for the ART and NC cohorts and these are now securely stored at HFEA and NHS Digital, respectively.

The entire linkage process was completed by NHS-Digital and none of the researchers had access to any identifiable participant data. The details of the ART and NC groups were encrypted and are currently held securely at HFEA and NHS-Digital, respectively.

Cohorts produced

Linkage 1: HFEA-ONS cohort

a) *ART***:** This group consisted of all children born after non-donor ART conducted in the UK between 1st April 1992 and 31st July 2009.

b) *NCS:* This included all NC children born to women who had at least one child born following ART. This included full and maternal half-siblings but not paternal half-siblings.

c) *NCP:* This consisted of two matched (by month and year of birth, sex, and multiplicity) NCP controls for each ART child.

Linkage 2: HFEA-ONS-HES sub-cohort

a) *ART with HES outcomes (HES-ART):* This included all children born after non-donor ART conducted in the UK between 1st April 1997 and 31st July 2009 linked to their HES outcomes.

b) *NCS with HES outcomes (HES-NCS):* This included the NCS of all non-donor ART children born in the UK between 1st April 1997 and 31st July 2009 linked to their HES outcomes.

c) *NCP control with HES outcomes (HES-NCP):* This included two matched NCP controls for each non-donor ART child born in the UK between 1st April 1997 & 31st July 2009 linked to their HES outcomes.

Data Cleaning

Data cleaning included deletion of triplets and higher order births along with their NCS and matched NCP controls; deletion of duplicates; and reformatting, labelling and creation of new variables. Triplets and higher order births were excluded from analysis as they are known to be associated with adverse perinatal outcomes such as higher infant mortality, birth defects, premature birth, and low BW (48, 49). Initial data scoping also revealed several discrepancies in the data, a common limitation of large data linkage studies utilising multiple data sources, and pragmatic rules were employed to

For peer review only

derive a consensus. Additional data cleaning carried out on the sub-cohort produced via linkage 2 included creation of an additional group consisting of ART children who had NCS (HES-sART) for the purposes of analysis. Order of pregnancy was calculated by sorting the child DOBs within each family in ascending order (Supplementary tables S3), and the type of ART and cause of infertility variables were regrouped appropriately.

Exemplar analysis

An exemplar analysis examining the effect of fresh and frozen embryo transfer (each compared to NC) on singleton BW was carried out using the HFEA-ONS cohort and HFEA-ONS-HES sub-cohort. Details of BW are provided by the hospital where the birth took place or by the midwife/doctor in attendance at the birth, and is then passed to ONS as a consequence of the NHS birth notification being linked to the corresponding birth registration by the registrar. Multiple regression analysis was used to compare BW between children born after ART and NCP controls. The models were adjusted for maternal age at delivery and sex for the ART vs NCP analysis, and for maternal age at delivery, sex, IMD at earliest appointment, and ethnicity for the HES-ART vs HES-NCP analysis.

A family-matched model was used to compare BW between ART children and their NCS, including a family covariate to allow for within family correlations. The models were adjusted for maternal age at delivery, sex, and order of pregnancy. IMD and ethnicity were excluded from this analysis as the underlying effects they represent would have remained constant within families. The models were parameterised to directly estimate the differences between ART (fresh and frozen) and NC children. The fresh vs frozen difference was estimated from the model by construction of the appropriate contrast. All analyses were carried out using Stata v16.0.

This paper was developed in accordance with the STROBE reporting guidelines (50).

Patient and Public Involvement

Unfortunately, due to the very personal nature of the treatments involved, it was not appropriate to contact the families directly thus preventing us from involving patients or the public in the design, conduct, reporting, or dissemination plans of our research.

For peer review only

FINDINGS TO DATE

Final HFEA-ONS cohort

The HFEA identified 110596 children that met our inclusion criteria, of which 97660 (Figure 1) were deemed suitable for transfer to NHS Digital and subsequent linkage to ONS birth records. The linkage success rate was 77% (75348 cases out of 97660) due to the poor quality of data received from the HFEA.

Tables 1 & 2 shows the descriptive characteristics of the final HFEA-ONS cohort generated after completion of Linkage 1 (Figure 1) and data cleaning. This cohort included 75348 children born after ART between 1992 and 2009 and 164823 matched NCP controls. Of the children born after ART, 15875 (sART) had 14763 NCS and the majority of families had had an ART pregnancy first followed by natural conception (Supplementary table S3).

The majority of the final cohort were ethnically white and singleton births, and a greater proportion of the ART and NCS groups were from a higher socioeconomic class. The majority of women who underwent ART were aged between 30-39 years at the time of delivery, while those with NC births tended to be slightly younger (25-39 years).

For peer review only

Table 1:Characteristics of children born between 1st April 1992 & 31st July 2009

	HFES-ONS cohort			
	ART*	Control (NCP)	ART with siblings (sART)	Siblings (NCS)
**Infants**	75348	164823	15875	14763
**Mean BW (S.D)**				
Singleton	3282.00 (620.27)	3353.83 (578.47)	3311.12 (605.10)	3430.05 (581.82)
Multiple	2378.25 (593.44)	2340.28 (626.06)	2405.10 (608.05)	2292.03 (780.45)
**Sex**				
Female	36994 (49.09%)	80859 (49.06%)	7598 (47.86%)	7271 (49.25%)
Male	38354 (50.91%)	83964 (50.94%)	8277 (52.14%)	7492 (50.75%)
**Multiplicity****				
Singleton children	44488 (59.06%)	99554 (60.40%)	10592 (66.71%)	14055 (95.20%)
Multiple children	30860 (40.94%)	65269 (39.60%)	5283 (33.29%)	708 (4.80%)
**Maternal age group**				
<25	731 (0.97%)	33156 (20.12%)	256 (1.61%)	388 (2.63%)
25-29	5315 (7.05%)	30231 (18.34%)	1326 (8.35%)	1032 (6.99%)
30-34	23342 (30.98%)	46050 (27.94%)	5466 (34.44%)	3855 (26.11%)
35-39	29187 (38.74%)	30345 (18.41%)	5872 (36.98%)	5711 (38.69%)
40-44	8705 (11.55%)	7812 (4.74%)	1315 (8.28%)	2157 (14.61%)
>=45	84 (0.11%)	587 (0.36%)	23 (0.14%)	53 (0.36%)
Missing	7984 (10.60%)	16642 (10.10%)	1617 (10.18%)	1567 (10.61%)
**Birth year**				
1992	57 (0.08%)	131 (0.09%)	12 (0.08%)	23 (0.16%)
1993	1595 (2.12%)	3187 (1.93%)	299 (1.8%)	337 (2.28%)
1994	2021 (2.68%)	4029 (2.44%)	425 (2.68%)	389 (2.63%)
1995	2439 (3.24%)	4911 (2.98%)	518 (3.26%)	526 (3.56%)
1996	2977 (3.95%)	5989 (3.63%)	641 (4.04%)	577 (3.91%)
1997	3574 (4.74%)	7172 (4.35%)	776 (4.89%)	679 (4.60%)
1998	3826 (5.08%)	7670 (4.65%)	861 (5.42%)	800 (5.42%)
1999	4253 (5.64%)	8531 (5.18%)	1023 (6.44%)	804 (5.45%)
2000	4504 (5.98%)	9106 (5.52%)	1119 (7.06%)	961 (6.51%)
2001	4754 (6.31%)	9677 (5.87%)	1163 (7.33%)	991 (6.72%)
2002	5149 (6.83%)	10464 (6.35%)	1339 (8.43%)	999 (6.77%)
2003	5531 (7.34%)	11373 (6.90%)	1401 (8.82%)	1064 (7.21%)
2004	5671 (7.53%)	11835 (7.18%)	1379 (8.69%)	1098 (7.44%)
2005	5768 (7.66%)	12310 (7.47%)	1372 (8.64%)	1109 (7.51%)
2006	6331 (8.40%)	14248 (8.64%)	1273 (8.02%)	1116 (7.56%)
2007	6410 (8.51%)	15976 (9.69%)	1126 (7.09%)	1209 (8.19%)
2008	6395 (8.49%)	17222 (10.45%)	719 (4.53%)	1271 (8.61%)
2009	4092 (5.43%)	10992 (6.67%)	429 (2.70%)	810 (5.49%)

For peer review only

Table 2: Fertility characteristics

	HFEA-ONS cohort		HFEA-ONS-HES sub-cohort	
	ART	sART (ART with	HES-ART	HES-sART (ART
**Multiplicity**				
Singletons	44489 (59.04%)	10592 (66.71%)	37891 (59.37%)	8383 (67.99%)
Twins	30859 (40.96%)	5283 (33.29%)	25986 (40.63%)	3946 (32.02%)
**Paternal age at childbirth**				
<25	406 (0.54%)	122 (0.77%)	324 (0.51%)	87 (0.71%)
25-29	4878 (6.48%)	1206 (7.61%)	3911 (6.13%)	938 (7.60%)
30-34	21526 (28.57%)	5304 (33.42%)	17706 (27.71%)	4116 (33.39%)
35-39	27678 (36.73%)	6007 (37.82%)	23626 (36.99%)	4692 (38.05%)
40-44	13458 (17.86%)	2306 (14.54%)	11715 (18.34%)	1778 (14.41%)
45-49	4551 (6.03%)	604 (3.80%)	4010 (6.28%)	468 (3.79%)
50-54	1636 (2.18%)	190 (1.21%)	1440 (2.27%)	146 (1.19%)
>=55	848 (1.12%)	82 (0.51%)	787 (1.23%)	65 (0.53%)
Missing	367 (0.49%)	54 (0.35%)	348 (0.55%)	39 (0.32%)
**Type of ART**				
ICSI	26629 (38.61%)	4409 (30.09%)	26210 (44.62%)	3995 (34.95%)
IVF	42096 (61.03%)	10170 (69.41%)	32282 (54.96%)	7364 (64.42%
IVF:ICSI	248 (0.36%)	73 (0.50%)	247 (0.42%)	72 (0.63%)
**Cause of infertility**				
Male factor	29203 (38.78%)	5675 (35.77%)	24876 (38.96%)	4198 (34.07%)
Endometriosis	2225 (2.95%)	495 (3.12%)	1962 (3.07%)	401 (3.25%)
Ovulatory	8581 (11.39%)	2198 (13.85%)	7713 (12.08%)	1854 (15.05%)
Tubal	19915 (26.44%)	3450 (21.74%)	15041 (23.56%)	2378 (19.30%)
Unknown cause	15387 (20.43%)	4048 (25.51%)	14252 (22.32%)	3491 (28.33%)
**Fresh/Frozen transfer**				
Fresh	66534 (88.30%)	14079 (88.69%)	56230 (88.03%)	10907 (88.46%)
Frozen	8739 (11.60%)	1782 (11.22%)	7595 (11.89%)	1414 (11.47%)
Missing	75 (0.10%)	14 (0.09%)	52 (0.08%)	8 (0.06%)
**Previous live births**				
0	67235 (89.23%)	14878 (93.71%)	56250 (88.06%)	11498 (93.25%)
1	7829 (10.40%)	958 (6.05%)	7348 (11.50%)	795 (6.45%)
2	273 (0.36%)	35 (0.22%)	268 (0.42%)	32 (0.26%)
3	9 (0.01%)	2 (0.01%)	9 (0.1%)	2 (0.02%)
Missing	2 (0.00%)	2 (0.01%)	2 (0.00%)	2 (0.02%)
**Parity of mother (previous pregnancies as recorded on the register)**				
0	64502 (85.60%)	14270 (89.88%)	53987 (84.52%)	11029 (89.45%)
1	9798 (13.01%)	1450 (9.15%)	8901 (13.93%)	1167 (9.47%)
2	949 (1.26%)	140 (0.89%)	894 (1.40%)	119 (0.97%)
3	83 (0.11%)	9 (0.06%)	79 (0.12%)	8 (0.06%)
4	14 (0.02%)	4 (0.03%)	14 (0.02%)	4 (0.03%)
Null	2 (0.00%)	2 (0.01%)	2 (0.00%)	2 (0.02%)
**Infertility duration in years**				
Mean (SD)	4.78 (2.92)	4.37 (2.61)	4.62 (2.84)	4.31 (2.56)
Missing	17451 (23.16%)	4951 (31.19%)	17898 (24.90%)	5364 (27.14%)

For peer review only

Table 3:Characteristics of children born between 1st April 1997 and 31st July 2009

	HFEA-ONS-HES sub-cohort			
	**HES-ART**	**Control (HES-NCP)**	**ART with siblings (HES-sART)**	**Siblings (HES-NCS)**
**Infants**	63877	127544	12329	11343
**Follow-up period in days**				
**Median (IQR)**	4429 (2181)	4409 (2141)	4635 (1846)	4307 (2029)
**Mean BW (S.D)**				
Singleton	3166.95 (742.25)	3271.67 (648.46)	3222.25 (699.31)	3346.54 (712.02)
Multiple	2172.27 (715.42)	2155.77 (683.93)	2201.58 (724.87)	2301.17 (677.82)
**Sex**				
Female	31435 (49.21%)	62785 (49.22%)	5907 (47.91%)	5573 (49.12%)
Male	32442 (50.79%)	64759 (50.78%)	6422 (52.09%)	5770 (50.88%)
**Multiplicity****				
Singleton children	37890 (59.36%)	75642 (59.30%)	8383 (68.04%)	10815 (95.83%)
Multiple children	25987 (40.64%)	51902 (40.70%)	3946 (31.96%)	528 (4.17%)
**IMD decile at earliest**				
1 (most deprived)	2045 (3.20%)	12696 (9.94%)	349 (2.82%)	299 (2.63%)
2	2650 (4.15%)	11104 (8.71%)	458 (3.72%)	396 (3.51%)
3	3311 (5.19%)	10057 (7.87%)	539 (4.40%)	463 (4.07%)
4	3926 (6.14%)	9550 (7.49%)	678 (5.44%)	588 (5.19%)
5	4666 (7.28%)	9161 (7.49%)	862 (6.98%)	746 (6.57%)
6	5350 (8.39%)	8872 (6.95%)	1084 (8.82%)	921 (8.09%)
7	6142 (9.61%)	8842 (6.94%)	1205 (9.77%)	1044 (9.20%)
8	6675 (10.46%)	8925 (7.01%)	1299 (10.54%)	1161 (10.25%)
9	7729 (12.10%)	9083 (7.11%)	1557 (12.58%)	1386 (12.21%)
10 (least deprived)	7710 (12.07%)	8299 (6.53%)	1720 (14.00%)	1499 (13.25%)
Missing	13673 (21.41%)	30955 (24.26%)	2580 (20.94%)	2840 (25.02%)
**Birth year**				
1997	2597 (4.06%)	5160 (4.07%)	518 (4.21%)	242 (2.14%)
1998	3708 (5.81%)	7389 (5.85%)	743 (6.03%)	419 (3.68%)
1999	4083 (6.40%)	8126 (6.49%)	852 (6.92%)	560 (4.94%)
2000	4310 (6.75%)	8633 (6.91%)	925 (7.51%)	781 (6.85%)
2001	4559 (7.14%)	9160 (7.33%)	972 (7.89%)	879 (7.76%)
2002	4980 (7.79%)	9933 (7.87%)	1171 (9.50%)	937 (8.27%)
2003	5379 (8.42%)	10788 (8.49%)	1253 (10.17%)	1012 (8.92%)
2004	5561 (8.71%)	11082 (8.66%)	1271 (10.31%)	1067 (9.41%)
2005	5662 (8.87%)	11326 (8.83%)	1271 (10.30%)	1078 (9.51%)

Final HFEA-ONS-HES sub-cohort

All births before 1st April 1997 were excluded for the purpose of linkage to HES, resulting in 63877, 11343 and 127544 children in the HES-ART, HES-NCS, and HES-NCP groups, respectively. Linkage 2 (Figure 2) had a success rate of 84.7%, and the descriptive characteristics of the sub-cohort produced have been shown in Tables 2 & 3

For peer review only

2006	6275 (9.82%)	12513 (9.68%)	1217 (9.86%)	1100 (9.70%)
2007	6342 (9.93%)	12701 (9.84%)	1058 (8.56%)	1199 (10.56%)
2008	6347 (9.93%)	12718 (9.81%)	670 (5.44%)	1260 (11.11%)
2009	4074 (6.37%)	8015 (6.17%)	408 (3.30%)	809 (7.14%)
**Ethnicity**				
White	61921 (96.94%)	122050 (95.69%)	11983 (97.19%)	11084 (97.72%)
Asian/Asian British	959 (1.50%)	2496 (1.96%)	197 (1.60%)	153 (1.35%)
Chinese	35 (0.05%)	89 (0.07%)	8 (0.06%)	1 (0.01%)
Black/African/Caribbean/Black	433 (0.68%)	5268 (4.04%)	61 (0.49%)	39 (0.34%)
Mixed/Multiple ethnic groups	318 (0.50%)	721 (0.57%)	38 (0.31%)	27 (0.24%)
Other ethnic group	211 (0.33%)	498 (0.39%)	42 (0.34%)	39 (0.34%)
**Maternal age at delivery**				
<=25	710 (1.11%)	27783 (21.88%)	241 (1.95%)	317 (2.86%)
25-29	5085 (7.96%)	25115 (19.70%)	1209 (9.81%)	887 (7.80%)
30-34	21994 (34.44%)	38896 (30.50%)	4797 (38.92%)	3309 (29.12%)
35-39	27682 (43.35%)	25907 (20.29%)	4998 (40.57%)	4954 (43.67%)
40-44	8164 (12.99%)	6419 (5.21%)	1057 (8.57%)	1830 (16.15%)
>=45	217 (0.34%)	772 (0.61%)	20 (0.16%)	45 (0.40%)
Missing	25 (0.04%)	2652 (2.02%)	7 (0.01%)	1 (0.00%)

Exemplar analysis: Singleton BW

The exemplar analysis included all singleton births in the HFEA-ONS cohort and HFEA-ONS-HES sub-cohort.

HFEA-ONS: Comparing ART to NCP showed that children born after fresh embryo transfers were lighter (BW difference: -131g; 95% CI: -140, -123) and those born after frozen embryo transfers were heavier (BW difference: 35g; 95% CI: 19, 52) than the NC controls (Table 4; Supplementary table S4). Moreover, children born after frozen embryo transfers were significantly heavier than those born after fresh embryo transfers (BW difference: -167g; 95% CI: -183, -150).
Family-matched analysis showed that children born after fresh embryo transfers were lighter (BW difference: -54g; 95% CI: -72, -36) and those born after frozen embryo transfers (BW difference: 138g; 95% CI: 101, 175) were heavier than the NCS group (Table 4; Supplementary table S5). Moreover, children born after frozen embryo transfers were significantly heavier than those born after fresh embryo transfers (BW difference =-193g; 95% CI: -232, -154).

HFEA-ONS-HES: Comparing HES-ART to HES-NCP showed that children born after fresh embryo transfers were lighter (BW difference: -152g; 95% CI: -162, -142) and those born after frozen embryo transfers were similar in weight (BW difference: 3g; 95% CI: -17, 22) to the NC controls (Table 4;

For peer review only

Supplementary table S6). Moreover, children born after frozen embryo transfers were significantly heavier than those born after fresh embryo transfers (BW difference:-155g; 95% CI: -175, -135). Family-matched analysis comparing showed that children born after fresh embryo transfers were lighter (BW difference: -57g; 95% CI: -75, -38) and those born after frozen embryo transfers were heavier (BW difference: 152g; 95% CI: 113, 190) than the HES-NCS group (Table 4; Supplementary table S6). Moreover, children born after frozen embryo transfers were significantly heavier than those born after fresh embryo transfers (BW difference: -209g; 95% CI: -249, -168).

Table 4: Statistical analysis of BW

	**HFEA-ONS cohort**		**HFEA-ONS-HES sub-cohort**	
	**ART (N=75348) – NCP (N=164823)** *BW coefficient (95%CI)*	**sART (N=15875) NCS (N=14763)** *BW coefficient (95%CI)*	**HES-ART (N=63877) HES-NCP (N=127544)** *BW coefficient (95%CI)*	**HES-sART (N=12329) – HES-NCS (N=11343)** *BW coefficient (95%CI)*
Fresh vs NC (g)	-131 (-140, -123)	-54 (-72, -36)	-152 (-162, -142)	-57 (-75, -38)
Frozen vs NC (g)	35 (19, 52)	138 (101, 175)	3 (-17, 22)	152 (113, 190)
Fresh vs Frozen (g)	-167 (-183, -150)	-193 (-232, -154)	-155 (-175, -135)	-209 (-249, -168)

*NC: naturally conceived

DISCUSSION

This study aimed to substantially enhance the research value of the HFEA register by utilising electronic record linkage methodology to establish a cohort of ART children born in the UK between 1992 and 2009, their NCS and matched NCP controls. Additionally, a sub-cohort consisting of those born between 1997 and 2009 was also linked to the Hospital Episode Statistics database to allow examination of postnatal health outcomes. The final cohort consisted of 75348 children born after non-donor ART carried out in the UK between 1st April 1992 and 31st July 2009, 14763 NCS and 164823 matched NCP controls. Of these, 63877 ART, 11343 NCS, and 127544 matched NCP controls were linked to hospital data up to 2015, thus providing a valuable resource for comprehensive, non-

For peer review only

invasive, continued immediate and longer term health monitoring of this population as they grow up.

An exemplar analysis comparing BW between children born after ART, their NCS and matched NCP controls was also carried out to demonstrate the validity of this cohort. The results of this analysis confirmed the findings of our previous linkage study on a subset of the entire 1992-2009 cohort (39), that children born after fresh embryo transfer tended to be lighter and those born from frozen transfer were heavier compared to their NCS and matched NCP controls. These findings are broadly in agreement with numerous large register-based studies and meta-analyses, but add valuable confirmation of the magnitude of BW differences in a large UK population (15, 51-59). Castillo et al (60) reported a similar magnitude of difference to that seen in sibling pairs in a more recent UK cohort, suggesting that the difference in BW between the fresh and frozen embryo transfer groups may not be explained entirely by maternal characteristics and the underlying cause of infertility as these were likely to have remained relatively stable between siblings. Notably, the BW difference observed between frozen transfer and NC babies is much larger in the sibling pair analysis than in the non-sibling comparison, and larger than observed by Hann et al (39). This merits further investigation, especially as increased rates of macrosomia following frozen transfer are an ongoing concern (34, 56). Other possible explanations include differences in the maternal uterine environment caused by either hormonal stimulation (fresh cycles) or hormonal preparation of the uterus (frozen cycles) (61), although a recent UK cohort study reported no association with either parameter (60). Molecular changes in the early embryo during freezing and thawing might also result in changes in the developmental process leading to altered BW and long term health (33, 62-65).

In the current study, the difference in BW between children born after fresh embryo transfer and those born after natural conception was seen to attenuate markedly from -131g in the population to -54g in the within-sibling analysis. By contrast, the difference in BW between children born after frozen embryo transfer and those born after natural conception was seen to markedly increase from 35g in the population to 138g in the within sibling analysis in the same dataset. This suggests that the findings of the within-family analysis must be interpreted with caution, with potential explanatory factors for these differences including subfertility and familial masking confounders in relation to the increase of the association of frozen transfer vs natural conception. Unavailability of birth order data for the population controls prevented us from adjusting for it in the ART vs NCP comparison, and this could plausibly act as a strong confounder given its association with mean BW is stronger than any differences due to ART in most studies. This notable difference between the population and within-family analyses requires further exploration.

For peer review only

Repeating the exemplar analysis using the cohort and sub-cohort produced by the linkages described above not only increased confidence in the findings but also allowed us to control for potential confounding factors such as maternal age, birth order, socioeconomic status and ethnicity which are known to affect BW (66).

The cohort described here will allow more effective use of HFEA data for health monitoring, and will be of value to researchers from a variety of professional backgrounds. The linked dataset will be returned to the HFEA and access will be controlled by them, although specific ethical approval will be required along with that of the Confidentiality Advisory Group (CAG) of the Health Research Authority (HRA).

In order to provide reliable risk estimates of relatively rare conditions in a specific group of children born after ART, large, well-designed cohort studies, preferably including families, are necessary. However, as it may not always be appropriate to contact these families directly due to the very personal nature of the treatments involved, access to national-level electronically linked data on cohorts such as the current one provides a cost-effective, non-intrusive, and comprehensive way to answer sophisticated research questions in a wide range of areas.

The main strength of this study lies in the meticulous linkage of robust, routinely collected administrative health data to yield a large cohort that is nationally unique and complements other similar linked cohorts, such as the Nordic linked cohorts (19), thus increasing the generalisability, accuracy, and precision of results from subsequent analyses. The research value and quality and consistency of the HFEA cohort 1992-2009 data has been considerably enhanced through linkage to the ONS birth records and HES databases and removal of artefacts and duplicates. Linkage to the hospital admissions and outpatient database provides long-term mortality and morbidity outcome data on offspring, and also represents a high-quality cross-sectoral evidence base that can be used for longitudinal research, policy planning and strategic development.

However, there are also several challenges associated with electronic data linkage studies in general, and Harron et al. (67) summarised these into three groups, namely those pertaining to the (i) the data linkage environment and privacy preservation; (ii) the linkage process itself, which includes data preparation linkage methods; and (iii) the linkage quality and potential bias in linked data. In the context of the current study, limitations in the linkage process itself included those associated with the method of definition of NC siblings used. The identification of sibling controls would be very sensitive to any errors in linkage, and missed second ART babies would appear as conventional siblings. Parents who had NC children were likely to have been less severely sub-fertile than parents who did not, and ART children born to the truly infertile would not have conventional siblings. Therefore, the NCS group in this study would have parents with borderline fertility problems or

For peer review only

those who developed secondary infertility after the birth of the NC child. For this reason, extensive quality assurance procedures were carried out on the linkage process. With regard to the linkage quality, as this was a bespoke linkage that has never been carried out before, there were no standardized quality assurance measures in place; however, the linkage was extensively checked manually by the team at NHS Digital prior to dissemination for analysis.

As HES monitoring data is only available from 1997, children born to women who underwent ART prior to this could not be linked to any hospital records, thus limiting our ability to examine health outcomes in those born before 1997 and exploring the effects of changes in ART techniques on health over that period. Nevertheless, linkage of cases born before 1997 to their birth records is still valuable as it may allow investigation of outcomes available on the birth register in the future. Moreover, although these data have been used extensively for research purposes, there have been long-standing concerns regarding the quality, completeness and coverage of HES records within health services and the academic community (68).

The BW analysis provides an example use of these cohorts and the strengths and limitations of sibling comparisons. However, it cannot be considered as an exhaustive analysis as gestational data was not available for NC children and there is limited covariate data in the present dataset. This is mainly because gestation is not recorded on the birth registration dataset and is of poor quality on the maternal arm of a child's HES record (>50% missing). As a result, although the quality of such data for the ART group (accessed from the HFEA register) was good, it was not available for the NCS and NCP control groups. Further ART treatment data is available on the HFEA register which was not included in the current datasets, and these could be extracted for future studies. In particular the developmental stage of the embryo at transfer and number of embryos transferred are important determinants of treatment success rate (i.e. the probability of a child appearing on the HFEA birth register) and also gestation and BW (37, 39, 60). Unfortunately, there was no way to systematically identify and account for children born after ART who emigrated or children born to mothers who lived in the England & Wales but travelled abroad for ART treatment, although European estimates of cross-border reproductive care activity suggests that the number of children to whom either of these applies is be small (69). Due to lack of maternal data, it was not feasible to identify and exclude NCP controls that were part of a triplet or higher order birth. However, the number of such individuals is estimated to be small as the incidence of naturally occurring triplets is in the range of 1:5000-1:9000 (42).

Lastly, an important limitation of the current cohort is that it finishes in 2009. Given that ART technologies have advanced rapidly in the last 10 years, there is an urgent need to continue to

For peer review only

prospectively monitor the next cohort of children from 2010 onwards. Two recent UK studies (51, 60) have suggested that BWs from both fresh and frozen transfers are increasing with time and may be different in the post 2010 cohort, suggesting that there may potentially be differences (of unknown origin) in the health outcomes of babies born more recently.

COLLABORATION

De-identified linked cohort data can be accessed from the Human Fertilisation and Embryology Authority and NHS Digital where it will be held with restricted access. Specific ethical approval from the Research Ethics Committee (REC) and the Confidentiality Advisory Group (CAG) of the Health Research Authority (HRA) will be required for access.

CONCLUSION

The current study carried out a bespoke record linkage that generated a new child cohort for use in exploring the relationship between conception via ART and short- and long-term health outcomes in offspring and enabling future linkage studies to other developmental and health data sources.

FURTHER DETAILS

Contributors: In line with the ICMJE authorship guidelines, authors AGS, SAR, DB, SMN, and DAL made substantial contributions to the conception or design of the work. Author MP undertook data preparation and provided statistical analysis, with SAR and JG providing statistical oversight. Authors MP, SAR, JG, DB, SMN, DAL, BL and AGS made substantial contributions to the interpretation of data. All authors contributed to the drafting of the manuscript, and/or the revising of the manuscript. All authors have given final approval of the version to be published, and agree to its accuracy.

Funding: This work was supported by the Medical Research Council [Grant number MR/L020335/1].

Acknowledgement: The authors would like to acknowledge the NIHR Great Ormond Street Hospital Biomedical Research Centre (BRC) for their support.

Competing interest: None declared.

Ethical considerations: Ethical approval and Section 251 support were obtained from the NHS Research Ethics Committee and Confidentiality Advisory Group, respectively. Additional data access

For peer review only

permissions were sought from the HFEA Register Research Panel, ONS Micro-Data Release board, and the NHS Digital Medical Register. All researchers with data access underwent NHS Digital Data Security Awareness and ONS Safe Researcher accreditation.

Provenance and peer review: Not commissioned; externally peer reviewed.

Data sharing statement: De-identified linked cohort data can be accessed from the Human Fertilisation and Embryology Authority and NHS Digital where it will be held with restricted access. Specific ethical approval from the Research Ethics Committee (REC) and the Confidentiality Advisory Group (CAG) of the Health Research Authority (HRA) will be required for access.

For peer review only

REFERENCES

(1) Lancaster P, de Mouzon J. Global Committee Reproductive ART Surveillance: Monitoring Technologies Assisted The (ICMART) International. *Assisted Reproductive Technology Surveillance*. 2019:101.
2) Barker DJ. Fetal programming of coronary heart disease. *Trends in Endocrinology & Metabolism*. 2002;13(9):364-8.
(3) Lane M, Robker RL, Robertson SA. Parenting from before conception. *Science*. 2014;345(6198):756-60.
(4) Fleming TP, Watkins AJ, Velazquez MA, Mathers JC, Prentice AM, Stephenson J, et al. Origins of lifetime health around the time of conception: causes and consequences. *Lancet*. 2018;391(10132):1842-52.
(5) Fisher-Jeffes L, Banerjee I, Sutcliffe A. Parents' concerns regarding their ART children. *Reproduction*. 2006;131(2):389-94.
(6) Källén B, Finnström O, Nygren KG, Olausson PO. In vitro fertilization in Sweden: child morbidity including cancer risk. *Fertility and Sterility*. 2005;84(3):605-10.
(7) Schieve LA, Rasmussen SA, Buck GM, Schendel DE, Reynolds MA, Wright VC. Are children born after assisted reproductive technology at increased risk for adverse health outcomes? *Obstetrics Gynecology*. 2004;103(6):1154-63.
(8) Reddy UM, Wapner RJ, Rebar RW, Tasca RJ. Infertility, assisted reproductive technology, and adverse pregnancy outcomes: executive summary of a National Institute of Child Health and Human Development workshop. *Obstetrics Gynecology*. 2007;109(4):967-77.
(9) Pandey S, Shetty A, Hamilton M, Bhattacharya S, Maheshwari A. Obstetric and perinatal outcomes in singleton pregnancies resulting from IVF/ICSI: a systematic review and meta-analysis. *Human Reproduction Update*. 2012;18(5):485-503.
(10) Gnoth C, Godehardt E, Frank-Herrmann P, Friol K, Tigges J, Freundl G. Definition and prevalence of subfertility and infertility. *Human Reproduction*. 2005;20(5):1144-7.
(11) Ceelen M, van Weissenbruch MM, Prein J, Smit JJ, Vermeiden JP, Spreeuwenberg M, et al. Growth during infancy and early childhood in relation to blood pressure and body fat measures at age 8-18 years of IVF children and spontaneously conceived controls born to subfertile parents. *Human Reproduction*. 2009;24(11):2788-95.
(12) Ceelen M, van Weissenbruch MM, Vermeiden JP, van Leeuwen FE, Delemarre-van de Waal HA. Pubertal development in children and adolescents born after IVF and spontaneous conception. *Human Reproduction*. 2008;23(12):2791-8.
(13) Dumoulin JC, Land JA, Van Montfoort AP, Nelissen EC, Coonen E, Derhaag JG, et al. Effect of in vitro culture of human embryos on birthweight of newborns. *Human Reproduction*. 2010;25(3):605-12.
(14) Van Montfoort AP, Hanssen LL, de Sutter P, Viville S, Geraedts JP, de Boer P. Assisted reproduction treatment and epigenetic inheritance. *Human Reproduction Update*. 2012;18(2):171-97.
(15) Stormlund S, Sopa N, Zedeler A, Bogstad J, Prætorius L, Nielsen HS, et al. Freeze-all versus fresh blastocyst transfer strategy during in vitro fertilisation in women with regular menstrual cycles: multicentre randomised controlled trial. *British Medical Journal*. 2020;370:m2519.
(16) Sandin S, Nygren KG, Iliadou A, Hultman CM, Reichenberg A. Autism and Mental Retardation Among Offspring Born After In Vitro Fertilization. *Journal of the American Medical Association*. 2013;310(1):75-84.
(17) Bay B, Mortensen EL, Hvidtjørn D, Kesmodel US. Fertility treatment and risk of childhood and adolescent mental disorders: register based cohort study. *British Medical Journal*. 2013;347:f3978.
(18) Davies MJ, Moore VM, Willson KJ, Van Essen P, Priest K, Scott H, et al. Reproductive Technologies and the Risk of Birth Defects. *New England Journal of Medicine*. 2012;366(19):1803-13.

For peer review only

(19) Opdahl S, Henningsen A-KA, Bergh C, Gissler M, Romundstad LB, Petzold M, et al. Data Resource Profile: Committee of Nordic Assisted Reproductive Technology and Safety (CoNARTaS) cohort. *International Journal of Epidemiology*. 2019;49(2):365-6f.
(20) Henningsen AK, Romundstad LB, Gissler M, Nygren K-G, Lidegaard Ø, Skjaerven R, et al. Infant and maternal health monitoring using a combined Nordic database on ART and safety. *Acta Obstetricia et Gynecologica Scandinavica*. 2011;90(7):683-91.
(21) Wennberg AL, Opdahl S, Bergh C, Henningsen A-KA, Gissler M, Romundstad LB, et al. Effect of maternal age on maternal and neonatal outcomes after assisted reproductive technology. *Fertility and sterility*. 2016;106(5):1142-9. e14.
(22) Finnström O, Källén B, Lindam A, Nilsson E, Nygren KG, Olausson PO. Maternal and child outcome after in vitro fertilization--a review of 25 years of population-based data from Sweden. *Acta Obstetricia et Gynecologica Scandinavica*. 2011;90(5):494-500.
(23) Henningsen AK, Pinborg A, Lidegaard Ø, Vestergaard C, Forman JL, Andersen AN. Perinatal outcome of singleton siblings born after assisted reproductive technology and spontaneous conception: Danish national sibling-cohort study. *Fertility and Sterility*. 2011;95(3):959-63.
(24) Kalra SK, Ratcliffe SJ, Barnhart KT, Coutifaris C. Extended embryo culture and an increased risk of preterm delivery. *Obstetrics Gynecology*. 2012;120(1):69-75.
(25) Romundstad LB, Romundstad PR, Sunde A, von Düring V, Skjaerven R, Gunnell D, et al. Effects of technology or maternal factors on perinatal outcome after assisted fertilisation: a population-based cohort study. *Lancet*. 2008;372(9640):737-43.
(26) Lawlor DA, Tilling K, Davey Smith G. Triangulation in aetiological epidemiology. *International Journal of Epidemiology*. 2017;45(6):1866-86.
(27) Romundstad LB, Romundstad PR, Sunde A, von Düring V, Skjaerven R, Vatten LJ. Increased risk of placenta previa in pregnancies following IVF/ICSI; a comparison of ART and non-ART pregnancies in the same mother. *Human Reproduction*. 2006;21(9):2353-8.
(28) Human Fertilisation and Embryology Authority. *Fertility treatment 2014–2016 Trends and figures*. London: HFEA. 2018. URL: https://www.hfea.gov.uk/media/3188/hfea-fertility-trends-and-figures-2014-2016.pdf
(29) *Human Fertilisation and Embryology Act 1990* (UK). URL: https://www.legislation.gov.uk/ukpga/1990/37/contents
(30) *Human Fertilisation and Embryology Privacy Policy*. 2018. (UK) URL: https://www.hfea.gov.uk/privacy-policy-cookies-and-accessibility/privacy-policy/
(31) *Human Fertilisation & Embryology Authority* (2019). URL: https://www.hfea.gov.uk/.
(32) Templeton A, Morris JK, Parslow W. Factors that affect outcome of in-vitro fertilisation treatment. *Lancet*. 1996;348(9039):1402-6.
(33) Nelson SM, Lawlor DA. Predicting Live Birth, Preterm Delivery, and Low Birth Weight in Infants Born from In Vitro Fertilisation: A Prospective Study of 144,018 Treatment Cycles. *PLOS Medicine*. 2011;8(1):e1000386.
(34) Maheshwari A, Raja EA, Bhattacharya S. Obstetric and perinatal outcomes after either fresh or thawed frozen embryo transfer: an analysis of 112,432 singleton pregnancies recorded in the Human Fertilisation and Embryology Authority anonymized dataset. *Fertility and Sterility*. 2016;106(7):1703-8.
(35) Kamath MS, Antonisamy B, Selliah HY, Sunkara SK. Perinatal outcomes of singleton live births with and without vanishing twin following transfer of multiple embryos: analysis of 113 784 singleton live births. *Human Reproduction*. 2018;33(11):2018-22.
(36) Marconi N, Raja EA, Bhattacharya S, Maheshwari A. Perinatal outcomes in singleton live births after fresh blastocyst-stage embryo transfer: a retrospective analysis of 67 147 IVF/ICSI cycles. *Human Reproduction*. 2019;34(9):1716-25.
(37) Castillo CM, Johnstone ED, Horne G, Falconer DA, Troup SA, Cutting R, et al. Associations of IVF singleton birthweight and gestation with clinical treatment and laboratory factors: a multicentre cohort study. *Human Reproduction*. 2020;35(12):2860-70.

For peer review only

(38) Roberts S, McGowan L, Hirst W, Brison D, Vail A, Lieberman B. Towards single embryo transfer? Modelling clinical outcomes of potential treatment choices using multiple data sources: predictive models and patient perspectives. *Health Technology Assessment*. 2010;14(38):1-237.
(39) Hann M, Roberts SA, D'Souza SW, Clayton P, Macklon N, Brison DR. The growth of assisted reproductive treatment-conceived children from birth to 5 years: a national cohort study. BMC Medicine. 2018;16(1):224.
(40) *Human Fertilisation & Embryology Act 2008*. (UK). URL: https://www.legislation.gov.uk/ukpga/2008/22/contents
(41) Williams CL, Bunch KJ, Stiller CA, Murphy MFG, Botting BJ, Wallace WH, et al. Cancer Risk among Children Born after Assisted Conception. *New England Journal of Medicine*. 2013;369(19):1819-27.
(42) Office for National Statistics. *User guide to birth statistics.*2019. URL: https://www.ons.gov.uk/peoplepopulationandcommunity/birthsdeathsandmarriages/livebirths/methodologies/userguidetobirthstatistics
(43) NHS Digital. *Hospital Episode Statistics*. URL: https://digital.nhs.uk/data-and-information/data-tools-and-services/data-services/hospital-episode-statistics.
(44) Boyd A, Cornish R, Johnson L, Simmonds S, Syddall H, Westbury L, Cooper C, Macleod J. *Understanding Hospital Episode Statistics (HES)*. London, UK: CLOSER. 2017.
(45) Herbert A, Wijlaars L, Zylbersztejn A, Cromwell D, Hardelid P. Data Resource Profile: Hospital Episode Statistics Admitted Patient Care (HES APC). *International Journal of Epidemiology*. 2017;46(4):1093-i.
(46) Zegers-Hochschild F, Adamson GD, Dyer S, Racowsky C, de Mouzon J, Sokol R, et al. The International Glossary on Infertility and Fertility Care, 2017. *Fertility and Sterility*. 2017;108(3):393-406.
(47) Williams C, Jones M, Swerdlow A, Botting B, Davies M, Jacobs I, et al. Risks of ovarian, breast, and corpus uteri cancer in women treated with assisted reproductive technology in Great Britain, 1991-2010: data linkage study including 2.2 million person years of observation. *British Medical Journal*. 2018;362:k2644.
(48) Yokoyama Y, Shimizu T, Hayakawa K. Incidence of handicaps in multiple births and associated factors. *Acta geneticae medicae et gemellologiae: twin research*. 1995;44(2):81-91.
(49) Doyle P. The outcome of multiple pregnancy. *Human Reproduction*. 1996;11(suppl_4):110-20.
(50) Elm Ev, Altman DG, Egger M, Pocock SJ, Gøtzsche PC, Vandenbroucke JP. Strengthening the reporting of observational studies in epidemiology (STROBE) statement: guidelines for reporting observational studies. *British Medical Journal*. 2007;335(7624):806-8.
(51) Castillo CM, Horne G, Fitzgerald CT, Johnstone ED, Brison DR, Roberts SA. The impact of IVF on birthweight from 1991 to 2015: a cross-sectional study. *Human Reproduction*. 2019;34(5):920-31.
(52) Pelkonen S, Koivunen R, Gissler M, Nuojua-Huttunen S, Suikkari A-M, Hydén-Granskog C, et al. Perinatal outcome of children born after frozen and fresh embryo transfer: the Finnish cohort study 1995–2006. *Human Reproduction*. 2010;25(4):914-23.
(53) Roy TK, Bradley CK, Bowman MC, McArthur SJ. Single-embryo transfer of vitrified-warmed blastocysts yields equivalent live-birth rates and improved neonatal outcomes compared with fresh transfers. *Fertility and Sterility*. 2014;101(5):1294-301. e2.
(54) Litzky JF, Boulet SL, Esfandiari N, Zhang Y, Kissin DM, Theiler RN, et al. Effect of frozen/thawed embryo transfer on birthweight, macrosomia, and low birthweight rates in US singleton infants. *American Journal of Obstetrics and Gynecology*. 2018;218(4):433.e1-.e10.
(55) Berntsen S, Pinborg A. Large for gestational age and macrosomia in singletons born after frozen/thawed embryo transfer (FET) in assisted reproductive technology (ART). *Birth Defects Res*earch. 2018;110(8):630-43.
(56) Terho AM, Pelkonen S, Opdahl S, Romundstad LB, Bergh C, Wennerholm UB, et al. High birth weight and large-for-gestational-age in singletons born after frozen compared to fresh embryo transfer, by gestational week: a Nordic register study from the CoNARTaS group. *Human Reproduction*. 2021.

For peer review only

(57) Elias FTS, Weber-Adrian D, Pudwell J, Carter J, Walker M, Gaudet L, et al. Neonatal outcomes in singleton pregnancies conceived by fresh or frozen embryo transfer compared to spontaneous conceptions: a systematic review and meta-analysis. *Archives of Gynecology and Obstetrics*. 2020;302(1):31-45.
(58) Maheshwari A, Pandey S, Amalraj Raja E, Shetty A, Hamilton M, Bhattacharya S. Is frozen embryo transfer better for mothers and babies? Can cumulative meta-analysis provide a definitive answer? *Human Reproduction Update*. 2018;24(1):35-58.
(59) Sha T, Yin X, Cheng W, Massey IY. Pregnancy-related complications and perinatal outcomes resulting from transfer of cryopreserved versus fresh embryos in vitro fertilization: a meta-analysis. *Fertility and Sterility*. 2018;109(2):330-42.e9.
(60) Castillo CM, Harper J, Roberts SA, O'Neill HC, Johnstone ED, Brison DR. The impact of selected embryo culture conditions on ART treatment cycle outcomes: a UK national study. *Human Reproduction Open*. 2020;2020(1):hoz031.
(61) Berntsen S, Söderström-Anttila V, Wennerholm UB, Laivuori H, Loft A, Oldereid NB, et al. The health of children conceived by ART: 'the chicken or the egg?'. *Human Reproduction Update*. 2019;25(2):137-58.
(62) Luke B, Brown MB, Wantman E, Stern JE, Toner JP, Coddington CC, 3rd. Increased risk of large-for-gestational age birthweight in singleton siblings conceived with in vitro fertilization in frozen versus fresh cycles. *Journal of Assisted Reproduction and Genetics*. 2017;34(2):191-200.
(63) Henningsen AK, Pinborg A. Birth and perinatal outcomes and complications for babies conceived following ART. *Seminars in Fetal & Neonatal Medicine*. 2014;19(4):234-8.
(64) Pinborg A, editor To transfer fresh or thawed embryos? *Seminars in Reproductive Medicine*; 2012: Thieme Medical Publishers.
(65) Kimber SJ, Sneddon SF, Bloor DJ, El-Bareg AM, Hawkhead JA, Metcalfe AD, et al. Expression of genes involved in early cell fate decisions in human embryos and their regulation by growth factors. *Reproduction*. 2008;135(5):635-47.
(66) Goldstein H. Factors related to birth weight and perinatal mortality. *British Medical Bulletin*. 1981;37(3):259-64.
(67) Harron K, Dibben C, Boyd J, Hjern A, Azimaee M, Barreto ML, et al. Challenges in administrative data linkage for research. *Big Data & Society*. 2017;4(2):2053951717745678.
(68) Spencer SA, Davies MP. Hospital episode statistics: improving the quality and value of hospital data: a national internet e-survey of hospital consultants. *BMJ Open*. 2012;2(6).
(69) Shenfield F, de Mouzon J, Pennings G, Ferraretti AP, Andersen AN, de Wert G, et al. Cross border reproductive care in six European countries. *Human Reproduction*. 2010;25(6):1361-8.

For peer review only

Figure 1: Flowchart showing linkage 1 (HFEA-ONS)

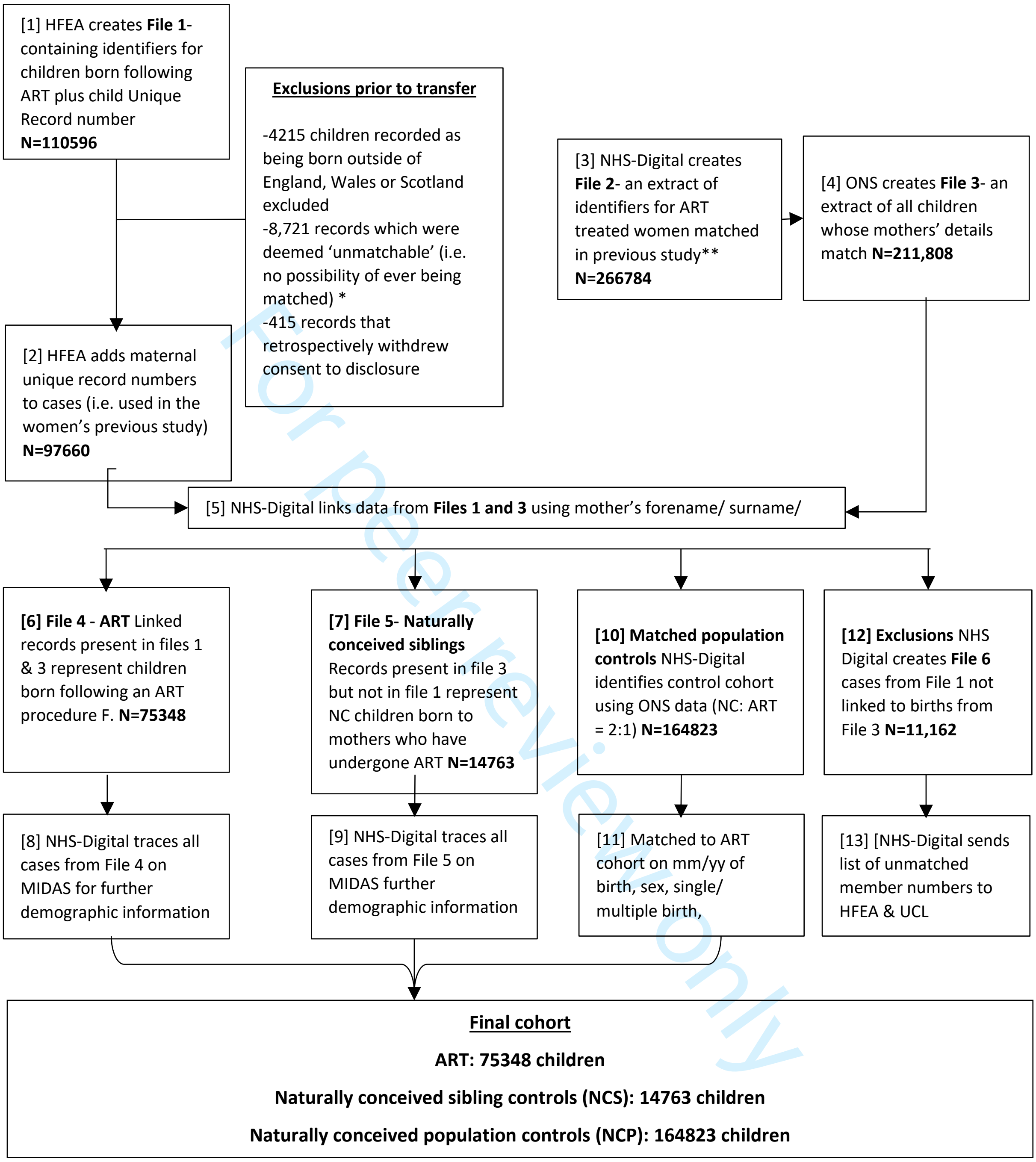

*as they were a) births outside of England/ Wales; b) births before 1993 (when ONS systems were automated and thus the date from which linkage is possible to ONS records); and c) to mothers which were not included in file 2 (as it was not possible to identify them on NHS-Digital systems previously- 'women's study').
** Please see Supplementary figure S2 for cohort flow

For peer review only

Figure 2: Flowchart showing linkage 2 (HFEA-ONS-HES)

[14] NHS Digital produces current status report on all ART, NCS and NCP controls (as produced in linkage 1) and provides de-identified output plus non identifiable deprivation score to UCL.

↓

[15] NHS Digital links cohort data to HES data and provides de-identified output to UCL

↓

[16] UCL interacts with HFEA to obtain fertility treatment clinical data and parental data per matched group member.

UCL match up NHS Digital and clinical HES information to HFEA information using unique record number.

↓

[17] UCL excludes records of children born before 1st April 1997 to coincide with start of HES monitoring.

UCL excludes triplets and higher order births from all groups (along with associated ART, siblings and matched controls)

↓

[18] NHS Digital produces file 4 for each group (identifiable details as below).

- For the ART group, file 4 will be securely transported to the HFEA and stored there securely with highly restricted access.
- For the NCS and NCP groups, file 4 will be securely stored at NHS Digital again with highly restricted access.

↓

Final sub-cohort

HES-ART: 63877

Naturally conceived siblings controls (HES-NCS): 11343

Naturally conceived population controls (HES-NCP): 127544

HES: Hospital Episode Statistics database; UCL: University College London; HFEA: Human Fertilization and Embryology Authority

For peer review only

S1: Completeness of variables (for the purpose of linkage and analysis) on the HFEA register

Variable	Useable* entry	Entry 'Null'	No entry	Other**	% useable out of total no. of entries
DOB	110,204	374	0	18	99.6%
Sex of Child<br>*Male*<br>*Female*	110,484<br>*56,265*<br>*54,219*	112	0	0	99.9%
BW	109, 157	534	0	905 records with BW <600g ***	98.7%
Child's Surname	12,332	97,671	0	593	11.2%
Child's Forename	11,402	97,670	0	1524	10.3%
Child's Town of Birth	70,738	39,689	61	108	64.0%
Child's District of Birth	18,833	91,452	298	13	17.0%
Child's Town ***or*** District of Birth	71,650	38,787	51	108	64.8%
Country of Birth	67554	38,861	0	4181 cases born outside of Great Britain	61.1%
Mother's Surname	110,596	0	0	0	100%
Mother's Forename	110,588	0	8	0	100.0%
Mother's Surname at Birth	51,237	58,714	209	436	46.3%
Mother's Forename at Birth	3,323	106,895	377	1	3.0%
Mother's DOB	110,569	1	0	26	100.0%
Father's Surname	110,450	138	8	0	99.9%
Father's Forename	110,384	202	8	2	99.8%
Father's DOB	110,282	257	0	57	99.7%
Mother's Town or District of Birth	62,505	47,045	0	1046	56.5%
Mother's Country of Birth	64,396	46,190	0	10	58.2%
Father's Town or District of Birth	61,143	48,354	0	1099	55.3%
Father's Country of Birth	63,100	47,485	0	11	57.1%
Treatment Cycle Start Date	110,596	0	0	0	100%
Treatment Centre No/ Name	110,594	0	0	2	100.0%

* 'Useable' data refers to records which contain data that appears useable in some analyses, including initial quality control analyses.

** 'Other' refers to unusable information for the variable in question, most commonly variations of 'Not known', 'Unknown' etc.

***905 records with documented BWs less than 600g were considered as invalid information for the purpose of deterministic linkage. Whilst a small number of these BWs (<600g) are likely to be correct, many more are likely to be data errors within the HFEA register. All actual recorded BWs were used when manually viewing potential matches.

This table was shared with us by HFEA at the start of the study.

For peer review only

S2- Flow diagram showing derivation of File 2 in Figure 1.

302, 487 Human Fertilisation & Embryology (HFEA) records relating to women treated with ART, 1991-2010, were available for linkage. These were transferred to NHS-digital & National Records of Scotland for linkage to NHS central registries of England & Wales and Scotland (EWS).

13,017 duplicates
- 11,290 - 2 or more distinct unique record numbers relating to the same individual
- 1,727 - duplicate unique record numbers.
- 1,722: Exact duplicate unique record numbers
- 5: Both had an exact duplicate unique record numbers and two distinct unique record numbers

22,683 ‘No trace’ on linkage to NHSCR
- 8, 854 - HFEA recorded address outside England, Wales and Scotland (EWS) (no possibility of linkage to NHSCR)
- 13,829 - EWS based women, no trace on NHSCR

3 records - noted to have possible linkage error after data quality checks

266,784 of 280,616 eligible records successfully linked.
- ***<u>Linkage rate = 95.07% *</u>***

* Denominator excludes duplicate records and records of women based overseas who had treatment in the UK.

242 records - contained no clinical fertility data

122 records - embarked from EWS before treatment date, thus no outcome data available

266,420 of 280,616 records successfully linked and containing both fertility and outcome data.
- 94.94%

1,678 records **-** treatment date missing

52 records – DOB missing

8,904 - Women had a cancer diagnosis prior to the first calendar year of treatment.

255,786 of 280,616 potentially eligible records entered into the cohort analysis
- 91.15%

S3: Order of pregnancy (HFEA-ONS cohort)

No. of pregnancies	Order of pregnancy	ART		Spontaneously conceived siblings	
		Singleton pregnancy	Twin Pregnancy	Singleton pregnancy	Twin Pregnancy
**2 (n=8441)**	1	5135	1182	2095	29
	2	1596	528	6161	156
**3 (n=1336)**	1	918	107	304	7
	2	453	96	777	10
	3	126	26	1155	29
**4 (n=125)**	1	83	9	33	0
	2	42	5	77	1
	3	19	6	99	1
	4	7	4	113	1
**5 (n=12)**	1	8	1	2	1
	2	5	1	6	0
	3	2	0	10	0
	4	0	0	12	0
	5	0	0	12	0
**6 (n=1)**	1	1	0	0	0
	2	0	0	1	0
	3	0	0	1	0
	4	0	0	1	0
	5	0	0	1	0
	6	0	0	1	0
**7 (n=1)**	1	0	1	0	0
	2	0	0	1	0
	3	0	0	1	1
	4	0	0	0	0
	5	0	0	1	0
	6	0	0	1	0
	7	0	0	1	0

For peer review only

S4: Multiple regression coefficients for adjusted mean BW- ART vs NCP (HFEA-ONS)

Variables	BW Coefficient	95% Confidence interval
Embryo transfer status		
Fresh	-131.4	(-139.5 - -123.4)
Frozen	35.24	(18.97 - 51.52)
Sex		
Male	119.1	(112.7 - 125.5)
Maternal age group		
25-29	66.68	(55.89 - 77.48)
30-34	111.9	(102.2 - 121.6)
35-39	129.2	(118.7 - 139.7)
40-44	132.3	(118.0 - 146.5)
>=45	72.66	(-13.46 - 158.8)
**Constant**	3,215	(3,207 - 3,223)
**Observations**	129,304	
**R-squared**	0.021	

For peer review only

S5: Multiple regression coefficients for adjusted mean BW- sART vs NCS (HFEA-ONS)

VARIABLES	BW coefficient	95% Confidence interval
Embryo transfer status		
Fresh	-54.69	(-72.64 - -36.73)
Frozen	138.6	(101.3 - 175.9)
Sex		
Male	115.6	(97.78 - 133.4)
Maternal age group		
25-29	-43.23	(-114.2 – 27.74)
30-34	-98.50	(-176.5 - -20.46)
35-39	-117.1	(-206.8 - -27.41)
40-44	-161.8	(-266.1 - -57.52)
>=45	-198.7	(-409.2 – 11.79)
Order of pregnancy		
2	165.6	(142.2 – 188.9)
>=3	237.0	(194.4 – 279.6)
**Constant**	3,337	(3,263 - 3,411)
**Observations**	22,086	
**R-squared**	0.081	

For peer review only

S6: Multiple regression coefficients for adjusted mean BW- ART vs NCP (HFEA-ONS-HES cohort)

Variables	BW coefficient	95% confidence interval
Embryo transfer status		
Fresh	-152.2	(-162.2 - -142.3)
Frozen	2.855	(-16.61 - 22.32)
Sex		
Male	120.6	(112.7 - 128.6)
Maternal age group		
25-29	57.25	(43.52 - 70.99)
30-34	86.40	(73.80 - 99.00)
35-39	91.20	(77.62 - 104.8)
40-44	98.57	(80.37 - 116.8)
45-49	4.103	(-63.56 - 71.77)
Missing	94.69	(60.19 - 129.2)
IMD at earliest appointment		
2	25.95	(8.336 - 43.57)
3	56.66	(38.69 - 74.64)
4	58.78	(40.66 - 76.91)
5	75.13	(56.93 - 93.34)
6	93.33	(75.06 - 111.6)
7	93.21	(75.17 - 111.2)
8	111.8	(93.76 - 129.8)
9	117.3	(99.48 - 135.1)
10	134.7	(116.5 - 152.8)
Derived ethnicity		
Asian/Asian British	-247.8	(-264.2 - -231.4)
Black/African/Caribbean/Black British	-138.2	(-161.2 - -115.2)
Chinese	-120.1	(-195.7 - -44.43)
Mixed/multiple ethnic groups	-66.13	(-94.92 - -37.33)
Not stated/Not known	5.034	(-5.060 - 15.13)
Other ethnic group	-49.19	(-82.25 - -16.13)
**Constant**	3,177	(3,162 - 3,192)
**Observations**	85,366	
**R-squared**	0.040	

For peer review only

S7: Multiple regression coefficients for adjusted mean BW- sART vs NCS (HFEA-ONS-HES cohort)

Variables	BW coefficient	95% Confidence interval
Embryo transfer status		
Fresh	-57.01	(-75.79 - -38.23)
Frozen	152.1	(113.5 - 190.7)
Sex		
Male	115.6	(96.95 – 134.2)
Maternal age group		
25-29	-28.73	(-104.3 - -46.84)
30-34	-81.27	(-164.9 – 2.335)
35-39	-96.01	(-192.0 - -0.010)
40-44	-135.4	(-246.9 - -23.89)
>=45	-141.6	(-308.8 - -25.69)
Order of pregnancy		
2	152.2	(127.8 – 176.6)
>=3	229.6	(184.3 – 274.8)
**Constant**	3,332	(3,252 - 3,412)
**Observations**	19180	
**R-squared**	0.09	

For peer review only

For peer review only

Reporting checklist for cohort study.

Based on the STROBE cohort guidelines.

Instructions to authors

Complete this checklist by entering the page numbers from your manuscript where readers will find each of the items listed below.

Your article may not currently address all the items on the checklist. Please modify your text to include the missing information. If you are certain that an item does not apply, please write "n/a" and provide a short explanation.

Upload your completed checklist as an extra file when you submit to a journal.

In your methods section, say that you used the STROBE cohort reporting guidelines, and cite them as:

von Elm E, Altman DG, Egger M, Pocock SJ, Gotzsche PC, Vandenbroucke JP. The Strengthening the Reporting of Observational Studies in Epidemiology (STROBE) Statement: guidelines for reporting observational studies.

		Reporting Item	Page Number
**Title and abstract**			
Title	#1a	Indicate the study's design with a commonly used term in the title or the abstract	1 & 2

For peer review only

Abstract	#1b	Provide in the abstract an informative and balanced summary of what was done and what was found	2
**Introduction**			
Background / rationale	#2	Explain the scientific background and rationale for the investigation being reported	4
Objectives	#3	State specific objectives, including any prespecified hypotheses	5, 6
**Methods**			
Study design	#4	Present key elements of study design early in the paper	6,7,8
Setting	#5	Describe the setting, locations, and relevant dates, including periods of recruitment, exposure, follow-up, and data collection	7, 8
Eligibility criteria	#6a	Give the eligibility criteria, and the sources and methods of selection of participants. Describe methods of follow-up.	7, 8
Eligibility criteria	#6b	For matched studies, give matching criteria and number of exposed and unexposed	7, 8

For peer review only

Variables	#7	Clearly define all outcomes, exposures, predictors, potential confounders, and effect modifiers. Give diagnostic criteria, if applicable	7, 8
Data sources / measurement	#8	For each variable of interest give sources of data and details of methods of assessment (measurement). Describe comparability of assessment methods if there is more than one group. Give information separately for for exposed and unexposed groups if applicable.	6,7
Bias	#9	Describe any efforts to address potential sources of bias	10<br><br>Creation of two control cohorts to minimize bias
Study size	#10	Explain how the study size was arrived at	N/A<br><br>Population-based study.
Quantitative variables	#11	Explain how quantitative variables were handled in the analyses. If applicable, describe which groupings were chosen, and why	11

For peer review only

Statistical methods	#12a	Describe all statistical methods, including those used to control for confounding	10, 11
Statistical methods	#12b	Describe any methods used to examine subgroups and interactions	11
Statistical methods	#12c	Explain how missing data were addressed	8-11
Statistical methods	#12d	If applicable, explain how loss to follow-up was addressed	N/A
Statistical methods	#12e	Describe any sensitivity analyses	11
**Results**			
Participants	#13a	Report numbers of individuals at each stage of study—eg numbers potentially eligible, examined for eligibility, confirmed eligible, included in the study, completing follow-up, and analysed. Give information separately for for exposed and unexposed groups if applicable.	11, 12, Figure 1 & 2
Participants	#13b	Give reasons for non-participation at each stage	N/A
Participants	#13c	Consider use of a flow diagram	Figure 1 & 2

For peer review only

Descriptive data	#14a	Give characteristics of study participants (eg demographic, clinical, social) and information on exposures and potential confounders. Give information separately for exposed and unexposed groups if applicable.	13, 14, 16 ,17
Descriptive data	#14b	Indicate number of participants with missing data for each variable of interest	13, 14, 16 ,17
Descriptive data	#14c	Summarise follow-up time (eg, average and total amount)	13, 14, 16 ,17
Outcome data	#15	Report numbers of outcome events or summary measures over time. Give information separately for exposed and unexposed groups if applicable.	13, 14, 16 ,17
Main results	#16a	Give unadjusted estimates and, if applicable, confounder-adjusted estimates and their precision (eg, 95% confidence interval). Make clear which confounders were adjusted for and why they were included	17, 18
Main results	#16b	Report category boundaries when continuous variables were categorized	13, 14, 16 ,17

For peer review only

Main results	#16c	If relevant, consider translating estimates of relative risk into absolute risk for a meaningful time period	N/A
Other analyses	#17	Report other analyses done—eg analyses of subgroups and interactions, and sensitivity analyses	N/A
**Discussion**			
Key results	#18	Summarise key results with reference to study objectives	18
Limitations	#19	Discuss limitations of the study, taking into account sources of potential bias or imprecision. Discuss both direction and magnitude of any potential bias.	20
Interpretation	#20	Give a cautious overall interpretation considering objectives, limitations, multiplicity of analyses, results from similar studies, and other relevant evidence.	18, 19
Generalisability	#21	Discuss the generalisability (external validity) of the study results	20
**Other Information**			

For peer review only

Funding	#22	Give the source of funding and the role of the funders for the present study and, if applicable, for the original study on which the present article is based	22

None The STROBE checklist is distributed under the terms of the Creative Commons Attribution License CC-BY. This checklist can be completed online using https://www.goodreports.org/, a tool made by the EQUATOR Network in collaboration with Penelope.ai

For peer review only

BMJ Open

Cohort Profile: A national, population based cohort of children born after assisted conception in the UK (1992-2009) - Methodology and birthweight analysis

Journal:	*BMJ Open*
Manuscript ID	bmjopen-2021-050931.R1
Article Type:	Cohort profile
Date Submitted by the Author:	11-May-2021
Complete List of Authors:	Purkayastha, Mitana; UCL Great Ormond Street Institute of Child Health Population Policy and Practice<br>Roberts, Stephen; University of Manchester, Centre for Biostatistics,Institute of Population Health<br>Gardiner, Julian; University of Oxford, Education; UCL Great Ormond Street Institute of Child Health Population Policy and Practice,<br>Brison, Daniel; Central Manchester University Hospitals NHS Foundation Trust, Department of Reproductive Medicine<br>Nelson, Scott; University of Glasgow, School of Medicine<br>Lawlor, Deborah; University of Bristol Bristol Population Health Science Institute, MRC Integrative Epidemiology Unit<br>Luke, Barbara ; Michigan State University,<br>Sutcliffe, Alastair; UCL Great Ormond Street Institute of Child Health Population Policy and Practice
**Primary Subject Heading**:	Paediatrics
Secondary Subject Heading:	Epidemiology, Reproductive medicine
Keywords:	Subfertility < GYNAECOLOGY, EPIDEMIOLOGY, PAEDIATRICS

SCHOLARONE™
Manuscripts

I, the Submitting Author has the right to grant and does grant on behalf of all authors of the Work (as defined in the below author licence), an exclusive licence and/or a non-exclusive licence for contributions from authors who are: i) UK Crown employees; ii) where BMJ has agreed a CC-BY licence shall apply, and/or iii) in accordance with the terms applicable for US Federal Government officers or employees acting as part of their official duties; on a worldwide, perpetual, irrevocable, royalty-free basis to BMJ Publishing Group Ltd ("BMJ") its licensees and where the relevant Journal is co-owned by BMJ to the co-owners of the Journal, to publish the Work in this journal and any other BMJ products and to exploit all rights, as set out in our licence.

The Submitting Author accepts and understands that any supply made under these terms is made by BMJ to the Submitting Author unless you are acting as an employee on behalf of your employer or a postgraduate student of an affiliated institution which is paying any applicable article publishing charge ("APC") for Open Access articles. Where the Submitting Author wishes to make the Work available on an Open Access basis (and intends to pay the relevant APC), the terms of reuse of such Open Access shall be governed by a Creative Commons licence – details of these licences and which Creative Commons licence will apply to this Work are set out in our licence referred to above.

Other than as permitted in any relevant BMJ Author's Self Archiving Policies, I confirm this Work has not been accepted for publication elsewhere, is not being considered for publication elsewhere and does not duplicate material already published. I confirm all authors consent to publication of this Work and authorise the granting of this licence.

For peer review only

Cohort Profile: A national, population based cohort of children born after assisted conception in the UK (1992-2009) - Methodology and birthweight analysis

Purkayastha M[a] [ORCID ID: https://orcid.org/0000-0002-1870-8838; email: m.purkayastha@ucl.ac.uk]

Roberts S.A[b] [ORCID ID: https://orcid.org/0000-0002-7477-7731; email: Steve.Roberts@manchester.ac.uk]

Gardiner J[c] [ORCID ID: https://orcid.org/0000-0001-8988-3812; email: julian.gardiner@education.ox.ac.uk]

Brison D.R[d] [ORCID ID: https://orcid.org/0000-0002-4307-1293; email: daniel.brison@manchester.ac.uk]

Nelson S.M[e] [ORCID ID: https://orcid.org/0000-0002-9099-3047; email: Scott.Nelson@glasgow.ac.uk]

Lawlor D.A[f] [ORCID ID: https://orcid.org/0000-0002-6793-2262; email: D.A.Lawlor@bristol.ac.uk]

Luke B[g] [ORCID ID: https://orcid.org/0000-0002-0405-8133; email: barbara.luke@hc.msu.edu]

Sutcliffe A.G[a] [ORCID ID: https://orcid.org/0000-0001-8542-6155; email: a.sutcliffe@ucl.ac.uk]

Author Affiliations:

[a] Great Ormond Street Institute of Child Health, University College London

[b] Division of Population Health, Health Services Research & Primary Care, University of Manchester

[c] Department of Education, University of Oxford

[d] Division of Developmental Biology & Medicine, University of Manchester

[e] School of Medicine, Dentistry & Nursing, Reproductive & Maternal Medicine, University of Glasgow

[f] Bristol Population Health Science Institute, University of Bristol

[g] Obstetrics, Gynaecology and Reproductive Biology,College Of Human Medicine, Michigan State University

Corresponding Author:

Mitana Purkayastha

30 Guilford Street

Population, Policy, and Practice

UCL Great Ormond Street Institute of Child Health

London WC1N 1DP

Email: m.purkayastha@ucl.ac.uk

Word count: 6193

For peer review only

ABSTRACT

Purpose: To generate a large cohort of children born after assisted reproductive technology (ART) in the UK between 1992 and 2009, their naturally conceived siblings (NCS), and matched naturally conceived population (NCP) controls and linking this with health outcome data to allow exploration of the effects of ART. The effects of fresh and frozen embryo transfer on birth weight were analysed to test the validity of the cohort.

Participants: Children recorded on the Human Fertilisation & Embryology Authority (HFEA) register as being born after ART between 1992 and 2009, their NCS, and matched NCP controls linked to Office for National Statistics birth registration dataset (HFEA-ONS cohort). This cohort was further linked to the UK Hospital Episode Statistics database to allow monitoring of the child's post-natal health outcomes up to 2015 (HFEA-ONS-HES sub-cohort).

Findings to date: The HFEA-ONS cohort consisted of 75348 children born after non-donor ART carried out in the UK between 1st April 1992 and 31st July 2009 and successfully linked to birth registration records, 14763 NCS and 164823 matched NCP controls. The HFEA-ONS-HES sub-cohort included 63877 ART, 11343 NCS, and 127544 matched NCP controls further linked to health outcome data. The exemplar analysis showed that children born after fresh embryo transfers were lighter (BW difference: -131g, 95%CI: -140, -123) and those born after frozen embryo transfers were heavier (BW difference: 35g, 95%CI: 19, 52) than the NCP controls. The within-sibling analyses were directionally consistent with the population control analyses, but attenuated markedly for the fresh vs natural conception (BW difference: -54g; 95% CI: -72, -36) and increased markedly for the frozen vs natural conception (BW difference: 152g; 95% CI: 113, 190) analyses.

Future plans: To utilise this cohort to explore the relationship between ART conception and short- and long-term health outcomes in offspring.

Keywords: assisted reproductive technology; birth weight; naturally conceived siblings; naturally conceived population controls; record linkage

For peer review only

Strengths and limitations of this study

- Meticulous linkage of robust, routinely collected administrative health data to yield a large cohort that is nationally unique, thus increasing the generalisability, accuracy, and precision of results from subsequent analyses.
- Linkage to the hospital admissions and outpatient database provides long-term mortality and morbidity outcome data on offspring for use in longitudinal research, policy planning and strategic development.
- Identification of naturally conceived siblings as well as matched naturally conceived population controls allows exploration of the association of ART with adverse offspring outcomes while accounting for parental factors related to sub-fertility which may confound these associations.
- Comparison of findings between the two approaches (ART vs naturally conceived population controls & ART vs naturally conceived siblings) mentioned above increases confidence in findings.
- The validity of the cohort was tested by means of an exemplar analysis.

For peer review only

INTRODUCTION

Assisted reproductive technology (ART) usage has increased annually since the first live birth in 1978, with over 8 million children born after ART globally (1). This increased utilization of ART has occurred concurrently with developments in our understanding of the impact of the early life environment on long term health (2-4) and most families with ART conceived children reported potential general health risks to their children as their paramount concern (5, 6), resulting in a concomitant increase in focus on the potential adverse short- and long-term effects on offspring. The inability to distinguish the relative importance of the effects of ART treatment factors and parental sub-fertility is a common limitation of many ART follow-up studies (7-9). This issue has been addressed in prospective cohorts by identifying control populations of children born naturally to parents with established subfertility [different from infertility in terms of the time of unwanted non-conception (10)]. One such well-known cohort provided the first evidence of differences in blood pressure and growth parameters in ART children (11, 12). Further evidence comes from within-ART comparisons showing that early life environment (e.g. embryo culture medium composition) is associated with changes in fetal growth, birth weight (BW) and child growth (13, 14), and studies of embryo cryopreservation have made a similar point more recently (15). These small studies have been essential in both directing and validating the associations identified from large population studies, including prospective cohort studies of ART conceptions only (e.g a Swedish study that followed-up 30,959 children born after ART) (16) or large, record linkage studies (e.g. one South Australian record linkage study compared 6163 ART and 302,811 naturally conceived (NC) offspring, while another study in Denmark compared 33,139 ART children to 555828 NC children from the population) (17, 18). The Committee of Nordic ART and Safety (CoNARTaS), created in 2008, utilised medical registry data from Denmark, Finland, Norway and Sweden to establish a large cohort of children conceived after ART or natural conception as well as women with at least one delivery after ART or natural conception, with the aim of examining long-term health outcomes in children born after ART (19-21).

While these large studies using conventional multivariable approaches to explore the association of ART with adverse offspring outcomes are essential in order to extrapolate effect sizes and risk estimates to the general ART population (22-24), they cannot fully account for parental factors related to sub-fertility which may confound these associations. This has been addressed in record linkage studies that used within sibling analyses (where comparisons are made between ART and their NC siblings) to better control for factors related to sub-fertility and other family confounders under the assumption that these parental factors would be the same (or very similar) within sibling

For peer review only

groups (23, 25). However, such analyses typically have lower statistical power due to restricted numbers, can be biased if there is individual level confounding, and the subset of ART children with siblings may not be representative of the ART population as a whole. Therefore, a comparison of the two approaches is valuable as similar results from both would increase confidence in findings (25-27).

In the United Kingdom, birth rates from IVF treatment have increased by over 85% since 1991, with around one in three treatment cycles now resulting in a birth for patients under 35 (28). It has been compulsory for every fertility clinic in the UK to report details of all treatments carried out to the Human Fertilisation & Embryology Authority (HFEA) since its inception in August 1991 (29, 30, 31). Due to the mandatory nature of reporting, the completeness and quality of data related to some couple characteristics (e.g. age, duration of infertility) and ART procedures is high, enabling research analysing treatment outcomes including success rates and perinatal outcomes such as gestation, BW, and congenital anomalies (32-37). However, the quality of perinatal outcome data available on the HFEA register is questionable as it is patient reported via the ART clinics (38). Hann et al (2018) (39) showed that 1 in 15 (6%) BWs recorded on the HFEA register were incorrect compared to data recorded directly by the delivering maternity unit. Moreover, the HFEA register contains no information on the health outcomes of children beyond the immediate perinatal period.

A change in the law in 2009 (40) made it possible for researchers to use patient identifying data contained in the HFEA register from 1991-2009 for the purpose of linkage to offspring health outcome databases, thus allowing the largest ever population studies of ART childhood cancer (41) and early child growth (39). However, further studies have been limited by extensive data governance requirements and high associated costs, and prospective monitoring of outcomes in this population as they progress from childhood into adulthood has been prevented thus far by the paucity of identifiable information necessary for a variety of linkages.

Consequently, the primary objective of this study was to substantially enhance the research value of the HFEA register by utilising electronic record linkage methodology to establish a cohort of children consisting of those born after ART in the UK between 1992 and 2009, their naturally conceived siblings (NCS) and matched naturally conceived population (NCP) controls to allow exploration of outcomes present on the birth registration dataset and subsequent linkage to other datasets. The secondary objective was to create a sub-cohort of children born after ART in the UK, NCS and matched NCP controls and linking this with information on their postnatal health outcomes up to 2015. These databases will be made available to all researchers (subject to necessary approvals), allowing more precise risk estimates of associations of ART with many potential childhood (and maternal) outcomes. Finally, the third objective of this study was to validate the cohort and sub-

For peer review only

cohort produced by comparing the effects of fresh and frozen embryo transfer versus natural conception on singleton BW. The hypothesis being tested was that children born after frozen embryo transfers would be heavier and those born after fresh embryo transfers would be lighter than those that were naturally conceived. Our previous linkage from the HFEA 1991-2009 cohort to maternity and child growth records confirmed evidence in the field that children born after frozen embryo transfers are heavier and those born after fresh embryo transfers are lighter than those that are naturally conceived (NC), and that these differences in BW are further associated with differences in child growth up to the age of 5 (39). However, this study was only able to examine the subset of ART children born in Scotland and the dataset created was not readily linkable to other datasets.

COHORT DESCRIPTION

Databases used

Human Fertilization & Embryology Authority Register (HFEA): The HFEA, an 'arm's length body' of the Department of Health, acts as an independent regulator of fertility treatment and research using human embryos in the UK (31). All licensed fertility clinics in the UK are required by law to provide information to the HFEA about treatments they carry out and their outcomes, ensuring high levels of data completeness. Only 1500 ART outcomes had not been reported as of 07/01/2020, almost all of which were likely to be from overseas patients who had ART in the UK and returned home for the delivery making it difficult for clinics to follow up (42). Regular quality assurance checks including manual validation of data submissions; regular quality assurance checks on data through the inspection process; publication of non-compliances with data quality issues in inspection reports; and, where relevant, review of quality reports and auditing of clinics with irregular data submissions are also carried out (43).

The HFEA Act 1990 (29) made prospective collection and storage of baseline information and birth outcomes on the HFEA register mandatory, although 'consent for disclosure of information for research' was not collected from patients who underwent treatment at a licensed fertility clinic prior to September 2009. The reliability and completeness of information relating to women who have undergone treatment on the HFEA register is considerably high (approximately 99.9%, 100% & 99.9% completeness for forename, surname, & DOB, respectively; Supplementary table S1), although it contains little identifiable information on children born after ART (e.g 11.2% and 10.3% completeness for child's surname and forename, respectively; Supplementary table S1).

Details of the data accessed from HFEA for this study are discussed below (see Linkage section).

For peer review only

Birth Registration Database*:* In the UK, all births are legally required to be registered by the Local Registration Service in partnership with the General Register Office (GRO) in England and Wales, making it the most complete data source available (44). The birth registration dataset is managed by ONS in England and Wales, while Scottish birth records are held by National Records for Scotland (NRS) who work closely with NHS Digital to ensure daily record transfers between the organisations using a secure and closed electronic system.

This study used the birth registration dataset to identify all children born to women known to have undergone ART as well as their NCS, and details of the data accessed have been discussed below (see Linkage section).

Hospital Episode Statistics (HES): Hospital Episode Statistics (HES) is a data warehouse containing details of all admissions, outpatient appointments and A & E attendances at NHS hospitals in England (45). HES data covers all NHS Clinical Commissioning Groups (CCGs) in England, including private patients treated in NHS hospitals, patients resident outside of England, and care delivered by treatment centres (including those in the independent sector) funded by the NHS (45). Approximately 98–99% of hospital activity in England are estimated to be funded by the NHS (46). Moreover, the HES admitted patient care database covers all births in NHS hospitals, representing approximately 97.3% of births in England, thus enabling creation of nationally representative birth cohorts (47, 48).

Although the HES data warehouse has been operating since 1990 (49), linkage of the HES admitted patient care (APC) episodes longitudinally to the same individuals only commenced in 1997/98 when the patient's NHS number became a mandated return from hospitals (50). Furthermore, linkage of this APC dataset to other HES datasets such as outpatient, A & E, and adult critical care only commenced much later in 2003/2004, 2007/2008 and 2008/2009, respectively (50). HES diagnoses were coded using the International Classification of Diseases version 9 (ICD-9) between April 1989 and March 1995 and ICD-10 thereafter.

This study linked the HES database to the ART children, NCS, and matched NCP control groups, and the data accessed for this purpose have been discussed below (see Linkage section). Although the current paper only uses demographic data [ethnicity; UK census-derived Index of Multiple Deprivation (IMD), the official measure of relative deprivation for small areas or neighbourhoods in the UK (51)] and BW from HES to carry out an exemplar analysis, further analyses of longitudinal health outcomes in these cohorts are already underway and will be published shortly.

For peer review only

Medical Integrated Database and Administration System (MIDAS): MIDAS is a system used by NHS Digital's Data Linkage and Extract Service (DLES) to replace the functionality previously provided by the Central Health Register Information System (CHRIS) application, specifically for the administration of information used within research and data linkage functions. It was developed to provide continuity for the existing list cleaning, flagging, tracing and cohort management services to maintain support for approved research and audit projects, National Cancer Registration and the ONS Longitudinal Study. It contains up to date demographic GP registration data via a daily feed from the Secondary User Service, standard demographic identifiers such as names and addresses, information on patients who have exited from and returned to the NHS, as well as cancer and death registration details. MIDAS can be considered as a window into the Personal Demographics Service database, providing bespoke data extracts in addition to regular patient tracking services. Extracts are provided on an individual as-and-when basis, and are subject to the regular DLES approvals process.

In this study, the cohort produced by Linkage 1 (discussed later) was traced on MIDAS for further demographic information (NHS number where necessary, postcode etc.) that would allow subsequent linkage to HES. Additionally, the matched NCP controls were also identified using MIDAS.

Inclusion and Exclusion criteria

ART: defined as "treatments or procedures that include in vitro handling of both human oocytes and sperm or embryos, for the purpose of reproduction" (52). This study included all children born after non-donor ART conducted in the UK between 1st April 1992 and 31st July 2009, in keeping with legislative changes that influenced the ability to link HFEA data to other health datasets (39, 41). It was estimated that only 0.2% of patients receiving ART during the study period retrospectively withdrew consent (53).

NCS**:** All naturally conceived (NC) children born to women with at least one child born following ART were included. This comprised of both full (both parents the same) and maternal half-siblings (same mother different father), but identification of paternal half-siblings was not possible.

NCP controls**:** The birth registration dataset and MIDAS were used to identify two matched [by month and year of birth, sex, and multiplicity/plurality] NCP controls per ART child (ART: control = 1:2).

For peer review only

All children conceived in the UK after ART but born outside of England, Wales & Scotland, those born after ART to women who permanently lived outside the UK but travelled to the UK for ART treatment, and those born in Northern Ireland were excluded as it would not be possible to link them to ONS birth records. Additionally, siblings born outside of the study period (as their conception status could not be verified) as well as those born outside of England, Wales & Scotland were also excluded. Cases that had withdrawn consent for their data to be used for research and children born after donor ART were excluded in compliance with HFEA legislation which prevents the viewing of identifiable data relating to these children by any third party.

Linkage

As described above, the HFEA Register contains very few identifiers for children born after ART, thus limiting linkage to other nationally held datasets. Therefore, this study initially linked HFEA records of children born to women known to have undergone ART to nationally held birth records to add identifiers and NHS numbers which would enable further linkage with a variety of other datasets (shown in Figures 1 & 2).

Linkage 1: HFEA-ONS

Step 1 [1 & 2 from Figure 1]: The HFEA first identified children born after ART and assigned them pseudo-anonymised unique record numbers (URN). Maternal unique record numbers (mURN; created from a previous study (53) [Supplementary Table S2] were added to the file, which was encrypted and sent securely to NHS Digital.

This file contained the following variables: date of birth (DOB) of child, BW of child, sex of child, forename of mother, surname of mother, previous name(s) of mother, multiplicity/plurality (e.g., singleton, twin), and mother's DOB.

Step 2 [3 & 4 from figure 1]: NHS Digital created file 2 [from a previous study (53); Supplementary Table S2] containing extracts of women who had undergone ART within the specified time period and securely transferred it to ONS. This file contained the NHS Number, DOB of possible mother, surname of possible mother, forename of possible mother, other name(s) of possible mother, and the HFEA mURN.

ONS then used these variables to link to birth records to create an extract of all children born to these mothers within the specified time frame. This extract (File 3) contained the mothers' details as supplied in File 2, surname of the child, forename of the child, DOB of the child, NHS number of the child (if available), BW of the child, and sex of the child. This file was then securely transferred back to NHS Digital.

For peer review only

Step 3 [5, 6, 7, 8 & 9 from figure 1]: NHS Digital then matched files 1 & 3 to determine children born to these mothers after ART and their NCS. This linkage was carried out using DOB of child, NHS number of child (if available), BW of child, sex of child, surname of child (where available on HFEA file 1), and forename of child (where available on HFEA file 1).

The ART and sibling cohorts were then traced using NHS Digital's national Medical Integrated Database and Administration System (MIDAS) to update demographic information (including NHS number, postcode) and current status (emigrations/ deaths etc.) and allow subsequent linkage to medical records.

Step 4 [10 &11 from figure 1]: NHS Digital used MIDAS to identify 2 controls for every cohort member, matched for month and year of birth, sex, and multiplicity/plurality (singleton/ multiple birth).

Step 5 [14 from figure 1]: NHS Digital produced updated demographic information on all ART children, siblings and controls (as obtained from MIDAS) and provided de-identified outputs plus non-identifiable deprivation scores to the research team at University College London (UCL).

Linkage 2: HFEA-ONS-HES

Step 6 [15 on figure 2]: NHS Digital then linked all cohorts produced by linkage 1 to HES using NHS number, postcode, and DOB, and shared with the research team at UCL.

Step 7 [16 on figure 2]: HFEA staff extracted relevant anonymised fertility data and this was shared with the research team at UCL who linked these two files (from steps 6 and 7) using the URNs.

Step 8 [17 on figure 2]: For the purpose of this second linkage, UCL researchers excluded records of children born before 1st April 1997 to coincide with the start of HES monitoring as well as triplets and higher order births from all groups (along with associated ART and NC births) to produce the final cohorts for health outcome analysis.

Step 9 [18 on figure 2]: NHS Digital produced file 4 (shown in Figure 1) for the ART and NC cohorts and these are now securely stored at HFEA and NHS Digital, respectively.

The entire linkage process was completed by NHS-Digital and none of the researchers had access to any identifiable participant data. The details of the ART and NC groups were encrypted and are currently held securely at HFEA and NHS-Digital, respectively.

For peer review only

Cohorts produced

Linkage 1: HFEA-ONS cohort

a) *ART*: This group consisted of all children born after non-donor ART conducted in the UK between 1st April 1992 and 31st July 2009.

b) *NCS:* This included all NC children born to women who had at least one child born following ART. This included full and maternal half-siblings but not paternal half-siblings.

c) *NCP:* This consisted of two matched (by month and year of birth, sex, and multiplicity/plurality) NCP controls for each ART child.

Linkage 2: HFEA-ONS-HES sub-cohort

a) *ART with HES outcomes (HES-ART):* This included all children born after non-donor ART conducted in the UK between 1st April 1997 and 31st July 2009 linked to their HES outcomes.

b) *NCS with HES outcomes (HES-NCS):* This included the NCS of all non-donor ART children born in the UK between 1st April 1997 and 31st July 2009 linked to their HES outcomes.

c) *NCP control with HES outcomes (HES-NCP):* This included two matched NCP controls for each non-donor ART child born in the UK between 1st April 1997 & 31st July 2009 linked to their HES outcomes.

Data Cleaning

Data cleaning included deletion of triplets and higher order births along with their NCS and matched NCP controls; deletion of duplicates; and reformatting, labelling and creation of new variables. Triplets and higher order births were excluded from analysis as they are known to be associated with adverse perinatal outcomes such as higher infant mortality, birth defects, premature birth, and low BW (54, 55). Initial data scoping also revealed several discrepancies in the data, a common limitation of large data linkage studies utilising multiple data sources, and pragmatic rules were employed to derive a consensus. Additional data cleaning carried out on the sub-cohort produced via linkage 2 included creation of an additional group consisting of ART children who had NCS (HES-sART) for the purposes of analysis. Order of pregnancy was calculated by sorting the child DOBs within each family in ascending order (Supplementary tables S3), and the type of ART and cause of infertility variables were regrouped appropriately.

For peer review only

Exemplar analysis

An exemplar analysis examining the effect of fresh and frozen embryo transfer (each compared to NC) on singleton BW was carried out using the HFEA-ONS cohort and HFEA-ONS-HES sub-cohort (inclusion criteria discussed previously). Details of BW are provided by the hospital where the birth took place or by the midwife/doctor in attendance at the birth, and is then passed to ONS as a consequence of the NHS birth notification being linked to the corresponding birth registration by the registrar. Multiple regression analysis was used to compare BW between children born after ART and NCP controls. The models were adjusted for maternal age at delivery and sex for the ART vs NCP analysis, and for maternal age at delivery, sex, IMD at earliest appointment, and ethnicity for the HES-ART vs HES-NCP analysis.

A family-matched model was used to compare BW between ART children and their NCS, including a family covariate to allow for within family correlations. The models were adjusted for maternal age at delivery, sex, and order of pregnancy. IMD and ethnicity were excluded from this analysis as the underlying effects they represent would have remained constant within families. The models were parameterised to directly estimate the differences between ART (fresh and frozen) and NC children. The fresh vs frozen difference was estimated from the model by construction of the appropriate contrast. All analyses were carried out using Stata v16.0.

This paper was developed in accordance with the STROBE reporting guidelines (56).

Patient and Public Involvement

No patients involved. Due to the very personal nature of the treatments involved, it was not appropriate to contact the families directly thus preventing us from involving patients or the public in the design, conduct, reporting, or dissemination plans of our research.

RESULTS

Final HFEA-ONS cohort

The HFEA identified 110596 children that met our inclusion criteria, of which 97660 (Figure 1) were deemed suitable for transfer to NHS Digital and subsequent linkage to ONS birth records. The linkage success rate was 77% (75348 cases out of 97660) due to the poor quality of data received from the HFEA.

A feasibility pilot study testing the validity of the linkage carried out in this study showed that the false positivity rates were very low (<0.0002%, unpublished data). Matching was done using SQL®

For peer review only

using exact matching (design to be inclusive of all potential matches) followed by probabilistic matching using Jaro Winkler® software. A clash was defined as a complete incompatibility of information between databases for a particular variable, with no clashes deemed as being a match. The HFEA were asked for further information in case of one clash, and more than one clash was considered to be a failed match. In the current study, the main linkage was carried out using a very high threshold for matching, and the weak/inaccurate identifiers in the HFEA register often resulted in matches that failed to pass this threshold. The majority of unlinked records could not be found due to clashes in parental identifiers and child date of birth.

Moreover, as the main linkage carried out in this study was bespoke in nature, particularly with regard to identification of the naturally conceived siblings, and had not been carried out before, standardized quality assurance measures could not be applied and instead extensive manual checking of the linkage was implemented. However, due to the mandatory nature of reporting all ART cycles carried out in the UK to the HFEA, it is unlikely that the unlinked records differed significantly from those that were linked, thus minimizing the risk of selection bias.

Tables 1 & 2 shows the descriptive characteristics of the final HFEA-ONS cohort generated after completion of Linkage 1 (Figure 1) and data cleaning. This cohort included 75348 children born after ART between 1992 and 2009 and 164823 matched NCP controls. Of the children born after ART, 15875 (sART) had 14763 NCS and the majority of families had had an ART pregnancy first followed by natural conception (Supplementary table S3).

The majority of the final cohort were ethnically white and singleton births, and a greater proportion of the ART and NCS groups were from a higher socioeconomic class. The majority of women who underwent ART were aged between 30-39 years at the time of delivery, while those with NC births tended to be slightly younger (25-39 years).

For peer review only

Table 1: Demographic characteristics of ART children, naturally conceived siblings, and matched naturally conceived population controls born between 1st April 1992 & 31st July 2009.

	HFEA-ONS cohort			
	**ART**	**Control (NCP)**	**ART with siblings (sART)**	**Siblings (NCS)**
**Infants**	75348	164823	15875	14763
**Mean BW (S.D)**				
Singleton	3282.0 (620.3)	3353.83 (578.5)	3311.12 (605.1)	3430.05 (581.9)
Multiple	2378.3 (593.4)	2340.28 (626.1)	2405.10 (608.1)	2292.03 (780.5)
**Sex**				
Female	36994 (49.1 %)	80859 (49.1 %)	7598 (47.9 %)	7271 (49.3 %)
Male	38354 (50.9 %)	83964 (50.9 %)	8277 (52.1 %)	7492 (50.8 %)
**Multiplicity/plurality**				
Singleton	44488 (59.1 %)	99554 (60.4 %)	10592 (66.7 %)	14055 (95.2 %)
Twin	30860 (40.9 %)	65269 (39.6 %)	5283 (33.3 %)	708 (4.8 %)
**Maternal age group**				
<25	731 (0.1 %)	33156 (20.1 %)	256 (1.6 %)	388 (2.6 %)
25-29	5315 (7.1 %)	30231 (18.3 %)	1326 (8.4 %)	1032 (6.1 %)
30-34	23342 (30.1 %)	46050 (27.9 %)	5466 (34.4 %)	3855 (26.1 %)
35-39	29187 (38.7 %)	30345 (18.4 %)	5872 (36.1 %)	5711 (38.7 %)
40-44	8705 (11.6 %)	7812 (4.7 %)	1315 (8.3 %)	2157 (14.6 %)
>=45	84 (0.1 %)	587 (0.4 %)	23 (0.1 %)	53 (0.4 %)
Missing	7984 (10.6 %)	16642 (10.1 %)	1617 (10.2 %)	1567 (10.6 %)
**Birth year**				
1992	57 (0.1 %)	131 (0.1 %)	12 (0.08%)	23 (0.2 %)
1993	1595 (2.1 %)	3187 (1.9 %)	299 (1.8%)	337 (2.3 %)
1994	2021 (2.7 %)	4029 (2.4 %)	425 (2.7 %)	389 (2.6 %)
1995	2439 (3.2 %)	4911 (2.1 %)	518 (3.3 %)	526 (3.6 %)
1996	2977 (3.1 %)	5989 (3.6 %)	641 (4.0 %)	577 (3.9 %)
1997	3574 (4.7 %)	7172 (4.4 %)	776 (4.9 %)	679 (4.6 %)
1998	3826 (5.1 %)	7670 (4.7 %)	861 (5.4 %)	800 (5.4 %)
1999	4253 (5.6 %)	8531 (5.2 %)	1023 (6.4 %)	804 (5.5 %)
2000	4504 (5.1 %)	9106 (5.5 %)	1119 (7.1 %)	961 (6.5 %)
2001	4754 (6.3 %)	9677 (5.9 %)	1163 (7.3 %)	991 (6.7 %)
2002	5149 (6.8 %)	10464 (6.4 %)	1339 (8.4 %)	999 (6.8 %)
2003	5531 (7.3 %)	11373 (6.9 %)	1401 (8.8 %)	1064 (7.2 %)
2004	5671 (7.5 %)	11835 (7.2 %)	1379 (8.7 %)	1098 (7.4 %)
2005	5768 (7.7 %)	12310 (7.5 %)	1372 (8.6 %)	1109 (7.5 %)
2006	6331 (8.4 %)	14248 (8.6 %)	1273 (8.0 %)	1116 (7.6 %)
2007	6410 (8.5 %)	15976 (9.7 %)	1126 (7.1 %)	1209 (8.9 %)
2008	6395 (8.5 %)	17222 (10.5 %)	719 (4.5 %)	1271 (8.6 %)
2009	4092 (5.4 %)	10992 (6.7 %)	429 (2.7 %)	810 (5.5 %)

HFEA- Human Fertilization & Embryology Authority; ONS- Office for National Statistics; NCP: naturally conceived population controls; NCS- naturally conceived siblings; sART- ART with siblings; BW - birthweight; S.D - standard deviation

For peer review only

Table 2: Fertility characteristics of ART children by cohort

	HFEA-ONS cohort		HFEA-ONS-HES sub-cohort	
	ART	sART (ART with siblings)	HES-ART	HES-sART (ART with siblings)
**Multiplicity/plurality**				
Singletons	44489 (59.0 %)	10592 (66.7 %)	37891 (59.4 %)	8383 (67.1 %)
Twins	30859 (40.1 %)	5283 (33.3 %)	25986 (40.6 %)	3946 (32.0 %)
**Paternal age at childbirth**				
<25	406 (0.5 %)	122 (0.8 %)	324 (0.5 %)	87 (0.7 %)
25-29	4878 (6.5 %)	1206 (7.6 %)	3911 (6.1 %)	938 (7.6 %)
30-34	21526 (28.6 %)	5304 (33.4 %)	17706 (27.7 %)	4116 (33.4 %)
35-39	27678 (36.7 %)	6007 (37.8 %)	23626 (36.1 %)	4692 (38.1 %)
40-44	13458 (17.9 %)	2306 (14.5 %)	11715 (18.3 %)	1778 (14.4 %)
45-49	4551 (6.0 %)	604 (3.8 %)	4010 (6.3 %)	468 (3.8 %)
50-54	1636 (2.2 %)	190 (1.2 %)	1440 (2.3 %)	146 (1.2 %)
>=55	848 (1.1 %)	82 (0.5 %)	787 (1.2 %)	65 (0.5 %)
Missing	367 (0.5 %)	54 (0.4 %)	348 (0.6 %)	39 (0.3 %)
**Type of ART**				
ICSI	26629 (38.6 %)	4409 (30.1 %)	26210 (44.6 %)	3995 (34.1 %)
IVF	42096 (61.0 %)	10170 (69.4 %)	32282 (54.1 %)	7364 (64.4 %
IVF:ICSI	248 (0.4 %)	73 (0.5 %)	247 (0.4 %)	72 (0.6 %)
**Cause of infertility**				
Male factor	29203 (38.8 %)	5675 (35.8 %)	24876 (38.1 %)	4198 (34.1 %)
Endometriosis	2225 (2.1 %)	495 (3.1 %)	1962 (3.1 %)	401 (3.3 %)
Ovulatory	8581 (11.4 %)	2198 (13.9 %)	7713 (12.1 %)	1854 (15.1 %)
Tubal	19915 (26.4 %)	3450 (21.7 %)	15041 (23.6 %)	2378 (19.3 %)
Unknown cause	15387 (20.4 %)	4048 (25.5 %)	14252 (22.3 %)	3491 (28.3 %)
**Fresh/Frozen transfer**				
Fresh	66534 (88.3 %)	14079 (88.7 %)	56230 (88.0 %)	10907 (88.5 %)
Frozen	8739 (11.6 %)	1782 (11.2 %)	7595 (11.9 %)	1414 (11.7%)
Missing	75 (0.1 %)	14 (0.1 %)	52 (0.1 %)	8 (0.1 %)
**Previous live births**				
0	67235 (89.2 %)	14878 (93.7 %)	56250 (88.1 %)	11498 (93.3 %)
1	7829 (10.4 %)	958 (6.1 %)	7348 (11.5 %)	795 (6.5 %)
2	273 (0.4 %)	35 (0.2 %)	268 (0.4 %)	32 (0.3 %)
3	9 (0.0 %)	2 (0.0 %)	9 (0.1 %)	2 (0.0 %)
Missing	2 (0.0 %)	2 (0.0 %)	2 (0.0 %)	2 (0.0 %)
**Parity of mother (previous pregnancies as recorded on the register)**				
0	64502 (85.6 %)	14270 (89.9 %)	53987 (84.5 %)	11029 (89.5 %)
1	9798 (13.0 %)	1450 (9.2 %)	8901 (13.9 %)	1167 (9.5 %)
2	949 (1.3 %)	140 (0.9 %)	894 (1.4 %)	119 (0.1 %)
3	83 (0.1 %)	9 (0.1 %)	79 (0.1 %)	8 (0.1 %)
4	14 (0.0 %)	4 (0.0 %)	14 (0.0 %)	4 (0.0 %)
Null	2 (0.0 %)	2 (0.0 %)	2 (0.0 %)	2 (0.0 %)
**Infertility duration in years**				
Mean (SD)	4.78 (2.9)	4.37 (2.6)	4.62 (2.8)	4.31 (2.6)
Missing	17451 (23.2 %)	4951 (31.2 %)	17898 (24.9 %)	5364 (27.1 %)

HFEA- Human Fertilization & Embryology Authority; ONS- Office for National Statistics; HES- Hospital Episode Statistics; sART- ART with siblings; ICSI - intracytoplasmic sperm injection; IVF - in vitro fertilisation

For peer review only

Table 3: Demographic characteristics of ART children, naturally conceived siblings, and matched naturally conceived population controls born between 1st April 1997 and 31st July 2009.

	HFEA-ONS-HES sub-cohort			
	**HES-ART**	**Control (HES-NCP)**	**ART with siblings (HES-sART)**	**Siblings (HES-NCS)**
**Infants**	63877	127544	12329	11343
**Follow-up period in days**				
**Median (IQR)**	4429 (2181)	4409 (2141)	4635 (1846)	4307 (2029)
**Mean BW (S.D)**				
Singleton	3166.95 (742.3)	3271.67 (648.5)	3222.25 (699.3)	3346.54 (712.0)
Multiple	2172.27 (715.4)	2155.77 (683.9)	2201.58 (724.9)	2301.17 (677.8)
**Sex**				
Female	31435 (49.2 %)	62785 (49.2 %)	5907 (47.9 %)	5573 (49.1 %)
Male	32442 (50.8 %)	64759 (50.8 %)	6422 (52.1 %)	5770 (50.9 %)
**Multiplicity/plurality**				
Singleton	37890 (59.4 %)	75642 (59.3 %)	8383 (68.0 %)	10815 (95.8 %)
Twin	25987 (40.6 %)	51902 (40.7 %)	3946 (31.1 %)	528 (4.2 %)
**IMD decile at earliest**				
1 (most deprived)	2045 (3.2 %)	12696 (9.9 %)	349 (2.8 %)	299 (2.6 %)
2	2650 (4.2 %)	11104 (8.7 %)	458 (3.7 %)	396 (3.5 %)
3	3311 (5.2 %)	10057 (7.8 %)	539 (4.4 %)	463 (4.1 %)
4	3926 (6.1 %)	9550 (7.5 %)	678 (5.4 %)	588 (5.2 %)
5	4666 (7.2 %)	9161 (7.5 %)	862 (6.1 %)	746 (6.6 %)
6	5350 (8.4 %)	8872 (6.5 %)	1084 (8.8 %)	921 (8.1 %)
7	6142 (9.6 %)	8842 (6.9 %)	1205 (9.8 %)	1044 (9.2 %)
8	6675 (10.5 %)	8925 (7.0 %)	1299 (10.5 %)	1161 (10.3 %)
9	7729 (12.1 %)	9083 (7.1 %)	1557 (12.6 %)	1386 (12.2 %)
10 (least deprived)	7710 (12.1 %)	8299 (6.5 %)	1720 (14.0 %)	1499 (13.3 %)
Missing	13673 (21.4 %)	30955 (24.3 %)	2580 (20.9 %)	2840 (25.0 %)
**Birth year**				
1997	2597 (4.1 %)	5160 (4.1 %)	518 (4.2 %)	242 (2.1 %)
1998	3708 (5.8 %)	7389 (5.9 %)	743 (6.0 %)	419 (3.7 %)
1999	4083 (6.4 %)	8126 (6.5 %)	852 (6.9 %)	560 (4.9 %)
2000	4310 (6.8 %)	8633 (6.9 %)	925 (7.5 %)	781 (6.9 %)
2001	4559 (7.1 %)	9160 (7.3 %)	972 (7.9 %)	879 (7.78 %)
2002	4980 (7.8 %)	9933 (7.9 %)	1171 (9.5 %)	937 (8.3 %)
2003	5379 (8.4 %)	10788 (8.5 %)	1253 (10.2 %)	1012 (8.9 %)
2004	5561 (8.7 %)	11082 (8.7 %)	1271 (10.3 %)	1067 (9.4 %)

For peer review only

Final HFEA-ONS-HES sub-cohort

All births before 1st April 1997 were excluded for the purpose of linkage to HES, resulting in 63877, 11343 and 127544 children in the HES-ART, HES-NCS, and HES-NCP groups, respectively. Linkage 2 (Figure 2) had a success rate of 84.7%, and the descriptive characteristics of the sub-cohort produced have been shown in Tables 2 & 3

2005	5662 (8.9 %)	11326 (8.8 %)	1271 (10.3 %)	1078 (9.5 %)
2006	6275 (9.8 %)	12513 (9.7 %)	1217 (9.7 %)	1100 (9.7 %)
2007	6342 (9.9 %)	12701 (9.8 %)	1058 (8.6 %)	1199 (10.6 %)
2008	6347 (9.9 %)	12718 (9.8 %)	670 (5.4 %)	1260 (11.1 %)
2009	4074 (6.4 %)	8015 (6.2 %)	408 (3.3 %)	809 (7.1 %)
**Ethnicity**				
White	61921 (96.9 %)	122050 (95.7 %)	11983 (97.2 %)	11084 (97.7 %)
Asian/Asian British	959 (1.5 %)	2496 (1.1 %)	197 (1.6 %)	153 (1.4 %)
Chinese	35 (0.1 %)	89 (0.1 %)	8 (0.1 %)	1 (0.0 %)
Black/African/Caribbean/Black	433 (0.7 %)	5268 (4.0 %)	61 (0.5 %)	39 (0.3 %)
Mixed/Multiple ethnic groups	318 (0.5 %)	721 (0.6 %)	38 (0.3 %)	27 (0.2 %)
Other ethnic group	211 (0.3 %)	498 (0.4 %)	42 (0.3 %)	39 (0.3 %)
**Maternal age at delivery**				
<=25	710 (1.1 %)	27783 (21.9 %)	241 (1.1 %)	317 (2.9 %)
25-29	5085 (7.1 %)	25115 (19.7 %)	1209 (9.9 %)	887 (7.8 %)
30-34	21994 (34.4 %)	38896 (30.5 %)	4797 (38.9 %)	3309 (29.1 %)
35-39	27682 (43.4 %)	25907 (20.3 %)	4998 (40.6 %)	4954 (43.7 %)
40-44	8164 (12.1 %)	6419 (5.2 %)	1057 (8.6 %)	1830 (16.2 %)
>=45	217 (0.3 %)	772 (0.6 %)	20 (0.2 %)	45 (0.4 %)
Missing	25 (0.0 %)	2652 (2.0 %)	7 (0.0 %)	1 (0.0 %)

HFEA- Human Fertilization & Embryology Authority; ONS- Office for National Statistics; HES- Hospital Episode Statistics; sART- ART with siblings; ICSI - intracytoplasmic sperm injection; IVF - in vitro fertilisation

Exemplar analysis: Singleton BW

The exemplar analysis included all singleton births in the HFEA-ONS cohort and HFEA-ONS-HES sub-cohort.

HFEA-ONS: Comparing ART to NCP showed that children born after fresh embryo transfers were lighter (BW difference: -131g; 95% CI: -140, -123) and those born after frozen embryo transfers were heavier (BW difference: 35g; 95% CI: 19, 52) than the NC controls (Table 4; Supplementary table S4). Moreover, children born after frozen embryo transfers were significantly heavier than those born after fresh embryo transfers (BW difference: -167g; 95% CI: -183, -150).
Family-matched analysis showed that children born after fresh embryo transfers were lighter (BW difference: -54g; 95% CI: -72, -36) and those born after frozen embryo transfers (BW difference: 138g; 95% CI: 101, 175) were heavier than the NCS group (Table 4; Supplementary table S5). Moreover, children born after frozen embryo transfers were significantly heavier than those born after fresh embryo transfers (BW difference =-193g; 95% CI: -232, -154).

HFEA-ONS-HES: Comparing HES-ART to HES-NCP showed that children born after fresh embryo transfers were lighter (BW difference: -152g; 95% CI: -162, -142) and those born after frozen embryo

transfers were similar in weight (BW difference: 3g; 95% CI: -17, 22) to the NC controls (Table 4; Supplementary table S6). Moreover, children born after frozen embryo transfers were significantly heavier than those born after fresh embryo transfers (BW difference:-155g; 95% CI: -175, -135). Family-matched analysis comparing showed that children born after fresh embryo transfers were lighter (BW difference: -57g; 95% CI: -75, -38) and those born after frozen embryo transfers were heavier (BW difference: 152g; 95% CI: 113, 190) than the HES-NCS group (Table 4; Supplementary table S7). Moreover, children born after frozen embryo transfers were significantly heavier than those born after fresh embryo transfers (BW difference: -209g; 95% CI: -249, -168).

Table 4: Statistical analysis of BW

	**HFEA-ONS cohort** *BW coefficient (95%CI)*		**HFEA-ONS-HES sub-cohort** *BW coefficient (95%CI)*	
	**ART (N=75348) – NCP (N=164823)** * adjusted for maternal age at delivery and sex	**sART (N=15875) NCS (N=14763)** *adjusted for maternal age at delivery, sex, and order of pregnancy.	**HES-ART (N=63877) HES-NCP (N=127544)** *adjusted for maternal age at delivery, sex, IMD at earliest appointment, and ethnicity.	**HES-sART (N=12329) – HES-NCS (N=11343)** *adjusted for maternal age at delivery, sex, and order of pregnancy.
Fresh vs NC (g)	-131 (-140, -123)	-54 (-72, -36)	-152 (-162, -142)	-57 (-75, -38)
Frozen vs NC (g)	35 (19, 52)	138 (101, 175)	3 (-17, 22)	152 (113, 190)
Fresh vs Frozen (g)	-167 (-183, -150)	-193 (-232, -154)	-155 (-175, -135)	-209 (-249, -168)

HFEA- Human Fertilization & Embryology Authority; ONS- Office for National Statistics; HES- Hospital Episode Statistics; sART- ART with siblings; NCP- Naturally conceived population control; NCS- Naturally conceived siblings; NC- naturally conceived; BW – birthweight; CI- confidence interval.

DISCUSSION

This study aimed to substantially enhance the research value of the HFEA register by utilising electronic record linkage methodology to establish a cohort of ART children born in the UK between

For peer review only - http://bmjopen.bmj.com/site/about/guidelines.xhtml

1992 and 2009, their NCS and matched NCP controls. Additionally, a sub-cohort consisting of those born between 1997 and 2009 was also linked to the Hospital Episode Statistics database to allow examination of postnatal health outcomes. The final cohort consisted of 75348 children born after non-donor ART carried out in the UK between 1st April 1992 and 31st July 2009, 14763 NCS and 164823 matched NCP controls. Of these, 63877 ART, 11343 NCS, and 127544 matched NCP controls were linked to hospital data up to 2015, thus providing a valuable resource for comprehensive, non-invasive, continued immediate and longer term health monitoring of this population as they grow up.

An exemplar analysis comparing BW between children born after ART, their NCS and matched NCP controls was also carried out to demonstrate the validity of this cohort. The results of this analysis confirmed the findings of our previous linkage study on a subset of the entire 1992-2009 cohort (39), that children born after fresh embryo transfer tended to be lighter and those born from frozen transfer were heavier compared to their NCS and matched NCP controls. These findings are broadly in agreement with numerous large register-based studies and meta-analyses, but add valuable confirmation of the magnitude of BW differences in a large UK population (15, 57-65). Castillo et al (66) reported a similar magnitude of difference to that seen in sibling pairs in a more recent UK cohort, suggesting that the difference in BW between the fresh and frozen embryo transfer groups may not be explained entirely by maternal characteristics and the underlying cause of infertility as these were likely to have remained relatively stable between siblings. Notably, the BW difference observed between frozen transfer and NC babies is much larger in the sibling pair analysis than in the non-sibling comparison, and larger than observed by Hann et al (39). This merits further investigation, especially as increased rates of macrosomia following frozen transfer are an ongoing concern (34, 62). Other possible explanations include differences in the maternal uterine environment caused by either hormonal stimulation (fresh cycles) or hormonal preparation of the uterus (frozen cycles) (67). Although lack of appropriate data on the HFEA register prevented exploration of these effects in the current analysis, a recent UK cohort study by Castillo et al using more detailed clinic data reported no association between type of FET and birthweight (66). Molecular changes in the early embryo during freezing and thawing might also result in changes in the developmental process leading to altered BW and long term health (33, 68-71).

In the current study, the difference in BW between children born after fresh embryo transfer and those born after natural conception was seen to attenuate markedly from -131g in the population to -54g in the within-sibling analysis. By contrast, the difference in BW between children born after frozen embryo transfer and those born after natural conception was seen to markedly increase from 35g in the population to 138g in the within sibling analysis in the same dataset. This suggests

For peer review only

that the findings of the within-family analysis must be interpreted with caution, with potential explanatory factors for these differences including subfertility and familial masking confounders in relation to the increase of the association of frozen transfer vs natural conception. Unavailability of birth order data for the population controls prevented us from adjusting for it in the ART vs NCP comparison, and this could plausibly act as a strong confounder given its association with mean BW is stronger than any differences due to ART in most studies. This notable difference between the population and within-family analyses requires further exploration.

Repeating the exemplar analysis using the cohort and sub-cohort produced by the linkages described above not only increased confidence in the findings but also allowed us to control for potential confounding factors such as maternal age, birth order, socioeconomic status and ethnicity which are known to affect BW (72).

The cohort described here will allow more effective use of HFEA data for health monitoring, and will be of value to researchers from a variety of professional backgrounds. The linked dataset will be returned to the HFEA and access will be controlled by them, although specific ethical approval will be required along with that of the Confidentiality Advisory Group (CAG) of the Health Research Authority (HRA).

In order to provide reliable risk estimates of relatively rare conditions in a specific group of children born after ART, large, well-designed cohort studies, preferably including families, are necessary. However, as it may not always be appropriate to contact these families directly due to the very personal nature of the treatments involved, access to national-level electronically linked data on cohorts such as the current one provides a cost-effective, non-intrusive, and comprehensive way to answer sophisticated research questions in a wide range of areas.

The main strength of this study lies in the meticulous linkage of robust, routinely collected administrative health data to yield a large cohort that is nationally unique and complements other similar linked cohorts, such as the Nordic linked cohorts (19), thus increasing the generalisability, accuracy, and precision of results from subsequent analyses. The research value and quality and consistency of the HFEA cohort 1992-2009 data has been considerably enhanced through linkage to the ONS birth records and HES databases and removal of artefacts and duplicates. Linkage to the hospital admissions and outpatient database provides long-term mortality and morbidity outcome data on offspring, and also represents a high-quality cross-sectoral evidence base that can be used for longitudinal research, policy planning and strategic development.

However, there are also several challenges associated with electronic data linkage studies in general, and Harron et al. (73) summarised these into three groups, namely those pertaining to the (i) the data linkage environment and privacy preservation; (ii) the linkage process itself, which includes data

For peer review only

preparation linkage methods; and (iii) the linkage quality and potential bias in linked data. In the context of the current study, limitations in the linkage process itself included those associated with the method of definition of NC siblings used. The identification of sibling controls would be very sensitive to any errors in linkage, and missed second ART babies would appear as conventional siblings. Parents who had NC children were likely to have been less severely sub-fertile than parents who did not, and ART children born to the truly infertile would not have conventional siblings. Therefore, the NCS group in this study would have parents with borderline fertility problems or those who developed secondary infertility after the birth of the NC child. For this reason, extensive quality assurance procedures were carried out on the linkage process. With regard to the linkage quality, as this was a bespoke linkage that has never been carried out before, there were no standardized quality assurance measures in place; however, the linkage was extensively checked manually by the team at NHS Digital prior to dissemination for analysis.

The often weak/inaccurate identifier data on the HFEA register and the high threshold for matching used in the current study meant that approximately 23% of children were lost during the linkage process. This is a limitation as almost 100% coverage can be achieved in settings with availability of unique identifying numbers for both mother and child. However, although unavailable for the study period explored here, the HFEA now record both the mother and child's NHS number, suggesting that this loss to follow-up may be avoided in future studies (74). As HES monitoring data is only available from 1997, children born to women who underwent ART prior to this could not be linked to any hospital records, thus limiting our ability to examine health outcomes in those born before 1997 and exploring the effects of changes in ART techniques on health over that period. However, comparison of maternal age between the unlinked and linked records showed no substantive differences (Supplementary table S8). Unfortunately, unavailability of data prevented us from carrying out further examination and comparison of the educational levels and socioeconomic profile of unlinked records. Nevertheless, linkage of cases born before 1997 to their birth records is still valuable as it may allow investigation of outcomes available on the birth register in the future. Moreover, although these data have been used extensively for research purposes, there have been long-standing concerns regarding the quality, completeness and coverage of HES records within health services and the academic community (75).

The BW analysis provides an example use of these cohorts and the strengths and limitations of sibling comparisons. However, it cannot be considered as an exhaustive analysis as gestational data was not available for NC children and there is limited covariate data in the present dataset. This is

For peer review only

mainly because gestation is not recorded on the birth registration dataset and is of poor quality on the maternal arm of a child's HES record (>50% missing). As a result, although the quality of such data for the ART group (accessed from the HFEA register) was good, it was not available for the NCS and NCP control groups. Further ART treatment data is available on the HFEA register which was not included in the current datasets, and these could be extracted for future studies. In particular the developmental stage of the embryo at transfer and number of embryos transferred are important determinants of treatment success rate (i.e. the probability of a child appearing on the HFEA birth register) and also gestation and BW (37, 39, 66). Unfortunately, there was no way to systematically identify and account for children born after ART who emigrated or children born to mothers who lived in the England & Wales but travelled abroad for ART treatment, although European estimates of cross-border reproductive care activity suggests that the number of children to whom either of these applies is be small (76). Due to lack of maternal data, it was not feasible to identify and exclude NCP controls that were part of a triplet or higher order birth. However, the number of such individuals is estimated to be small as the incidence of naturally occurring triplets is in the range of 1:5000-1:9000 (44).

Lastly, an important limitation of the current cohort is that it finishes in 2009. Given that ART technologies have advanced rapidly in the last 10 years, there is an urgent need to continue to prospectively monitor the next cohort of children from 2010 onwards. Two recent UK studies (57, 66) have suggested that BWs from both fresh and frozen transfers are increasing with time and may be different in the post 2010 cohort, suggesting that there may potentially be differences (of unknown origin) in the health outcomes of babies born more recently.

CONCLUSION

The current study carried out a bespoke record linkage that generated a new child cohort for use in exploring the relationship between conception via ART and short- and long-term health outcomes in offspring and enabling future linkage studies to other developmental and health data sources.

FOOTNOTE

Contributors: In line with the ICMJE authorship guidelines, authors AGS, SAR, DRB, SMN, BL, and DAL made substantial contributions to the conception or design of the work. Authors AGS and MP made substantial contributions to the acquisition of data. Author MP undertook data preparation and provided statistical analysis, with SAR and JG providing statistical oversight. Authors MP, SAR, JG, DRB, SMN, DAL, BL, and AGS made substantial contributions to the interpretation of data. All

For peer review only

authors contributed to the drafting of the manuscript, and/or the revising of the manuscript. All authors have given final approval of the version to be published, and agree to its accuracy.

Funding: This work was supported by the Medical Research Council [Grant number MR/L020335/1].

Competing interest: None declared.

Ethical considerations: Ethical approval and Section 251 support were obtained from the NHS Research Ethics Committee and Confidentiality Advisory Group, respectively. Additional data access permissions were sought from the HFEA Register Research Panel, ONS Micro-Data Release board, and the NHS Digital Medical Register. All researchers with data access underwent NHS Digital Data Security Awareness and ONS Safe Researcher accreditation.

Provenance and peer review: Not commissioned; externally peer reviewed.

Data sharing statement: De-identified linked cohort data can be accessed from the Human Fertilisation and Embryology Authority and NHS Digital where it will be held with restricted access. Specific ethical approval from the Research Ethics Committee (REC) and the Confidentiality Advisory Group (CAG) of the Health Research Authority (HRA) will be required for access.

For peer review only

REFERENCES

(1) Lancaster P, de Mouzon J. Global Committee Reproductive ART Surveillance: Monitoring Technologies Assisted The (ICMART) International. *Assisted Reproductive Technology Surveillance*. 2019:101.
2) Barker DJ. Fetal programming of coronary heart disease. *Trends in Endocrinology & Metabolism*. 2002;13(9):364-8.
(3) Lane M, Robker RL, Robertson SA. Parenting from before conception. *Science*. 2014;345(6198):756-60.
(4) Fleming TP, Watkins AJ, Velazquez MA, Mathers JC, Prentice AM, Stephenson J, et al. Origins of lifetime health around the time of conception: causes and consequences. *Lancet*. 2018;391(10132):1842-52.
(5) Fisher-Jeffes L, Banerjee I, Sutcliffe A. Parents' concerns regarding their ART children. *Reproduction*. 2006;131(2):389-94.
(6) Källén B, Finnström O, Nygren KG, Olausson PO. In vitro fertilization in Sweden: child morbidity including cancer risk. *Fertility and Sterility*. 2005;84(3):605-10.
(7) Schieve LA, Rasmussen SA, Buck GM, Schendel DE, Reynolds MA, Wright VC. Are children born after assisted reproductive technology at increased risk for adverse health outcomes? *Obstetrics Gynecology*. 2004;103(6):1154-63.
(8) Reddy UM, Wapner RJ, Rebar RW, Tasca RJ. Infertility, assisted reproductive technology, and adverse pregnancy outcomes: executive summary of a National Institute of Child Health and Human Development workshop. *Obstetrics Gynecology*. 2007;109(4):967-77.
(9) Pandey S, Shetty A, Hamilton M, Bhattacharya S, Maheshwari A. Obstetric and perinatal outcomes in singleton pregnancies resulting from IVF/ICSI: a systematic review and meta-analysis. *Human Reproduction Update*. 2012;18(5):485-503.
(10) Gnoth C, Godehardt E, Frank-Herrmann P, Friol K, Tigges J, Freundl G. Definition and prevalence of subfertility and infertility. *Human Reproduction*. 2005;20(5):1144-7.
(11) Ceelen M, van Weissenbruch MM, Prein J, Smit JJ, Vermeiden JP, Spreeuwenberg M, et al. Growth during infancy and early childhood in relation to blood pressure and body fat measures at age 8-18 years of IVF children and spontaneously conceived controls born to subfertile parents. *Human Reproduction*. 2009;24(11):2788-95.
(12) Ceelen M, van Weissenbruch MM, Vermeiden JP, van Leeuwen FE, Delemarre-van de Waal HA. Pubertal development in children and adolescents born after IVF and spontaneous conception. *Human Reproduction*. 2008;23(12):2791-8.
(13) Dumoulin JC, Land JA, Van Montfoort AP, Nelissen EC, Coonen E, Derhaag JG, et al. Effect of in vitro culture of human embryos on birthweight of newborns. *Human Reproduction*. 2010;25(3):605-12.
(14) Van Montfoort AP, Hanssen LL, de Sutter P, Viville S, Geraedts JP, de Boer P. Assisted reproduction treatment and epigenetic inheritance. *Human Reproduction Update*. 2012;18(2):171-97.
(15) Stormlund S, Sopa N, Zedeler A, Bogstad J, Prætorius L, Nielsen HS, et al. Freeze-all versus fresh blastocyst transfer strategy during in vitro fertilisation in women with regular menstrual cycles: multicentre randomised controlled trial. *British Medical Journal*. 2020;370:m2519.
(16) Sandin S, Nygren KG, Iliadou A, Hultman CM, Reichenberg A. Autism and Mental Retardation Among Offspring Born After In Vitro Fertilization. *Journal of the American Medical Association*. 2013;310(1):75-84.
(17) Bay B, Mortensen EL, Hvidtjørn D, Kesmodel US. Fertility treatment and risk of childhood and adolescent mental disorders: register based cohort study. *British Medical Journal*. 2013;347:f3978.
(18) Davies MJ, Moore VM, Willson KJ, Van Essen P, Priest K, Scott H, et al. Reproductive Technologies and the Risk of Birth Defects. *New England Journal of Medicine*. 2012;366(19):1803-13.

For peer review only

(19) Opdahl S, Henningsen A-KA, Bergh C, Gissler M, Romundstad LB, Petzold M, et al. Data Resource Profile: Committee of Nordic Assisted Reproductive Technology and Safety (CoNARTaS) cohort. *International Journal of Epidemiology*. 2019;49(2):365-6f.
(20) Henningsen AK, Romundstad LB, Gissler M, Nygren K-G, Lidegaard Ø, Skjaerven R, et al. Infant and maternal health monitoring using a combined Nordic database on ART and safety. *Acta Obstetricia et Gynecologica Scandinavica*. 2011;90(7):683-91.
(21) Wennberg AL, Opdahl S, Bergh C, Henningsen A-KA, Gissler M, Romundstad LB, et al. Effect of maternal age on maternal and neonatal outcomes after assisted reproductive technology. *Fertility and sterility*. 2016;106(5):1142-9. e14.
(22) Finnström O, Källén B, Lindam A, Nilsson E, Nygren KG, Olausson PO. Maternal and child outcome after in vitro fertilization--a review of 25 years of population-based data from Sweden. *Acta Obstetricia et Gynecologica Scandinavica*. 2011;90(5):494-500.
(23) Henningsen AK, Pinborg A, Lidegaard Ø, Vestergaard C, Forman JL, Andersen AN. Perinatal outcome of singleton siblings born after assisted reproductive technology and spontaneous conception: Danish national sibling-cohort study. *Fertility and Sterility*. 2011;95(3):959-63.
(24) Kalra SK, Ratcliffe SJ, Barnhart KT, Coutifaris C. Extended embryo culture and an increased risk of preterm delivery. *Obstetrics Gynecology*. 2012;120(1):69-75.
(25) Romundstad LB, Romundstad PR, Sunde A, von Düring V, Skjaerven R, Gunnell D, et al. Effects of technology or maternal factors on perinatal outcome after assisted fertilisation: a population-based cohort study. *Lancet*. 2008;372(9640):737-43.
(26) Lawlor DA, Tilling K, Davey Smith G. Triangulation in aetiological epidemiology. *International Journal of Epidemiology*. 2017;45(6):1866-86.
(27) Romundstad LB, Romundstad PR, Sunde A, von Düring V, Skjaerven R, Vatten LJ. Increased risk of placenta previa in pregnancies following IVF/ICSI; a comparison of ART and non-ART pregnancies in the same mother. *Human Reproduction*. 2006;21(9):2353-8.
(28) Human Fertilisation and Embryology Authority. *Fertility treatment 2014–2016 Trends and figures*. London: HFEA. 2018. URL: https://www.hfea.gov.uk/media/3188/hfea-fertility-trends-and-figures-2014-2016.pdf
(29) *Human Fertilisation and Embryology Act 1990* (UK). URL: https://www.legislation.gov.uk/ukpga/1990/37/contents
(30) *Human Fertilisation and Embryology Privacy Policy*. 2018. (UK) URL: https://www.hfea.gov.uk/privacy-policy-cookies-and-accessibility/privacy-policy/
(31) *Human Fertilisation & Embryology Authority* (2019). URL: https://www.hfea.gov.uk/.
(32) Templeton A, Morris JK, Parslow W. Factors that affect outcome of in-vitro fertilisation treatment. *Lancet*. 1996;348(9039):1402-6.
(33) Nelson SM, Lawlor DA. Predicting Live Birth, Preterm Delivery, and Low Birth Weight in Infants Born from In Vitro Fertilisation: A Prospective Study of 144,018 Treatment Cycles. *PLOS Medicine*. 2011;8(1):e1000386.
(34) Maheshwari A, Raja EA, Bhattacharya S. Obstetric and perinatal outcomes after either fresh or thawed frozen embryo transfer: an analysis of 112,432 singleton pregnancies recorded in the Human Fertilisation and Embryology Authority anonymized dataset. *Fertility and Sterility*. 2016;106(7):1703-8.
(35) Kamath MS, Antonisamy B, Selliah HY, Sunkara SK. Perinatal outcomes of singleton live births with and without vanishing twin following transfer of multiple embryos: analysis of 113 784 singleton live births. *Human Reproduction*. 2018;33(11):2018-22.
(36) Marconi N, Raja EA, Bhattacharya S, Maheshwari A. Perinatal outcomes in singleton live births after fresh blastocyst-stage embryo transfer: a retrospective analysis of 67 147 IVF/ICSI cycles. *Human Reproduction*. 2019;34(9):1716-25.
(37) Castillo CM, Johnstone ED, Horne G, Falconer DA, Troup SA, Cutting R, et al. Associations of IVF singleton birthweight and gestation with clinical treatment and laboratory factors: a multicentre cohort study. *Human Reproduction*. 2020;35(12):2860-70.

For peer review only

(38) Roberts S, McGowan L, Hirst W, Brison D, Vail A, Lieberman B. Towards single embryo transfer? Modelling clinical outcomes of potential treatment choices using multiple data sources: predictive models and patient perspectives. *Health Technology Assessment*. 2010;14(38):1-237.
(39) Hann M, Roberts SA, D'Souza SW, Clayton P, Macklon N, Brison DR. The growth of assisted reproductive treatment-conceived children from birth to 5 years: a national cohort study. BMC Medicine. 2018;16(1):224.
(40) *Human Fertilisation & Embryology Act 2008*. (UK). URL: https://www.legislation.gov.uk/ukpga/2008/22/contents
(41) Williams CL, Bunch KJ, Stiller CA, Murphy MFG, Botting BJ, Wallace WH, et al. Cancer Risk among Children Born after Assisted Conception. *New England Journal of Medicine*. 2013;369(19):1819-27.
(42) Human Fertilisation and Embryology Authority. *Fertility treatment 2018: trends and figures.* URL: https://www.hfea.gov.uk/about-us/publications/research-and-data/fertility-treatment-2018-trends-and figures/#:~:text=Birth%20rates%20from%20IVF%20have,HFEA%20was%20established%20in%201991.
(43) Human Fertilisation and Embryology Authority. *Fertility treatment 2018: trends and figures Quality and Methodology Report.* URL: https://www.hfea.gov.uk/about-us/publications/research-and-data/fertility-treatment-2018-trends-and-figures/fertility-treatment-2018-quality-and-methodology-report/
(44) Office for National Statistics. *User guide to birth statistics.*2019. URL: https://www.ons.gov.uk/peoplepopulationandcommunity/birthsdeathsandmarriages/livebirths/methodologies/userguidetobirthstatistics
(45) NHS Digital. *Hospital Episode Statistics*. URL: https://digital.nhs.uk/data-and-information/data-tools-and-services/data-services/hospital-episode-statistics.
(46) Great Britain. National Audit Office, Great Britain. Parliament. House of Commons. Healthcare across the UK: A comparison of the NHS in England, Scotland, Wales and Northern Ireland. The Stationery Office; 2012.
(47) The Private Healthcare Information Network. *Annual Report 2016-17.* URL: https://apicms.phin.org.uk/Content/Resource/158-PHIN_AR_2016-17.pdf
(48) Herbert A, Wijlaars L, Zylbersztejn A, Cromwell D, Hardelid P. Data Resource Profile: Hospital Episode Statistics Admitted Patient Care (HES APC). *International Journal of Epidemiology*. 2017;46(4):1093-i.
(49) Boyd A, Cornish R, Johnson L, Simmonds S, Syddall H, Westbury L, Cooper C, Macleod J. *Understanding Hospital Episode Statistics (HES)*. London, UK: CLOSER. 2017.
(50) Herbert A, Wijlaars L, Zylbersztejn A, Cromwell D, Hardelid P. Data Resource Profile: Hospital Episode Statistics Admitted Patient Care (HES APC). *International Journal of Epidemiology*. 2017;46(4):1093-i.
(51) Smith T, Noble M, Noble S, Wright G, McLennan D, Plunkett E. The English indices of deprivation 2015. London: Department for Communities and Local Government. 2015 Sep.
(52) Zegers-Hochschild F, Adamson GD, Dyer S, Racowsky C, de Mouzon J, Sokol R, et al. The International Glossary on Infertility and Fertility Care, 2017. *Fertility and Sterility*. 2017;108(3):393-406.
(53) Williams C, Jones M, Swerdlow A, Botting B, Davies M, Jacobs I, et al. Risks of ovarian, breast, and corpus uteri cancer in women treated with assisted reproductive technology in Great Britain, 1991-2010: data linkage study including 2.2 million person years of observation. *British Medical Journal*. 2018;362:k2644.
(54) Yokoyama Y, Shimizu T, Hayakawa K. Incidence of handicaps in multiple births and associated factors. *Acta geneticae medicae et gemellologiae: twin research*. 1995;44(2):81-91.
(55) Doyle P. The outcome of multiple pregnancy. *Human Reproduction*. 1996;11(suppl_4):110-20.

For peer review only

(56) Elm Ev, Altman DG, Egger M, Pocock SJ, Gøtzsche PC, Vandenbroucke JP. Strengthening the reporting of observational studies in epidemiology (STROBE) statement: guidelines for reporting observational studies. *British Medical Journal*. 2007;335(7624):806-8.
(57) Castillo CM, Horne G, Fitzgerald CT, Johnstone ED, Brison DR, Roberts SA. The impact of IVF on birthweight from 1991 to 2015: a cross-sectional study. *Human Reproduction*. 2019;34(5):920-31.
(58) Pelkonen S, Koivunen R, Gissler M, Nuojua-Huttunen S, Suikkari A-M, Hydén-Granskog C, et al. Perinatal outcome of children born after frozen and fresh embryo transfer: the Finnish cohort study 1995–2006. *Human Reproduction*. 2010;25(4):914-23.
(59) Roy TK, Bradley CK, Bowman MC, McArthur SJ. Single-embryo transfer of vitrified-warmed blastocysts yields equivalent live-birth rates and improved neonatal outcomes compared with fresh transfers. *Fertility and Sterility*. 2014;101(5):1294-301. e2.
(60) Litzky JF, Boulet SL, Esfandiari N, Zhang Y, Kissin DM, Theiler RN, et al. Effect of frozen/thawed embryo transfer on birthweight, macrosomia, and low birthweight rates in US singleton infants. *American Journal of Obstetrics and Gynecology*. 2018;218(4):433.e1-.e10.
(61) Berntsen S, Pinborg A. Large for gestational age and macrosomia in singletons born after frozen/thawed embryo transfer (FET) in assisted reproductive technology (ART). *Birth Defects Res*earch. 2018;110(8):630-43.
(62) Terho AM, Pelkonen S, Opdahl S, Romundstad LB, Bergh C, Wennerholm UB, et al. High birth weight and large-for-gestational-age in singletons born after frozen compared to fresh embryo transfer, by gestational week: a Nordic register study from the CoNARTaS group. *Human Reproduction*. 2021.
(63) Elias FTS, Weber-Adrian D, Pudwell J, Carter J, Walker M, Gaudet L, et al. Neonatal outcomes in singleton pregnancies conceived by fresh or frozen embryo transfer compared to spontaneous conceptions: a systematic review and meta-analysis. *Archives of Gynecology and Obstetrics*. 2020;302(1):31-45.
(64) Maheshwari A, Pandey S, Amalraj Raja E, Shetty A, Hamilton M, Bhattacharya S. Is frozen embryo transfer better for mothers and babies? Can cumulative meta-analysis provide a definitive answer? *Human Reproduction Update*. 2018;24(1):35-58.
(65) Sha T, Yin X, Cheng W, Massey IY. Pregnancy-related complications and perinatal outcomes resulting from transfer of cryopreserved versus fresh embryos in vitro fertilization: a meta-analysis. *Fertility and Sterility*. 2018;109(2):330-42.e9.
(66) Castillo CM, Harper J, Roberts SA, O'Neill HC, Johnstone ED, Brison DR. The impact of selected embryo culture conditions on ART treatment cycle outcomes: a UK national study. *Human Reproduction Open*. 2020;2020(1):hoz031.
(67) Berntsen S, Söderström-Anttila V, Wennerholm UB, Laivuori H, Loft A, Oldereid NB, et al. The health of children conceived by ART: 'the chicken or the egg?'. *Human Reproduction Update*. 2019;25(2):137-58.
(68) Luke B, Brown MB, Wantman E, Stern JE, Toner JP, Coddington CC, 3rd. Increased risk of large-for-gestational age birthweight in singleton siblings conceived with in vitro fertilization in frozen versus fresh cycles. *Journal of Assisted Reproduction and Genetics*. 2017;34(2):191-200.
(69) Henningsen AK, Pinborg A. Birth and perinatal outcomes and complications for babies conceived following ART. *Seminars in Fetal & Neonatal Medicine*. 2014;19(4):234-8.
(70) Pinborg A, editor To transfer fresh or thawed embryos? *Seminars in Reproductive Medicine*; 2012: Thieme Medical Publishers.
(71) Kimber SJ, Sneddon SF, Bloor DJ, El-Bareg AM, Hawkhead JA, Metcalfe AD, et al. Expression of genes involved in early cell fate decisions in human embryos and their regulation by growth factors. *Reproduction*. 2008;135(5):635-47.
(72) Goldstein H. Factors related to birth weight and perinatal mortality. *British Medical Bulletin*. 1981;37(3):259-64.
(73) Harron K, Dibben C, Boyd J, Hjern A, Azimaee M, Barreto ML, et al. Challenges in administrative data linkage for research. *Big Data & Society*. 2017;4(2):2053951717745678.

For peer review only

(74) Human Fertilisation and Embryology Authority. *How we manage your information*. https://www.hfea.gov.uk/choose-a-clinic/how-we-manage-your-information/#:~:text=Key%20facts%20Researchers%20need%20your,not%20publish%20your%20identifying%20information.
(75) Spencer SA, Davies MP. Hospital episode statistics: improving the quality and value of hospital data: a national internet e-survey of hospital consultants. *BMJ Open*. 2012;2(6).
(76) Shenfield F, de Mouzon J, Pennings G, Ferraretti AP, Andersen AN, de Wert G, et al. Cross border reproductive care in six European countries. *Human Reproduction*. 2010;25(6):1361-8.

For peer review only

FIGURE LEGENDS

Figure 1: Flowchart showing linkage 1 (HFEA-ONS)

Figure 2: Flowchart showing linkage 2 (HFEA-ONS-HES)

For peer review only

Figure 1: Flowchart showing linkage 1 (HFEA-ONS)

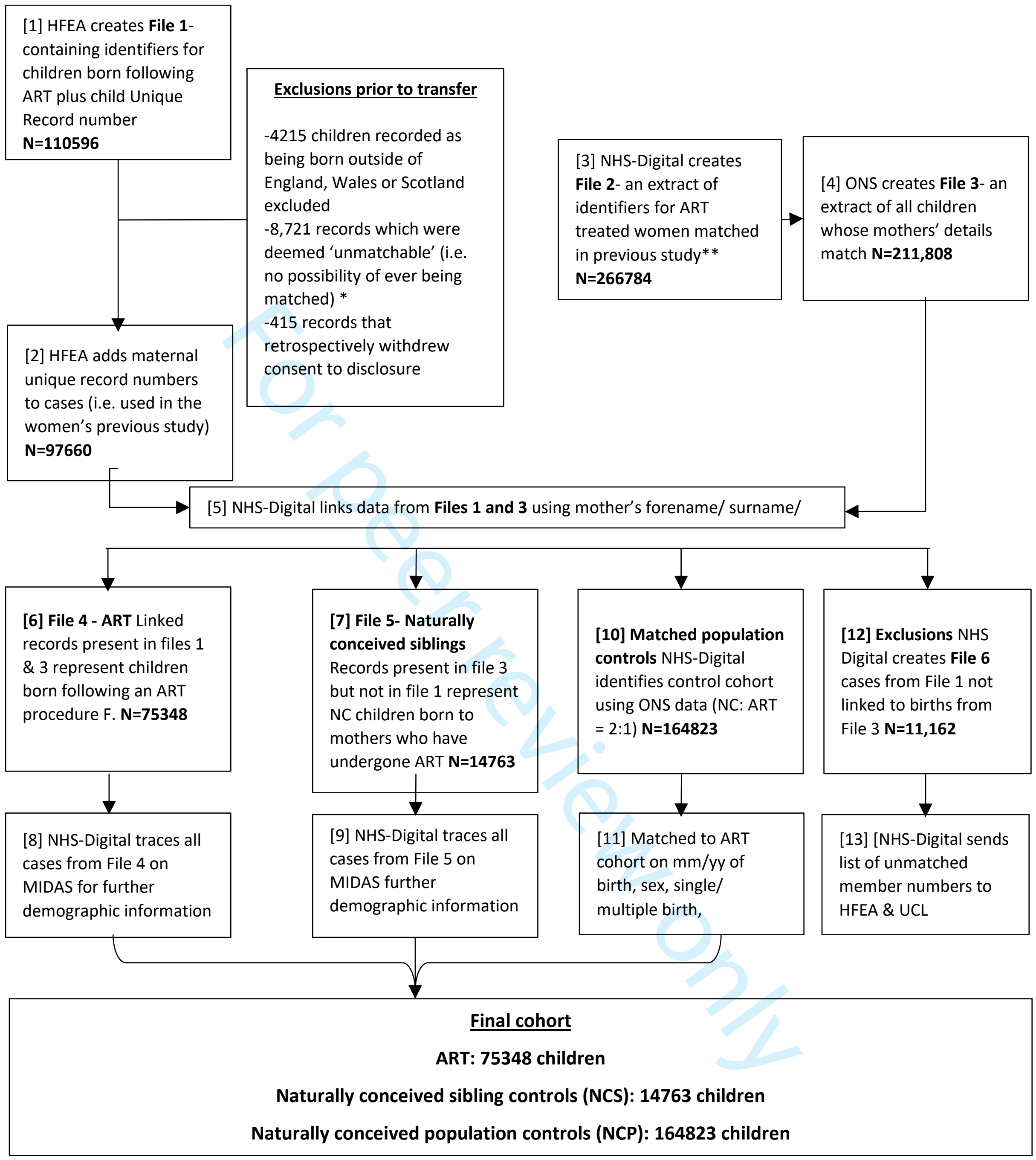

*as they were a) births outside of England/ Wales; b) births before 1993 (when ONS systems were automated and thus the date from which linkage is possible to ONS records); and c) to mothers which were not included in file 2 (as it was not possible to identify them on NHS-Digital systems previously- 'women's study').
** Please see Supplementary figure S2 for cohort flow

For peer review only

Figure 2: Flowchart showing linkage 2 (HFEA-ONS-HES)

[14] NHS Digital produces current status report on all ART, NCS and NCP controls (as produced in linkage 1) and provides de-identified output plus non identifiable deprivation score to UCL.

↓

[15] NHS Digital links cohort data to HES data and provides de-identified output to UCL

↓

[16] UCL interacts with HFEA to obtain fertility treatment clinical data and parental data per matched group member.

UCL match up NHS Digital and clinical HES information to HFEA information using unique record number.

↓

[17] UCL excludes records of children born before 1st April 1997 to coincide with start of HES monitoring.

UCL excludes triplets and higher order births from all groups (along with associated ART, siblings and matched controls)

↓

[18] NHS Digital produces file 4 for each group (identifiable details as below).

- For the ART group, file 4 will be securely transported to the HFEA and stored there securely with highly restricted access.
- For the NCS and NCP groups, file 4 will be securely stored at NHS Digital again with highly restricted access.

↓

Final sub-cohort

HES-ART: 63877

Naturally conceived siblings controls (HES-NCS): 11343

Naturally conceived population controls (HES-NCP): 127544

HES: Hospital Episode Statistics database; UCL: University College London; HFEA: Human Fertilization and Embryology Authority

For peer review only - http://bmjopen.bmj.com/site/about/guidelines.xhtml

Supplementary Files

S1: Completeness of variables (for the purpose of linkage and analysis) on the HFEA register

Variable	Useable* entry	Entry 'Null'	No entry	Other**	% useable out of total no. of entries
DOB	110,204	374	0	18	99.6%
Sex of Child<br>*Male*<br>*Female*	110,484<br>*56,265*<br>*54,219*	112	0	0	99.9%
BW	109, 157	534	0	905 records with BW <600g ***	98.7%
Child's Surname	12,332	97,671	0	593	11.2%
Child's Forename	11,402	97,670	0	1524	10.3%
Child's Town of Birth	70,738	39,689	61	108	64.0%
Child's District of Birth	18,833	91,452	298	13	17.0%
Child's Town ***or*** District of Birth	71,650	38,787	51	108	64.8%
Country of Birth	67554	38,861	0	4181 cases born outside of Great Britain	61.1%
Mother's Surname	110,596	0	0	0	100%
Mother's Forename	110,588	0	8	0	100.0%
Mother's Surname at Birth	51,237	58,714	209	436	46.3%
Mother's Forename at Birth	3,323	106,895	377	1	3.0%
Mother's DOB	110,569	1	0	26	100.0%
Father's Surname	110,450	138	8	0	99.9%
Father's Forename	110,384	202	8	2	99.8%
Father's DOB	110,282	257	0	57	99.7%
Mother's Town or District of Birth	62,505	47,045	0	1046	56.5%
Mother's Country of Birth	64,396	46,190	0	10	58.2%
Father's Town or District of Birth	61,143	48,354	0	1099	55.3%
Father's Country of Birth	63,100	47,485	0	11	57.1%
Treatment Cycle Start Date	110,596	0	0	0	100%
Treatment Centre No/ Name	110,594	0	0	2	100.0%

HFEA: Human Fertilization and Embryology Authority; DOB: date of birth; BW: birth weight

* 'Useable' data refers to records which contain data that appears useable in some analyses, including initial quality control analyses.

** 'Other' refers to unusable information for the variable in question, most commonly variations of 'Not known', 'Unknown' etc.

***905 records with documented BWs less than 600g were considered as invalid information for the purpose of deterministic linkage. Whilst a small number of these BWs (<600g) are likely to be correct, many more are likely to be data errors within the HFEA register. All actual recorded BWs were used when manually viewing potential matches.

This table was shared with us by HFEA at the start of the study.

For peer review only

S2- Flow diagram showing derivation of File 2 in Figure 1.

302, 487 Human Fertilisation & Embryology (HFEA) records relating to women treated with ART, 1991-2010, were available for linkage. These were transferred to NHS-digital & National Records of Scotland for linkage to NHS central registries of England & Wales and Scotland (EWS).

13,017 duplicates

- 11,290 - 2 or more distinct unique record numbers relating to the same individual
- 1,727 - duplicate unique record numbers.
- 1,722: Exact duplicate unique record numbers
- 5: Both had an exact duplicate unique record numbers and two distinct unique record numbers

22,683 'No trace' on linkage to NHSCR

- 8, 854 - HFEA recorded address outside England, Wales and Scotland (EWS) (no possibility of linkage to NHSCR)
- 13,829 - EWS based women, no trace on NHSCR

3 records - noted to have possible linkage error after data quality checks

266,784 of 280,616 eligible records successfully linked.

- ***<u>Linkage rate = 95.07% *</u>***

* Denominator excludes duplicate records and records of women based overseas who had treatment in the UK.

242 records - contained no clinical fertility data

122 records - embarked from EWS before treatment date, thus no outcome data available

266,420 of 280,616 records successfully linked and containing both fertility and outcome data.

- 94.94%

1,678 records **-** treatment date missing

52 records – DOB missing

8,904 - Women had a cancer diagnosis prior to the first calendar year of treatment.

255,786 of 280,616 potentially eligible records entered into the cohort analysis

- 91.15%

HFEA: Human Fertilization and Embryology Authority; DOB: date of birth; EWS: England, Wales & Scotland; NHSCR: National Health Service Central Register

S3: Order of pregnancy (HFEA-ONS cohort)

No. of pregnancies	Order of pregnancy	ART		Spontaneously conceived siblings	
		Singleton pregnancy	Twin Pregnancy	Singleton pregnancy	Twin Pregnancy
**2 (n=8441)**	1	5135	1182	2095	29
	2	1596	528	6161	156
**3 (n=1336)**	1	918	107	304	7
	2	453	96	777	10
	3	126	26	1155	29
**4 (n=125)**	1	83	9	33	0
	2	42	5	77	1
	3	19	6	99	1
	4	7	4	113	1
**5 (n=12)**	1	8	1	2	1
	2	5	1	6	0
	3	2	0	10	0
	4	0	0	12	0
	5	0	0	12	0
**6 (n=1)**	1	1	0	0	0
	2	0	0	1	0
	3	0	0	1	0
	4	0	0	1	0
	5	0	0	1	0
	6	0	0	1	0
**7 (n=1)**	1	0	1	0	0
	2	0	0	1	0
	3	0	0	1	1
	4	0	0	0	0
	5	0	0	1	0
	6	0	0	1	0
	7	0	0	1	0

HFEA: Human Fertilization and Embryology Authority; ONS: Office for National Statistics.

For peer review only

S4: Multiple regression coefficients for adjusted mean BW- ART vs NCP (HFEA-ONS)

Variables	BW Coefficient	95% Confidence interval
Embryo transfer status		
Fresh	-131.4	(-139.5 - -123.4)
Frozen	35.24	(18.97 - 51.52)
Sex		
Male	119.1	(112.7 - 125.5)
Maternal age group		
25-29	66.68	(55.89 - 77.48)
30-34	111.9	(102.2 - 121.6)
35-39	129.2	(118.7 - 139.7)
40-44	132.3	(118.0 - 146.5)
>=45	72.66	(-13.46 - 158.8)
**Constant**	3,215	(3,207 - 3,223)
**Observations**	129,304	
**R-squared**	0.021	

BW: birth weight; NCP: naturally conceived population controls; HFEA: Human Fertilization and Embryology Authority; ONS: Office for National Statistics.

For peer review only

S5: Multiple regression coefficients for adjusted mean BW- sART vs NCS (HFEA-ONS)

VARIABLES	BW coefficient	95% Confidence interval
Embryo transfer status		
Fresh	-54.69	(-72.64 - -36.73)
Frozen	138.6	(101.3 - 175.9)
Sex		
Male	115.6	(97.78 - 133.4)
Maternal age group		
25-29	-43.23	(-114.2 – 27.74)
30-34	-98.50	(-176.5 - -20.46)
35-39	-117.1	(-206.8 - -27.41)
40-44	-161.8	(-266.1 - -57.52)
>=45	-198.7	(-409.2 – 11.79)
Order of pregnancy		
2	165.6	(142.2 – 188.9)
>=3	237.0	(194.4 – 279.6)
**Constant**	3,337	(3,263 - 3,411)
**Observations**	22,086	
**R-squared**	0.081	

BW: birth weight; sART: ART with siblings; NCS- naturally conceived siblings; HFEA: Human Fertilization and Embryology Authority; ONS: Office for National Statistics.

For peer review only

S6: Multiple regression coefficients for adjusted mean BW- ART vs NCP (HFEA-ONS-HES cohort)

Variables	BW coefficient	95% confidence interval
Embryo transfer status		
Fresh	-152.2	(-162.2 - -142.3)
Frozen	2.855	(-16.61 - 22.32)
Sex		
Male	120.6	(112.7 - 128.6)
Maternal age group		
25-29	57.25	(43.52 - 70.99)
30-34	86.40	(73.80 - 99.00)
35-39	91.20	(77.62 - 104.8)
40-44	98.57	(80.37 - 116.8)
45-49	4.103	(-63.56 - 71.77)
Missing	94.69	(60.19 - 129.2)
IMD at earliest appointment		
2	25.95	(8.336 - 43.57)
3	56.66	(38.69 - 74.64)
4	58.78	(40.66 - 76.91)
5	75.13	(56.93 - 93.34)
6	93.33	(75.06 - 111.6)
7	93.21	(75.17 - 111.2)
8	111.8	(93.76 - 129.8)
9	117.3	(99.48 - 135.1)
10	134.7	(116.5 - 152.8)
Derived ethnicity		
Asian/Asian British	-247.8	(-264.2 - -231.4)
Black/African/Caribbean/Black British	-138.2	(-161.2 - -115.2)
Chinese	-120.1	(-195.7 - -44.43)
Mixed/multiple ethnic groups	-66.13	(-94.92 - -37.33)
Not stated/Not known	5.034	(-5.060 - 15.13)
Other ethnic group	-49.19	(-82.25 - -16.13)
**Constant**	3,177	(3,162 - 3,192)
**Observations**	85,366	
**R-squared**	0.040	

BW: birth weight; NCP- naturally conceived population controls; HFEA: Human Fertilization and Embryology Authority; ONS: Office for National Statistics; HES: Hospital Episode Statistics; IMD: Index of Multiple Deprivation

For peer review only

S7: Multiple regression coefficients for adjusted mean BW- sART vs NCS (HFEA-ONS-HES cohort)

Variables	BW coefficient	95% Confidence interval
Embryo transfer status		
Fresh	-57.01	(-75.79 - -38.23)
Frozen	152.1	(113.5 - 190.7)
Sex		
Male	115.6	(96.95 – 134.2)
Maternal age group		
25-29	-28.73	(-104.3 - -46.84)
30-34	-81.27	(-164.9 – 2.335)
35-39	-96.01	(-192.0 - -0.010)
40-44	-135.4	(-246.9 - -23.89)
>=45	-141.6	(-308.8 - -25.69)
Order of pregnancy		
2	152.2	(127.8 – 176.6)
>=3	229.6	(184.3 – 274.8)
**Constant**	3,332	(3,252 - 3,412)
**Observations**	19180	
**R-squared**	0.09	

BW: birth weight; sART: ART with siblings; NCS- naturally conceived siblings; HFEA: Human Fertilization and Embryology Authority; ONS: Office for National Statistics.

S8: Distribution of maternal age in records born before 1997 and not linked to HES

	ART	CONTROL	SIBLING
Maternal age group			
<=25	91 (0.79%)	5804 (15.57%)	84 (2.45%)
25-29	1360 (11.84%)	5496 (14.74%)	179 (5.22%)
30-34	4781 (41.62%)	7592 (20.37%)	636 (18.55%)
35-39	4318 (37.59%)	4738 (12.71%)	873 (25.46%)
40-44	921 (8.02%)	1160 (3.11%)	357 (10.41%)
>=45	11 (0.10%)	132 (0.35%)	27 (0.79%)
Missing	5 (0.04%)	12357 (33.15%)	1273 (37.12%)

HES: Hospital Episode Statistics

For peer review only

For peer review only

For peer review only

Reporting checklist for cohort study.

Based on the STROBE cohort guidelines.

Instructions to authors

Complete this checklist by entering the page numbers from your manuscript where readers will find each of the items listed below.

Your article may not currently address all the items on the checklist. Please modify your text to include the missing information. If you are certain that an item does not apply, please write "n/a" and provide a short explanation.

Upload your completed checklist as an extra file when you submit to a journal.

In your methods section, say that you used the STROBE cohort reporting guidelines, and cite them as:

von Elm E, Altman DG, Egger M, Pocock SJ, Gotzsche PC, Vandenbroucke JP. The Strengthening the Reporting of Observational Studies in Epidemiology (STROBE) Statement: guidelines for reporting observational studies.

For peer review only

		Reporting Item	Page Number
**Title and abstract**			
Title	#1a	Indicate the study's design with a commonly used term in the title or the abstract	1 & 2

Abstract	#1b	Provide in the abstract an informative and balanced summary of what was done and what was found	2
**Introduction**			
Background / rationale	#2	Explain the scientific background and rationale for the investigation being reported	4
Objectives	#3	State specific objectives, including any prespecified hypotheses	5, 6
**Methods**			
Study design	#4	Present key elements of study design early in the paper	6,7,8
Setting	#5	Describe the setting, locations, and relevant dates, including periods of recruitment, exposure, follow-up, and data collection	7, 8
Eligibility criteria	#6a	Give the eligibility criteria, and the sources and methods of selection of participants. Describe methods of follow-up.	7, 8
Eligibility criteria	#6b	For matched studies, give matching criteria and number of exposed and unexposed	7, 8

For peer review only

Variables	#7	Clearly define all outcomes, exposures, predictors, potential confounders, and effect modifiers. Give diagnostic criteria, if applicable	7, 8
Data sources / measurement	#8	For each variable of interest give sources of data and details of methods of assessment (measurement). Describe comparability of assessment methods if there is more than one group. Give information separately for for exposed and unexposed groups if applicable.	6,7
Bias	#9	Describe any efforts to address potential sources of bias	10<br>Creation of two control cohorts to minimize bias
Study size	#10	Explain how the study size was arrived at	N/A<br>Population-based study.
Quantitative variables	#11	Explain how quantitative variables were handled in the analyses. If applicable, describe which groupings were chosen, and why	11

For peer review only

Statistical methods	#12a	Describe all statistical methods, including those used to control for confounding	10, 11
Statistical methods	#12b	Describe any methods used to examine subgroups and interactions	11
Statistical methods	#12c	Explain how missing data were addressed	8-11
Statistical methods	#12d	If applicable, explain how loss to follow-up was addressed	N/A
Statistical methods	#12e	Describe any sensitivity analyses	11
**Results**			
Participants	#13a	Report numbers of individuals at each stage of study—eg numbers potentially eligible, examined for eligibility, confirmed eligible, included in the study, completing follow-up, and analysed. Give information separately for for exposed and unexposed groups if applicable.	11, 12, Figure 1 & 2
Participants	#13b	Give reasons for non-participation at each stage	N/A
Participants	#13c	Consider use of a flow diagram	Figure 1 & 2

For peer review only

Descriptive data	#14a	Give characteristics of study participants (eg demographic, clinical, social) and information on exposures and potential confounders. Give information separately for exposed and unexposed groups if applicable.	13, 14, 16 ,17
Descriptive data	#14b	Indicate number of participants with missing data for each variable of interest	13, 14, 16 ,17
Descriptive data	#14c	Summarise follow-up time (eg, average and total amount)	13, 14, 16 ,17
Outcome data	#15	Report numbers of outcome events or summary measures over time. Give information separately for exposed and unexposed groups if applicable.	13, 14, 16 ,17
Main results	#16a	Give unadjusted estimates and, if applicable, confounder-adjusted estimates and their precision (eg, 95% confidence interval). Make clear which confounders were adjusted for and why they were included	17, 18
Main results	#16b	Report category boundaries when continuous variables were categorized	13, 14, 16 ,17

For peer review only

Main results	#16c	If relevant, consider translating estimates of relative risk into absolute risk for a meaningful time period	N/A
Other analyses	#17	Report other analyses done—eg analyses of subgroups and interactions, and sensitivity analyses	N/A
**Discussion**			
Key results	#18	Summarise key results with reference to study objectives	18
Limitations	#19	Discuss limitations of the study, taking into account sources of potential bias or imprecision. Discuss both direction and magnitude of any potential bias.	20
Interpretation	#20	Give a cautious overall interpretation considering objectives, limitations, multiplicity of analyses, results from similar studies, and other relevant evidence.	18, 19
Generalisability	#21	Discuss the generalisability (external validity) of the study results	20
**Other Information**			

For peer review only

Funding	#22	Give the source of funding and the role of the funders for the present study and, if applicable, for the original study on which the present article is based	22

None The STROBE checklist is distributed under the terms of the Creative Commons Attribution License CC-BY. This checklist can be completed online using https://www.goodreports.org/, a tool made by the EQUATOR Network in collaboration with Penelope.ai

For peer review only

BMJ Open

Cohort Profile: A national, population based cohort of children born after assisted conception in the UK (1992-2009) - Methodology and birthweight analysis

Journal:	*BMJ Open*
Manuscript ID	bmjopen-2021-050931.R2
Article Type:	Cohort profile
Date Submitted by the Author:	23-Jun-2021
Complete List of Authors:	Purkayastha, Mitana; UCL Great Ormond Street Institute of Child Health Population Policy and Practice<br>Roberts, Stephen; University of Manchester, Centre for Biostatistics,Institute of Population Health<br>Gardiner, Julian; University of Oxford, Education; UCL Great Ormond Street Institute of Child Health Population Policy and Practice,<br>Brison, Daniel; Central Manchester University Hospitals NHS Foundation Trust, Department of Reproductive Medicine<br>Nelson, Scott; University of Glasgow, School of Medicine<br>Lawlor, Deborah; University of Bristol Bristol Population Health Science Institute, MRC Integrative Epidemiology Unit<br>Luke, Barbara ; Michigan State University,<br>Sutcliffe, Alastair; UCL Great Ormond Street Institute of Child Health Population Policy and Practice
**Primary Subject Heading**:	Paediatrics
Secondary Subject Heading:	Epidemiology, Reproductive medicine
Keywords:	Subfertility < GYNAECOLOGY, EPIDEMIOLOGY, PAEDIATRICS

SCHOLARONE™
Manuscripts

I, the Submitting Author has the right to grant and does grant on behalf of all authors of the Work (as defined in the below author licence), an exclusive licence and/or a non-exclusive licence for contributions from authors who are: i) UK Crown employees; ii) where BMJ has agreed a CC-BY licence shall apply, and/or iii) in accordance with the terms applicable for US Federal Government officers or employees acting as part of their official duties; on a worldwide, perpetual, irrevocable, royalty-free basis to BMJ Publishing Group Ltd ("BMJ") its licensees and where the relevant Journal is co-owned by BMJ to the co-owners of the Journal, to publish the Work in this journal and any other BMJ products and to exploit all rights, as set out in our licence.

The Submitting Author accepts and understands that any supply made under these terms is made by BMJ to the Submitting Author unless you are acting as an employee on behalf of your employer or a postgraduate student of an affiliated institution which is paying any applicable article publishing charge ("APC") for Open Access articles. Where the Submitting Author wishes to make the Work available on an Open Access basis (and intends to pay the relevant APC), the terms of reuse of such Open Access shall be governed by a Creative Commons licence – details of these licences and which Creative Commons licence will apply to this Work are set out in our licence referred to above.

Other than as permitted in any relevant BMJ Author's Self Archiving Policies, I confirm this Work has not been accepted for publication elsewhere, is not being considered for publication elsewhere and does not duplicate material already published. I confirm all authors consent to publication of this Work and authorise the granting of this licence.

For peer review only

Cohort Profile: A national, population based cohort of children born after assisted conception in the UK (1992-2009) - Methodology and birthweight analysis

Purkayastha M[a] [ORCID ID: https://orcid.org/0000-0002-1870-8838; email: m.purkayastha@ucl.ac.uk]

Roberts S.A[b] [ORCID ID: https://orcid.org/0000-0002-7477-7731; email: Steve.Roberts@manchester.ac.uk]

Gardiner J[c] [ORCID ID: https://orcid.org/0000-0001-8988-3812; email: julian.gardiner@education.ox.ac.uk]

Brison D.R[d] [ORCID ID: https://orcid.org/0000-0002-4307-1293; email: daniel.brison@manchester.ac.uk]

Nelson S.M[e] [ORCID ID: https://orcid.org/0000-0002-9099-3047; email: Scott.Nelson@glasgow.ac.uk]

Lawlor D.A[f] [ORCID ID: https://orcid.org/0000-0002-6793-2262; email: D.A.Lawlor@bristol.ac.uk]

Luke B[g] [ORCID ID: https://orcid.org/0000-0002-0405-8133; email: barbara.luke@hc.msu.edu]

Sutcliffe A.G[a] [ORCID ID: https://orcid.org/0000-0001-8542-6155; email: a.sutcliffe@ucl.ac.uk]

Author Affiliations:

[a] Great Ormond Street Institute of Child Health, University College London

[b] Division of Population Health, Health Services Research & Primary Care, University of Manchester

[c] Department of Education, University of Oxford

[d] Division of Developmental Biology & Medicine, University of Manchester

[e] School of Medicine, Dentistry & Nursing, Reproductive & Maternal Medicine, University of Glasgow

[f] Bristol Population Health Science Institute, University of Bristol

[g] Obstetrics, Gynaecology and Reproductive Biology,College Of Human Medicine, Michigan State University

Corresponding Author:

Mitana Purkayastha

30 Guilford Street

Population, Policy, and Practice

UCL Great Ormond Street Institute of Child Health

London WC1N 1DP

Email: m.purkayastha@ucl.ac.uk

Word count: 6193

For peer review only

ABSTRACT

Purpose: To generate a large cohort of children born after assisted reproductive technology (ART) in the UK between 1992 and 2009, their naturally conceived siblings (NCS), and matched naturally conceived population (NCP) controls and linking this with health outcome data to allow exploration of the effects of ART. The effects of fresh and frozen embryo transfer on birth weight were analysed to test the validity of the cohort.

Participants: Children recorded on the Human Fertilisation & Embryology Authority (HFEA) register as being born after ART between 1992 and 2009, their NCS, and matched NCP controls linked to Office for National Statistics birth registration dataset (HFEA-ONS cohort). This cohort was further linked to the UK Hospital Episode Statistics database to allow monitoring of the child's post-natal health outcomes up to 2015 (HFEA-ONS-HES sub-cohort).

Findings to date: The HFEA-ONS cohort consisted of 75348 children born after non-donor ART carried out in the UK between 1st April 1992 and 31st July 2009 and successfully linked to birth registration records, 14763 NCS and 164823 matched NCP controls. The HFEA-ONS-HES sub-cohort included 63877 ART, 11343 NCS, and 127544 matched NCP controls further linked to health outcome data. The exemplar analysis showed that children born after fresh embryo transfers were lighter (BW difference: -131g, 95%CI: -140, -123) and those born after frozen embryo transfers were heavier (BW difference: 35g, 95%CI: 19, 52) than the NCP controls. The within-sibling analyses were directionally consistent with the population control analyses, but attenuated markedly for the fresh vs natural conception (BW difference: -54g; 95% CI: -72, -36) and increased markedly for the frozen vs natural conception (BW difference: 152g; 95% CI: 113, 190) analyses.

Future plans: To utilise this cohort to explore the relationship between ART conception and short- and long-term health outcomes in offspring.

Keywords: assisted reproductive technology; birth weight; naturally conceived siblings; naturally conceived population controls; record linkage

For peer review only

Strengths and limitations of this study

- Meticulous linkage of robust, routinely collected administrative health data to yield a large cohort that is nationally unique, thus increasing the generalisability, accuracy, and precision of results from subsequent analyses.

- Linkage to the hospital admissions and outpatient database provides long-term mortality and morbidity outcome data on offspring for use in longitudinal research, policy planning and strategic development.

- Identification of naturally conceived siblings as well as matched naturally conceived population controls allows exploration of the association of ART with adverse offspring outcomes while accounting for parental factors related to sub-fertility which may confound these associations.

- Comparison of findings between the two approaches (ART vs naturally conceived population controls & ART vs naturally conceived siblings) mentioned above increases confidence in findings.

- The validity of the cohort was tested by means of an exemplar analysis.

For peer review only

INTRODUCTION

Assisted reproductive technology (ART) usage has increased annually since the first live birth in 1978, with over 8 million children born after ART globally (1). This increased utilization of ART has occurred concurrently with developments in our understanding of the impact of the early life environment on long term health (2-4) and most families with ART conceived children reported potential general health risks to their children as their paramount concern (5, 6), resulting in a concomitant increase in focus on the potential adverse short- and long-term effects on offspring. The inability to distinguish the relative importance of the effects of ART treatment factors and parental sub-fertility is a common limitation of many ART follow-up studies (7-9). This issue has been addressed in prospective cohorts by identifying control populations of children born naturally to parents with established subfertility [different from infertility in terms of the time of unwanted non-conception (10)]. One such well-known cohort provided the first evidence of differences in blood pressure and growth parameters in ART children (11, 12). Further evidence comes from within-ART comparisons showing that early life environment (e.g. embryo culture medium composition) is associated with changes in fetal growth, birth weight (BW) and child growth (13, 14), and studies of embryo cryopreservation have made a similar point more recently (15). These small studies have been essential in both directing and validating the associations identified from large population studies, including prospective cohort studies of ART conceptions only (e.g a Swedish study that followed-up 30,959 children born after ART) (16) or large, record linkage studies (e.g. one South Australian record linkage study compared 6163 ART and 302,811 naturally conceived (NC) offspring, while another study in Denmark compared 33,139 ART children to 555828 NC children from the population) (17, 18). The Committee of Nordic ART and Safety (CoNARTaS), created in 2008, utilised medical registry data from Denmark, Finland, Norway and Sweden to establish a large cohort of children conceived after ART or natural conception as well as women with at least one delivery after ART or natural conception, with the aim of examining long-term health outcomes in children born after ART (19-21).

While these large studies using conventional multivariable approaches to explore the association of ART with adverse offspring outcomes are essential in order to extrapolate effect sizes and risk estimates to the general ART population (22-24), they cannot fully account for parental factors related to sub-fertility which may confound these associations. This has been addressed in record linkage studies that used within sibling analyses (where comparisons are made between ART and their NC siblings) to better control for factors related to sub-fertility and other family confounders under the assumption that these parental factors would be the same (or very similar) within sibling

For peer review only

groups (23, 25). However, such analyses typically have lower statistical power due to restricted numbers, can be biased if there is individual level confounding, and the subset of ART children with siblings may not be representative of the ART population as a whole. Therefore, a comparison of the two approaches is valuable as similar results from both would increase confidence in findings (25-27).

In the United Kingdom, birth rates from IVF treatment have increased by over 85% since 1991, with around one in three treatment cycles now resulting in a birth for patients under 35 (28). It has been compulsory for every fertility clinic in the UK to report details of all treatments carried out to the Human Fertilisation & Embryology Authority (HFEA) since its inception in August 1991 (29, 30, 31). Due to the mandatory nature of reporting, the completeness and quality of data related to some couple characteristics (e.g. age, duration of infertility) and ART procedures is high, enabling research analysing treatment outcomes including success rates and perinatal outcomes such as gestation, BW, and congenital anomalies (32-37). However, the quality of perinatal outcome data available on the HFEA register is questionable as it is patient reported via the ART clinics (38). Hann et al (2018) (39) showed that 1 in 15 (6%) BWs recorded on the HFEA register were incorrect compared to data recorded directly by the delivering maternity unit. Moreover, the HFEA register contains no information on the health outcomes of children beyond the immediate perinatal period.

A change in the law in 2009 (40) made it possible for researchers to use patient identifying data contained in the HFEA register from 1991-2009 for the purpose of linkage to offspring health outcome databases, thus allowing the largest ever population studies of ART childhood cancer (41) and early child growth (39). However, further studies have been limited by extensive data governance requirements and high associated costs, and prospective monitoring of outcomes in this population as they progress from childhood into adulthood has been prevented thus far by the paucity of identifiable information necessary for a variety of linkages.

Consequently, the primary objective of this study was to substantially enhance the research value of the HFEA register by utilising electronic record linkage methodology to establish a cohort of children consisting of those born after ART in the UK between 1992 and 2009, their naturally conceived siblings (NCS) and matched naturally conceived population (NCP) controls to allow exploration of outcomes present on the birth registration dataset and subsequent linkage to other datasets. The secondary objective was to create a sub-cohort of children born after ART in the UK, NCS and matched NCP controls and linking this with information on their postnatal health outcomes up to 2015. These databases will be made available to all researchers (subject to necessary approvals), allowing more precise risk estimates of associations of ART with many potential childhood (and maternal) outcomes. Finally, the third objective of this study was to validate the cohort and sub-

For peer review only

cohort produced by comparing the effects of fresh and frozen embryo transfer versus natural conception on singleton BW. The hypothesis being tested was that children born after frozen embryo transfers would be heavier and those born after fresh embryo transfers would be lighter than those that were naturally conceived. Our previous linkage from the HFEA 1991-2009 cohort to maternity and child growth records confirmed evidence in the field that children born after frozen embryo transfers are heavier and those born after fresh embryo transfers are lighter than those that are naturally conceived (NC), and that these differences in BW are further associated with differences in child growth up to the age of 5 (39). However, this study was only able to examine the subset of ART children born in Scotland and the dataset created was not readily linkable to other datasets.

COHORT DESCRIPTION

Databases used

Human Fertilization & Embryology Authority Register (HFEA): The HFEA, an 'arm's length body' of the Department of Health, acts as an independent regulator of fertility treatment and research using human embryos in the UK (31). All licensed fertility clinics in the UK are required by law to provide information to the HFEA about treatments they carry out and their outcomes, ensuring high levels of data completeness. Only 1500 ART outcomes had not been reported as of 07/01/2020, almost all of which were likely to be from overseas patients who had ART in the UK and returned home for the delivery making it difficult for clinics to follow up (42). Regular quality assurance checks including manual validation of data submissions; regular quality assurance checks on data through the inspection process; publication of non-compliances with data quality issues in inspection reports; and, where relevant, review of quality reports and auditing of clinics with irregular data submissions are also carried out (43).

The HFEA Act 1990 (29) made prospective collection and storage of baseline information and birth outcomes on the HFEA register mandatory, although 'consent for disclosure of information for research' was not collected from patients who underwent treatment at a licensed fertility clinic prior to September 2009. The reliability and completeness of information relating to women who have undergone treatment on the HFEA register is considerably high (approximately 99.9%, 100% & 99.9% completeness for forename, surname, & DOB, respectively; Supplementary table S1), although it contains little identifiable information on children born after ART (e.g 11.2% and 10.3% completeness for child's surname and forename, respectively; Supplementary table S1).

Details of the data accessed from HFEA for this study are discussed below (see Linkage section).

For peer review only

Birth Registration Database*:* In the UK, all births are legally required to be registered by the Local Registration Service in partnership with the General Register Office (GRO) in England and Wales, making it the most complete data source available (44). The birth registration dataset is managed by ONS in England and Wales, while Scottish birth records are held by National Records for Scotland (NRS) who work closely with NHS Digital to ensure daily record transfers between the organisations using a secure and closed electronic system.

This study used the birth registration dataset to identify all children born to women known to have undergone ART as well as their NCS, and details of the data accessed have been discussed below (see Linkage section).

Hospital Episode Statistics (HES): Hospital Episode Statistics (HES) is a data warehouse containing details of all admissions, outpatient appointments and A & E attendances at NHS hospitals in England (45). HES data covers all NHS Clinical Commissioning Groups (CCGs) in England, including private patients treated in NHS hospitals, patients resident outside of England, and care delivered by treatment centres (including those in the independent sector) funded by the NHS (45). Approximately 98–99% of hospital activity in England are estimated to be funded by the NHS (46). Moreover, the HES admitted patient care database covers all births in NHS hospitals, representing approximately 97.3% of births in England, thus enabling creation of nationally representative birth cohorts (47, 48).

Although the HES data warehouse has been operating since 1990 (49), linkage of the HES admitted patient care (APC) episodes longitudinally to the same individuals only commenced in 1997/98 when the patient's NHS number became a mandated return from hospitals (50). Furthermore, linkage of this APC dataset to other HES datasets such as outpatient, A & E, and adult critical care only commenced much later in 2003/2004, 2007/2008 and 2008/2009, respectively (50). HES diagnoses were coded using the International Classification of Diseases version 9 (ICD-9) between April 1989 and March 1995 and ICD-10 thereafter.

This study linked the HES database to the ART children, NCS, and matched NCP control groups, and the data accessed for this purpose have been discussed below (see Linkage section). Although the current paper only uses demographic data [ethnicity; UK census-derived Index of Multiple Deprivation (IMD), the official measure of relative deprivation for small areas or neighbourhoods in the UK (51)] and BW from HES to carry out an exemplar analysis, further analyses of longitudinal health outcomes in these cohorts are already underway and will be published shortly.

For peer review only

Medical Integrated Database and Administration System (MIDAS): MIDAS is a system used by NHS Digital's Data Linkage and Extract Service (DLES) to replace the functionality previously provided by the Central Health Register Information System (CHRIS) application, specifically for the administration of information used within research and data linkage functions. It was developed to provide continuity for the existing list cleaning, flagging, tracing and cohort management services to maintain support for approved research and audit projects, National Cancer Registration and the ONS Longitudinal Study. It contains up to date demographic GP registration data via a daily feed from the Secondary User Service, standard demographic identifiers such as names and addresses, information on patients who have exited from and returned to the NHS, as well as cancer and death registration details. MIDAS can be considered as a window into the Personal Demographics Service database, providing bespoke data extracts in addition to regular patient tracking services. Extracts are provided on an individual as-and-when basis, and are subject to the regular DLES approvals process.

In this study, the cohort produced by Linkage 1 (discussed later) was traced on MIDAS for further demographic information (NHS number where necessary, postcode etc.) that would allow subsequent linkage to HES. Additionally, the matched NCP controls were also identified using MIDAS.

Inclusion and Exclusion criteria

ART: defined as "treatments or procedures that include in vitro handling of both human oocytes and sperm or embryos, for the purpose of reproduction" (52). This study included all children born after non-donor ART conducted in the UK between 1st April 1992 and 31st July 2009, in keeping with legislative changes that influenced the ability to link HFEA data to other health datasets (39, 41). It was estimated that only 0.2% of patients receiving ART during the study period retrospectively withdrew consent (53).

NCS**:** All naturally conceived (NC) children born to women with at least one child born following ART were included. This comprised of both full (both parents the same) and maternal half-siblings (same mother different father), but identification of paternal half-siblings was not possible.

NCP controls**:** The birth registration dataset and MIDAS were used to identify two matched [by month and year of birth, sex, and multiplicity/plurality] NCP controls per ART child (ART: control = 1:2).

For peer review only

All children conceived in the UK after ART but born outside of England, Wales & Scotland, those born after ART to women who permanently lived outside the UK but travelled to the UK for ART treatment, and those born in Northern Ireland were excluded as it would not be possible to link them to ONS birth records. Additionally, siblings born outside of the study period (as their conception status could not be verified) as well as those born outside of England, Wales & Scotland were also excluded. Cases that had withdrawn consent for their data to be used for research and children born after donor ART were excluded in compliance with HFEA legislation which prevents the viewing of identifiable data relating to these children by any third party.

Linkage

As described above, the HFEA Register contains very few identifiers for children born after ART, thus limiting linkage to other nationally held datasets. Therefore, this study initially linked HFEA records of children born to women known to have undergone ART to nationally held birth records to add identifiers and NHS numbers which would enable further linkage with a variety of other datasets (shown in Figures 1 & 2).

Linkage 1: HFEA-ONS

Step 1 [1 & 2 from Figure 1]: The HFEA first identified children born after ART and assigned them pseudo-anonymised unique record numbers (URN). Maternal unique record numbers (mURN; created from a previous study (53) [Supplementary Table S2] were added to the file, which was encrypted and sent securely to NHS Digital.

This file contained the following variables: date of birth (DOB) of child, BW of child, sex of child, forename of mother, surname of mother, previous name(s) of mother, multiplicity/plurality (e.g., singleton, twin), and mother's DOB.

Step 2 [3 & 4 from figure 1]: NHS Digital created file 2 [from a previous study (53); Supplementary Table S2] containing extracts of women who had undergone ART within the specified time period and securely transferred it to ONS. This file contained the NHS Number, DOB of possible mother, surname of possible mother, forename of possible mother, other name(s) of possible mother, and the HFEA mURN.

ONS then used these variables to link to birth records to create an extract of all children born to these mothers within the specified time frame. This extract (File 3) contained the mothers' details as supplied in File 2, surname of the child, forename of the child, DOB of the child, NHS number of the child (if available), BW of the child, and sex of the child. This file was then securely transferred back to NHS Digital.

For peer review only

Step 3 [5, 6, 7, 8 & 9 from figure 1]: NHS Digital then matched files 1 & 3 to determine children born to these mothers after ART and their NCS. This linkage was carried out using DOB of child, NHS number of child (if available), BW of child, sex of child, surname of child (where available on HFEA file 1), and forename of child (where available on HFEA file 1).

The ART and sibling cohorts were then traced using NHS Digital's national Medical Integrated Database and Administration System (MIDAS) to update demographic information (including NHS number, postcode) and current status (emigrations/ deaths etc.) and allow subsequent linkage to medical records.

Step 4 [10 &11 from figure 1]: NHS Digital used MIDAS to identify 2 controls for every cohort member, matched for month and year of birth, sex, and multiplicity/plurality (singleton/ multiple birth).

Step 5 [14 from figure 1]: NHS Digital produced updated demographic information on all ART children, siblings and controls (as obtained from MIDAS) and provided de-identified outputs plus non-identifiable deprivation scores to the research team at University College London (UCL).

Linkage 2: HFEA-ONS-HES

Step 6 [15 on figure 2]: NHS Digital then linked all cohorts produced by linkage 1 to HES using NHS number, postcode, and DOB, and shared with the research team at UCL.

Step 7 [16 on figure 2]: HFEA staff extracted relevant anonymised fertility data and this was shared with the research team at UCL who linked these two files (from steps 6 and 7) using the URNs.

Step 8 [17 on figure 2]: For the purpose of this second linkage, UCL researchers excluded records of children born before 1st April 1997 to coincide with the start of HES monitoring as well as triplets and higher order births from all groups (along with associated ART and NC births) to produce the final cohorts for health outcome analysis.

Step 9 [18 on figure 2]: NHS Digital produced file 4 (shown in Figure 1) for the ART and NC cohorts and these are now securely stored at HFEA and NHS Digital, respectively.

The entire linkage process was completed by NHS-Digital and none of the researchers had access to any identifiable participant data. The details of the ART and NC groups were encrypted and are currently held securely at HFEA and NHS-Digital, respectively.

For peer review only

Cohorts produced

Linkage 1: HFEA-ONS cohort

a) *ART*: This group consisted of all children born after non-donor ART conducted in the UK between 1st April 1992 and 31st July 2009.

b) *NCS:* This included all NC children born to women who had at least one child born following ART. This included full and maternal half-siblings but not paternal half-siblings.

c) *NCP:* This consisted of two matched (by month and year of birth, sex, and multiplicity/plurality) NCP controls for each ART child.

Linkage 2: HFEA-ONS-HES sub-cohort

a) *ART with HES outcomes (HES-ART):* This included all children born after non-donor ART conducted in the UK between 1st April 1997 and 31st July 2009 linked to their HES outcomes.

b) *NCS with HES outcomes (HES-NCS):* This included the NCS of all non-donor ART children born in the UK between 1st April 1997 and 31st July 2009 linked to their HES outcomes.

c) *NCP control with HES outcomes (HES-NCP):* This included two matched NCP controls for each non-donor ART child born in the UK between 1st April 1997 & 31st July 2009 linked to their HES outcomes.

Data Cleaning

Data cleaning included deletion of triplets and higher order births along with their NCS and matched NCP controls; deletion of duplicates; and reformatting, labelling and creation of new variables. Triplets and higher order births were excluded from analysis as they are known to be associated with adverse perinatal outcomes such as higher infant mortality, birth defects, premature birth, and low BW (54, 55). Initial data scoping also revealed several discrepancies in the data, a common limitation of large data linkage studies utilising multiple data sources, and pragmatic rules were employed to derive a consensus. Additional data cleaning carried out on the sub-cohort produced via linkage 2 included creation of an additional group consisting of ART children who had NCS (HES-sART) for the purposes of analysis. Order of pregnancy was calculated by sorting the child DOBs within each family in ascending order (Supplementary tables S3), and the type of ART and cause of infertility variables were regrouped appropriately.

For peer review only

Exemplar analysis

An exemplar analysis examining the effect of fresh and frozen embryo transfer (each compared to NC) on singleton BW was carried out using the HFEA-ONS cohort and HFEA-ONS-HES sub-cohort (inclusion criteria discussed previously). Details of BW are provided by the hospital where the birth took place or by the midwife/doctor in attendance at the birth, and is then passed to ONS as a consequence of the NHS birth notification being linked to the corresponding birth registration by the registrar. Multiple regression analysis was used to compare BW between children born after ART and NCP controls. The models were adjusted for maternal age at delivery and sex for the ART vs NCP analysis, and for maternal age at delivery, sex, IMD at earliest appointment, and ethnicity for the HES-ART vs HES-NCP analysis.

A family-matched model was used to compare BW between ART children and their NCS, including a family covariate to allow for within family correlations. The models were adjusted for maternal age at delivery, sex, and order of pregnancy. IMD and ethnicity were excluded from this analysis as the underlying effects they represent would have remained constant within families. The models were parameterised to directly estimate the differences between ART (fresh and frozen) and NC children. The fresh vs frozen difference was estimated from the model by construction of the appropriate contrast. All analyses were carried out using Stata v16.0.

This paper was developed in accordance with the STROBE reporting guidelines (56).

Patient and Public Involvement

No patients involved. Due to the very personal nature of the treatments involved, it was not appropriate to contact the families directly thus preventing us from involving patients or the public in the design, conduct, reporting, or dissemination plans of our research.

FINDINGS TO DATE

Final HFEA-ONS cohort

The HFEA identified 110596 children that met our inclusion criteria, of which 97660 (Figure 1) were deemed suitable for transfer to NHS Digital and subsequent linkage to ONS birth records. The linkage success rate was 77% (75348 cases out of 97660) due to the poor quality of data received from the HFEA.

A feasibility pilot study testing the validity of the linkage carried out in this study showed that the false positivity rates were very low (<0.0002%, unpublished data). Matching was done using SQL® using exact matching (design to be inclusive of all potential matches) followed by probabilistic

matching using Jaro Winkler® software. A clash was defined as a complete incompatibility of information between databases for a particular variable, with no clashes deemed as being a match. The HFEA were asked for further information in case of one clash, and more than one clash was considered to be a failed match. In the current study, the main linkage was carried out using a very high threshold for matching, and the weak/inaccurate identifiers in the HFEA register often resulted in matches that failed to pass this threshold. The majority of unlinked records could not be found due to clashes in parental identifiers and child date of birth.

As the main linkage carried out in this study was bespoke in nature, particularly with regard to identification of the naturally conceived siblings, and had not been carried out before, standardized quality assurance measures could not be applied and instead extensive manual checking of the linkage was implemented. However, due to the mandatory nature of reporting all ART cycles carried out in the UK to the HFEA, it is unlikely that the unlinked records differed significantly from those that were linked, thus minimizing the risk of selection bias.

Tables 1 & 2 shows the descriptive characteristics of the final HFEA-ONS cohort generated after completion of Linkage 1 (Figure 1) and data cleaning. This cohort included 75348 children born after ART between 1992 and 2009 and 164823 matched NCP controls. Of the children born after ART, 15875 (sART) had 14763 NCS and the majority of families had had an ART pregnancy first followed by natural conception (Supplementary table S3).

The majority of the final cohort were ethnically white and singleton births, and a greater proportion of the ART and NCS groups were from a higher socioeconomic class. The majority of women who underwent ART were aged between 30-39 years at the time of delivery, while those with NC births tended to be slightly younger (25-39 years).

For peer review only

Table 1: Demographic characteristics of ART children, naturally conceived siblings, and matched naturally conceived population controls born between 1st April 1992 & 31st July 2009.

	HFEA-ONS cohort			
	**ART**	**Control (NCP)**	**ART with siblings (sART)**	**Siblings (NCS)**
**Infants**	75348	164823	15875	14763
**Mean BW (S.D)**				
Singleton	3282.0 (620.3)	3353.83 (578.5)	3311.12 (605.1)	3430.05 (581.9)
Multiple	2378.3 (593.4)	2340.28 (626.1)	2405.10 (608.1)	2292.03 (780.5)
**Sex**				
Female	36994 (49.1 %)	80859 (49.1 %)	7598 (47.9 %)	7271 (49.3 %)
Male	38354 (50.9 %)	83964 (50.9 %)	8277 (52.1 %)	7492 (50.8 %)
**Multiplicity/plurality**				
Singleton	44488 (59.1 %)	99554 (60.4 %)	10592 (66.7 %)	14055 (95.2 %)
Twin	30860 (40.9 %)	65269 (39.6 %)	5283 (33.3 %)	708 (4.8 %)
**Maternal age group**				
<25	731 (0.1 %)	33156 (20.1 %)	256 (1.6 %)	388 (2.6 %)
25-29	5315 (7.1 %)	30231 (18.3 %)	1326 (8.4 %)	1032 (6.1 %)
30-34	23342 (30.1 %)	46050 (27.9 %)	5466 (34.4 %)	3855 (26.1 %)
35-39	29187 (38.7 %)	30345 (18.4 %)	5872 (36.1 %)	5711 (38.7 %)
40-44	8705 (11.6 %)	7812 (4.7 %)	1315 (8.3 %)	2157 (14.6 %)
>=45	84 (0.1 %)	587 (0.4 %)	23 (0.1 %)	53 (0.4 %)
Missing	7984 (10.6 %)	16642 (10.1 %)	1617 (10.2 %)	1567 (10.6 %)
**Birth year**				
1992	57 (0.1 %)	131 (0.1 %)	12 (0.08%)	23 (0.2 %)
1993	1595 (2.1 %)	3187 (1.9 %)	299 (1.8%)	337 (2.3 %)
1994	2021 (2.7 %)	4029 (2.4 %)	425 (2.7 %)	389 (2.6 %)
1995	2439 (3.2 %)	4911 (2.1 %)	518 (3.3 %)	526 (3.6 %)
1996	2977 (3.1 %)	5989 (3.6 %)	641 (4.0 %)	577 (3.9 %)
1997	3574 (4.7 %)	7172 (4.4 %)	776 (4.9 %)	679 (4.6 %)
1998	3826 (5.1 %)	7670 (4.7 %)	861 (5.4 %)	800 (5.4 %)
1999	4253 (5.6 %)	8531 (5.2 %)	1023 (6.4 %)	804 (5.5 %)
2000	4504 (5.1 %)	9106 (5.5 %)	1119 (7.1 %)	961 (6.5 %)
2001	4754 (6.3 %)	9677 (5.9 %)	1163 (7.3 %)	991 (6.7 %)
2002	5149 (6.8 %)	10464 (6.4 %)	1339 (8.4 %)	999 (6.8 %)
2003	5531 (7.3 %)	11373 (6.9 %)	1401 (8.8 %)	1064 (7.2 %)
2004	5671 (7.5 %)	11835 (7.2 %)	1379 (8.7 %)	1098 (7.4 %)
2005	5768 (7.7 %)	12310 (7.5 %)	1372 (8.6 %)	1109 (7.5 %)
2006	6331 (8.4 %)	14248 (8.6 %)	1273 (8.0 %)	1116 (7.6 %)
2007	6410 (8.5 %)	15976 (9.7 %)	1126 (7.1 %)	1209 (8.9 %)
2008	6395 (8.5 %)	17222 (10.5 %)	719 (4.5 %)	1271 (8.6 %)
2009	4092 (5.4 %)	10992 (6.7 %)	429 (2.7 %)	810 (5.5 %)

HFEA- Human Fertilization & Embryology Authority; ONS- Office for National Statistics; NCP: naturally conceived population controls; NCS- naturally conceived siblings; sART- ART with siblings; BW - birthweight; S.D - standard deviation

For peer review only

Table 2: Fertility characteristics of ART children by cohort

	HFEA-ONS cohort		HFEA-ONS-HES sub-cohort	
	ART	sART (ART with siblings)	HES-ART	HES-sART (ART with siblings)
**Multiplicity/plurality**				
Singletons	44489 (59.0 %)	10592 (66.7 %)	37891 (59.4 %)	8383 (67.1 %)
Twins	30859 (40.1 %)	5283 (33.3 %)	25986 (40.6 %)	3946 (32.0 %)
**Paternal age at childbirth**				
<25	406 (0.5 %)	122 (0.8 %)	324 (0.5 %)	87 (0.7 %)
25-29	4878 (6.5 %)	1206 (7.6 %)	3911 (6.1 %)	938 (7.6 %)
30-34	21526 (28.6 %)	5304 (33.4 %)	17706 (27.7 %)	4116 (33.4 %)
35-39	27678 (36.7 %)	6007 (37.8 %)	23626 (36.1 %)	4692 (38.1 %)
40-44	13458 (17.9 %)	2306 (14.5 %)	11715 (18.3 %)	1778 (14.4 %)
45-49	4551 (6.0 %)	604 (3.8 %)	4010 (6.3 %)	468 (3.8 %)
50-54	1636 (2.2 %)	190 (1.2 %)	1440 (2.3 %)	146 (1.2 %)
>=55	848 (1.1 %)	82 (0.5 %)	787 (1.2 %)	65 (0.5 %)
Missing	367 (0.5 %)	54 (0.4 %)	348 (0.6 %)	39 (0.3 %)
**Type of ART**				
ICSI	26629 (38.6 %)	4409 (30.1 %)	26210 (44.6 %)	3995 (34.1 %)
IVF	42096 (61.0 %)	10170 (69.4 %)	32282 (54.1 %)	7364 (64.4 %
IVF:ICSI	248 (0.4 %)	73 (0.5 %)	247 (0.4 %)	72 (0.6 %)
**Cause of infertility**				
Male factor	29203 (38.8 %)	5675 (35.8 %)	24876 (38.1 %)	4198 (34.1 %)
Endometriosis	2225 (2.1 %)	495 (3.1 %)	1962 (3.1 %)	401 (3.3 %)
Ovulatory	8581 (11.4 %)	2198 (13.9 %)	7713 (12.1 %)	1854 (15.1 %)
Tubal	19915 (26.4 %)	3450 (21.7 %)	15041 (23.6 %)	2378 (19.3 %)
Unknown cause	15387 (20.4 %)	4048 (25.5 %)	14252 (22.3 %)	3491 (28.3 %)
**Fresh/Frozen transfer**				
Fresh	66534 (88.3 %)	14079 (88.7 %)	56230 (88.0 %)	10907 (88.5 %)
Frozen	8739 (11.6 %)	1782 (11.2 %)	7595 (11.9 %)	1414 (11.7%)
Missing	75 (0.1 %)	14 (0.1 %)	52 (0.1 %)	8 (0.1 %)
**Previous live births**				
0	67235 (89.2 %)	14878 (93.7 %)	56250 (88.1 %)	11498 (93.3 %)
1	7829 (10.4 %)	958 (6.1 %)	7348 (11.5 %)	795 (6.5 %)
2	273 (0.4 %)	35 (0.2 %)	268 (0.4 %)	32 (0.3 %)
3	9 (0.0 %)	2 (0.0 %)	9 (0.1 %)	2 (0.0 %)
Missing	2 (0.0 %)	2 (0.0 %)	2 (0.0 %)	2 (0.0 %)
**Parity of mother (previous pregnancies as recorded on the register)**				
0	64502 (85.6 %)	14270 (89.9 %)	53987 (84.5 %)	11029 (89.5 %)
1	9798 (13.0 %)	1450 (9.2 %)	8901 (13.9 %)	1167 (9.5 %)
2	949 (1.3 %)	140 (0.9 %)	894 (1.4 %)	119 (0.1 %)
3	83 (0.1 %)	9 (0.1 %)	79 (0.1 %)	8 (0.1 %)
4	14 (0.0 %)	4 (0.0 %)	14 (0.0 %)	4 (0.0 %)
Null	2 (0.0 %)	2 (0.0 %)	2 (0.0 %)	2 (0.0 %)
**Infertility duration in years**				
Mean (SD)	4.78 (2.9)	4.37 (2.6)	4.62 (2.8)	4.31 (2.6)
Missing	17451 (23.2 %)	4951 (31.2 %)	17898 (24.9 %)	5364 (27.1 %)

HFEA- Human Fertilization & Embryology Authority; ONS- Office for National Statistics; HES- Hospital Episode Statistics; sART- ART with siblings; ICSI - intracytoplasmic sperm injection; IVF - in vitro fertilisation

For peer review only

Table 3: Demographic characteristics of ART children, naturally conceived siblings, and matched naturally conceived population controls born between 1st April 1997 and 31st July 2009.

	HFEA-ONS-HES sub-cohort			
	**HES-ART**	**Control (HES-NCP)**	**ART with siblings (HES-sART)**	**Siblings (HES-NCS)**
**Infants**	63877	127544	12329	11343
**Follow-up period in days**				
**Median (IQR)**	4429 (2181)	4409 (2141)	4635 (1846)	4307 (2029)
**Mean BW (S.D)**				
Singleton<br>Multiple	3166.95 (742.3)<br>2172.27 (715.4)	3271.67 (648.5)<br>2155.77 (683.9)	3222.25 (699.3)<br>2201.58 (724.9)	3346.54 (712.0)<br>2301.17 (677.8)
**Sex**				
Female	31435 (49.2 %)	62785 (49.2 %)	5907 (47.9 %)	5573 (49.1 %)
Male	32442 (50.8 %)	64759 (50.8 %)	6422 (52.1 %)	5770 (50.9 %)
**Multiplicity/plurality**				
Singleton	37890 (59.4 %)	75642 (59.3 %)	8383 (68.0 %)	10815 (95.8 %)
Twin	25987 (40.6 %)	51902 (40.7 %)	3946 (31.1 %)	528 (4.2 %)
**IMD decile at earliest**				
1 (most deprived)	2045 (3.2 %)	12696 (9.9 %)	349 (2.8 %)	299 (2.6 %)
2	2650 (4.2 %)	11104 (8.7 %)	458 (3.7 %)	396 (3.5 %)
3	3311 (5.2 %)	10057 (7.8 %)	539 (4.4 %)	463 (4.1 %)
4	3926 (6.1 %)	9550 (7.5 %)	678 (5.4 %)	588 (5.2 %)
5	4666 (7.2 %)	9161 (7.5 %)	862 (6.1 %)	746 (6.6 %)
6	5350 (8.4 %)	8872 (6.5 %)	1084 (8.8 %)	921 (8.1 %)
7	6142 (9.6 %)	8842 (6.9 %)	1205 (9.8 %)	1044 (9.2 %)
8	6675 (10.5 %)	8925 (7.0 %)	1299 (10.5 %)	1161 (10.3 %)
9	7729 (12.1 %)	9083 (7.1 %)	1557 (12.6 %)	1386 (12.2 %)
10 (least deprived)	7710 (12.1 %)	8299 (6.5 %)	1720 (14.0 %)	1499 (13.3 %)
Missing	13673 (21.4 %)	30955 (24.3 %)	2580 (20.9 %)	2840 (25.0 %)
**Birth year**				
1997	2597 (4.1 %)	5160 (4.1 %)	518 (4.2 %)	242 (2.1 %)
1998	3708 (5.8 %)	7389 (5.9 %)	743 (6.0 %)	419 (3.7 %)
1999	4083 (6.4 %)	8126 (6.5 %)	852 (6.9 %)	560 (4.9 %)
2000	4310 (6.8 %)	8633 (6.9 %)	925 (7.5 %)	781 (6.9 %)
2001	4559 (7.1 %)	9160 (7.3 %)	972 (7.9 %)	879 (7.78 %)
2002	4980 (7.8 %)	9933 (7.9 %)	1171 (9.5 %)	937 (8.3 %)
2003	5379 (8.4 %)	10788 (8.5 %)	1253 (10.2 %)	1012 (8.9 %)
2004	5561 (8.7 %)	11082 (8.7 %)	1271 (10.3 %)	1067 (9.4 %)
2005	5662 (8.9 %)	11326 (8.8 %)	1271 (10.3 %)	1078 (9.5 %)

Final HFEA-ONS-HES sub-cohort

All births before 1st April 1997 were excluded for the purpose of linkage to HES, resulting in 63877, 11343 and 127544 children in the HES-ART, HES-NCS, and HES-NCP groups, respectively. Linkage 2 (Figure 2) had a success rate of 84.7%, and the descriptive characteristics of the sub-cohort produced have been shown in Tables 2 & 3

2006	6275 (9.8 %)	12513 (9.7 %)	1217 (9.7 %)	1100 (9.7 %)
2007	6342 (9.9 %)	12701 (9.8 %)	1058 (8.6 %)	1199 (10.6 %)
2008	6347 (9.9 %)	12718 (9.8 %)	670 (5.4 %)	1260 (11.1 %)
2009	4074 (6.4 %)	8015 (6.2 %)	408 (3.3 %)	809 (7.1 %)
**Ethnicity**				
White	61921 (96.9 %)	122050 (95.7 %)	11983 (97.2 %)	11084 (97.7 %)
Asian/Asian British	959 (1.5 %)	2496 (1.1 %)	197 (1.6 %)	153 (1.4 %)
Chinese	35 (0.1 %)	89 (0.1 %)	8 (0.1 %)	1 (0.0 %)
Black/African/Caribbean/Black	433 (0.7 %)	5268 (4.0 %)	61 (0.5 %)	39 (0.3 %)
Mixed/Multiple ethnic groups	318 (0.5 %)	721 (0.6 %)	38 (0.3 %)	27 (0.2 %)
Other ethnic group	211 (0.3 %)	498 (0.4 %)	42 (0.3 %)	39 (0.3 %)
**Maternal age at delivery**				
<=25	710 (1.1 %)	27783 (21.9 %)	241 (1.1 %)	317 (2.9 %)
25-29	5085 (7.1 %)	25115 (19.7 %)	1209 (9.9 %)	887 (7.8 %)
30-34	21994 (34.4 %)	38896 (30.5 %)	4797 (38.9 %)	3309 (29.1 %)
35-39	27682 (43.4 %)	25907 (20.3 %)	4998 (40.6 %)	4954 (43.7 %)
40-44	8164 (12.1 %)	6419 (5.2 %)	1057 (8.6 %)	1830 (16.2 %)
>=45	217 (0.3 %)	772 (0.6 %)	20 (0.2 %)	45 (0.4 %)
Missing	25 (0.0 %)	2652 (2.0 %)	7 (0.0 %)	1 (0.0 %)

HFEA- Human Fertilization & Embryology Authority; ONS- Office for National Statistics; HES- Hospital Episode Statistics; sART- ART with siblings; ICSI - intracytoplasmic sperm injection; IVF - in vitro fertilisation

Exemplar analysis: Singleton BW

The exemplar analysis included all singleton births in the HFEA-ONS cohort and HFEA-ONS-HES sub-cohort.

HFEA-ONS: Comparing ART to NCP showed that children born after fresh embryo transfers were lighter (BW difference: -131g; 95% CI: -140, -123) and those born after frozen embryo transfers were heavier (BW difference: 35g; 95% CI: 19, 52) than the NC controls (Table 4; Supplementary table S4). Moreover, children born after frozen embryo transfers were significantly heavier than those born after fresh embryo transfers (BW difference: -167g; 95% CI: -183, -150).
Family-matched analysis showed that children born after fresh embryo transfers were lighter (BW difference: -54g; 95% CI: -72, -36) and those born after frozen embryo transfers (BW difference: 138g; 95% CI: 101, 175) were heavier than the NCS group (Table 4; Supplementary table S5). Moreover, children born after frozen embryo transfers were significantly heavier than those born after fresh embryo transfers (BW difference =-193g; 95% CI: -232, -154).

HFEA-ONS-HES: Comparing HES-ART to HES-NCP showed that children born after fresh embryo transfers were lighter (BW difference: -152g; 95% CI: -162, -142) and those born after frozen embryo transfers were similar in weight (BW difference: 3g; 95% CI: -17, 22) to the NC controls (Table 4;

For peer review only

Supplementary table S6). Moreover, children born after frozen embryo transfers were significantly heavier than those born after fresh embryo transfers (BW difference:-155g; 95% CI: -175, -135). Family-matched analysis comparing showed that children born after fresh embryo transfers were lighter (BW difference: -57g; 95% CI: -75, -38) and those born after frozen embryo transfers were heavier (BW difference: 152g; 95% CI: 113, 190) than the HES-NCS group (Table 4; Supplementary table S7). Moreover, children born after frozen embryo transfers were significantly heavier than those born after fresh embryo transfers (BW difference: -209g; 95% CI: -249, -168).

Table 4: Statistical analysis of BW

	**HFEA-ONS cohort** *BW coefficient (95%CI)*		**HFEA-ONS-HES sub-cohort** *BW coefficient (95%CI)*	
	**ART (N=75348) – NCP (N=164823)** * adjusted for maternal age at delivery and sex	**sART (N=15875) NCS (N=14763)** *adjusted for maternal age at delivery, sex, and order of pregnancy.	**HES-ART (N=63877) HES-NCP (N=127544)** *adjusted for maternal age at delivery, sex, IMD at earliest appointment, and ethnicity.	**HES-sART (N=12329) – HES-NCS (N=11343)** *adjusted for maternal age at delivery, sex, and order of pregnancy.
Fresh vs NC (g)	-131 (-140, -123)	-54 (-72, -36)	-152 (-162, -142)	-57 (-75, -38)
Frozen vs NC (g)	35 (19, 52)	138 (101, 175)	3 (-17, 22)	152 (113, 190)
Fresh vs Frozen (g)	-167 (-183, -150)	-193 (-232, -154)	-155 (-175, -135)	-209 (-249, -168)

HFEA- Human Fertilization & Embryology Authority; ONS- Office for National Statistics; HES- Hospital Episode Statistics; sART- ART with siblings; NCP- Naturally conceived population control; NCS- Naturally conceived siblings; NC- naturally conceived; BW – birthweight; CI- confidence interval.

DISCUSSION

This study aimed to substantially enhance the research value of the HFEA register by utilising electronic record linkage methodology to establish a cohort of ART children born in the UK between

For peer review only

1992 and 2009, their NCS and matched NCP controls. Additionally, a sub-cohort consisting of those born between 1997 and 2009 was also linked to the Hospital Episode Statistics database to allow examination of postnatal health outcomes. The final cohort consisted of 75348 children born after non-donor ART carried out in the UK between 1st April 1992 and 31st July 2009, 14763 NCS and 164823 matched NCP controls. Of these, 63877 ART, 11343 NCS, and 127544 matched NCP controls were linked to hospital data up to 2015, thus providing a valuable resource for comprehensive, non-invasive, continued immediate and longer term health monitoring of this population as they grow up.

An exemplar analysis comparing BW between children born after ART, their NCS and matched NCP controls was also carried out to demonstrate the validity of this cohort. The results of this analysis confirmed the findings of our previous linkage study on a subset of the entire 1992-2009 cohort (39), that children born after fresh embryo transfer tended to be lighter and those born from frozen transfer were heavier compared to their NCS and matched NCP controls. These findings are broadly in agreement with numerous large register-based studies and meta-analyses, but add valuable confirmation of the magnitude of BW differences in a large UK population (15, 57-65). Castillo et al (66) reported a similar magnitude of difference to that seen in sibling pairs in a more recent UK cohort, suggesting that the difference in BW between the fresh and frozen embryo transfer groups may not be explained entirely by maternal characteristics and the underlying cause of infertility as these were likely to have remained relatively stable between siblings. Notably, the BW difference observed between frozen transfer and NC babies is much larger in the sibling pair analysis than in the non-sibling comparison, and larger than observed by Hann et al (39). This merits further investigation, especially as increased rates of macrosomia following frozen transfer are an ongoing concern (34, 62). Other possible explanations include differences in the maternal uterine environment caused by either hormonal stimulation (fresh cycles) or hormonal preparation of the uterus (frozen cycles) (67). Although lack of appropriate data on the HFEA register prevented exploration of these effects in the current analysis, a recent UK cohort study by Castillo et al using more detailed clinic data reported no association between type of FET and birthweight (66). Molecular changes in the early embryo during freezing and thawing might also result in changes in the developmental process leading to altered BW and long term health (33, 68-71).

In the current study, the difference in BW between children born after fresh embryo transfer and those born after natural conception was seen to attenuate markedly from -131g in the population to -54g in the within-sibling analysis. By contrast, the difference in BW between children born after frozen embryo transfer and those born after natural conception was seen to markedly increase from 35g in the population to 138g in the within sibling analysis in the same dataset. This suggests

For peer review only - http://bmjopen.bmj.com/site/about/guidelines.xhtml

that the findings of the within-family analysis must be interpreted with caution, with potential explanatory factors for these differences including subfertility and familial masking confounders in relation to the increase of the association of frozen transfer vs natural conception. Unavailability of birth order data for the population controls prevented us from adjusting for it in the ART vs NCP comparison, and this could plausibly act as a strong confounder given its association with mean BW is stronger than any differences due to ART in most studies. This notable difference between the population and within-family analyses requires further exploration.

Repeating the exemplar analysis using the cohort and sub-cohort produced by the linkages described above not only increased confidence in the findings but also allowed us to control for potential confounding factors such as maternal age, birth order, socioeconomic status and ethnicity which are known to affect BW (72).

The cohort described here will allow more effective use of HFEA data for health monitoring, and will be of value to researchers from a variety of professional backgrounds. The linked dataset will be returned to the HFEA and access will be controlled by them, although specific ethical approval will be required along with that of the Confidentiality Advisory Group (CAG) of the Health Research Authority (HRA).

In order to provide reliable risk estimates of relatively rare conditions in a specific group of children born after ART, large, well-designed cohort studies, preferably including families, are necessary. However, as it may not always be appropriate to contact these families directly due to the very personal nature of the treatments involved, access to national-level electronically linked data on cohorts such as the current one provides a cost-effective, non-intrusive, and comprehensive way to answer sophisticated research questions in a wide range of areas.

STRENGTHS AND LIMITATIONS

The main strength of this study lies in the meticulous linkage of robust, routinely collected administrative health data to yield a large cohort that is nationally unique and complements other similar linked cohorts, such as the Nordic linked cohorts (19), thus increasing the generalisability, accuracy, and precision of results from subsequent analyses. The research value and quality and consistency of the HFEA cohort 1992-2009 data has been considerably enhanced through linkage to the ONS birth records and HES databases and removal of artefacts and duplicates. Linkage to the hospital admissions and outpatient database provides long-term mortality and morbidity outcome data on offspring, and also represents a high-quality cross-sectoral evidence base that can be used for longitudinal research, policy planning and strategic development.

For peer review only

However, there are also several challenges associated with electronic data linkage studies in general, and Harron et al. (73) summarised these into three groups, namely those pertaining to the (i) the data linkage environment and privacy preservation; (ii) the linkage process itself, which includes data preparation linkage methods; and (iii) the linkage quality and potential bias in linked data. In the context of the current study, limitations in the linkage process itself included those associated with the method of definition of NC siblings used. The identification of sibling controls would be very sensitive to any errors in linkage, and missed second ART babies would appear as conventional siblings. Parents who had NC children were likely to have been less severely sub-fertile than parents who did not, and ART children born to the truly infertile would not have conventional siblings. Therefore, the NCS group in this study would have parents with borderline fertility problems or those who developed secondary infertility after the birth of the NC child. For this reason, extensive quality assurance procedures were carried out on the linkage process. With regard to the linkage quality, as this was a bespoke linkage that has never been carried out before, there were no standardized quality assurance measures in place; however, the linkage was extensively checked manually by the team at NHS Digital prior to dissemination for analysis.

The often weak/inaccurate identifier data on the HFEA register and the high threshold for matching used in the current study meant that approximately 23% of children were lost during the linkage process. This is a limitation as almost 100% coverage can be achieved in settings with availability of unique identifying numbers for both mother and child. However, although unavailable for the study period explored here, the HFEA now record both the mother and child's NHS number, suggesting that this loss to follow-up may be avoided in future studies (74). As HES monitoring data is only available from 1997, children born to women who underwent ART prior to this could not be linked to any hospital records, thus limiting our ability to examine health outcomes in those born before 1997 and exploring the effects of changes in ART techniques on health over that period. However, comparison of maternal age between the unlinked and linked records showed no substantive differences (Supplementary table S8). Unfortunately, unavailability of data prevented us from carrying out further examination and comparison of the educational levels and socioeconomic profile of unlinked records. Nevertheless, linkage of cases born before 1997 to their birth records is still valuable as it may allow investigation of outcomes available on the birth register in the future. Moreover, although these data have been used extensively for research purposes, there have been long-standing concerns regarding the quality, completeness and coverage of HES records within health services and the academic community (75).

For peer review only

The BW analysis provides an example use of these cohorts and the strengths and limitations of sibling comparisons. However, it cannot be considered as an exhaustive analysis as gestational data was not available for NC children and there is limited covariate data in the present dataset. This is mainly because gestation is not recorded on the birth registration dataset and is of poor quality on the maternal arm of a child's HES record (>50% missing). As a result, although the quality of such data for the ART group (accessed from the HFEA register) was good, it was not available for the NCS and NCP control groups. Further ART treatment data is available on the HFEA register which was not included in the current datasets, and these could be extracted for future studies. In particular the developmental stage of the embryo at transfer and number of embryos transferred are important determinants of treatment success rate (i.e. the probability of a child appearing on the HFEA birth register) and also gestation and BW (37, 39, 66). Unfortunately, there was no way to systematically identify and account for children born after ART who emigrated or children born to mothers who lived in the England & Wales but travelled abroad for ART treatment, although European estimates of cross-border reproductive care activity suggests that the number of children to whom either of these applies is be small (76). Due to lack of maternal data, it was not feasible to identify and exclude NCP controls that were part of a triplet or higher order birth. However, the number of such individuals is estimated to be small as the incidence of naturally occurring triplets is in the range of 1:5000-1:9000 (44).

Lastly, an important limitation of the current cohort is that it finishes in 2009. Given that ART technologies have advanced rapidly in the last 10 years, there is an urgent need to continue to prospectively monitor the next cohort of children from 2010 onwards. Two recent UK studies (57, 66) have suggested that BWs from both fresh and frozen transfers are increasing with time and may be different in the post 2010 cohort, suggesting that there may potentially be differences (of unknown origin) in the health outcomes of babies born more recently.

COLLABORATION

The current study carried out a bespoke record linkage that generated a new child cohort for use in exploring the relationship between conception via ART and short- and long-term health outcomes in offspring and enabling future linkage studies to other developmental and health data sources.

Upon completion of our further planned analyses, access to the linked dataset will be controlled by HFEA and NHS Digital and specific ethical approval will be required along with approval from the Confidentiality Advisory Group (CAG) of the Health Research Authority (HRA).

For peer review only

FURTHER DETAILS

Contributors: In line with the ICMJE authorship guidelines, authors AGS, SAR, DRB, SMN, BL, and DAL made substantial contributions to the conception or design of the work. Authors AGS and MP made substantial contributions to the acquisition of data. Author MP undertook data preparation and provided statistical analysis, with SAR and JG providing statistical oversight. Authors MP, SAR, JG, DRB, SMN, DAL, BL, and AGS made substantial contributions to the interpretation of data. All authors contributed to the drafting of the manuscript, and/or the revising of the manuscript. All authors have given final approval of the version to be published, and agree to its accuracy.

Funding: This work was supported by the Medical Research Council [Grant number MR/L020335/1].

Competing interest: None declared.

Ethical considerations: Ethical approval and Section 251 support were obtained from the NHS Research Ethics Committee and Confidentiality Advisory Group, respectively. Additional data access permissions were sought from the HFEA Register Research Panel, ONS Micro-Data Release board, and the NHS Digital Medical Register. All researchers with data access underwent NHS Digital Data Security Awareness and ONS Safe Researcher accreditation.

Provenance and peer review: Not commissioned; externally peer reviewed.

Data sharing statement: De-identified linked cohort data can be accessed from the Human Fertilisation and Embryology Authority and NHS Digital where it will be held with restricted access. Specific ethical approval from the Research Ethics Committee (REC) and the Confidentiality Advisory Group (CAG) of the Health Research Authority (HRA) will be required for access.

For peer review only

REFERENCES

(1) Lancaster P, de Mouzon J. Global Committee Reproductive ART Surveillance: Monitoring Technologies Assisted The (ICMART) International. *Assisted Reproductive Technology Surveillance*. 2019:101.
2) Barker DJ. Fetal programming of coronary heart disease. *Trends in Endocrinology & Metabolism*. 2002;13(9):364-8.
(3) Lane M, Robker RL, Robertson SA. Parenting from before conception. *Science*. 2014;345(6198):756-60.
(4) Fleming TP, Watkins AJ, Velazquez MA, Mathers JC, Prentice AM, Stephenson J, et al. Origins of lifetime health around the time of conception: causes and consequences. *Lancet*. 2018;391(10132):1842-52.
(5) Fisher-Jeffes L, Banerjee I, Sutcliffe A. Parents' concerns regarding their ART children. *Reproduction*. 2006;131(2):389-94.
(6) Källén B, Finnström O, Nygren KG, Olausson PO. In vitro fertilization in Sweden: child morbidity including cancer risk. *Fertility and Sterility*. 2005;84(3):605-10.
(7) Schieve LA, Rasmussen SA, Buck GM, Schendel DE, Reynolds MA, Wright VC. Are children born after assisted reproductive technology at increased risk for adverse health outcomes? *Obstetrics Gynecology*. 2004;103(6):1154-63.
(8) Reddy UM, Wapner RJ, Rebar RW, Tasca RJ. Infertility, assisted reproductive technology, and adverse pregnancy outcomes: executive summary of a National Institute of Child Health and Human Development workshop. *Obstetrics Gynecology*. 2007;109(4):967-77.
(9) Pandey S, Shetty A, Hamilton M, Bhattacharya S, Maheshwari A. Obstetric and perinatal outcomes in singleton pregnancies resulting from IVF/ICSI: a systematic review and meta-analysis. *Human Reproduction Update*. 2012;18(5):485-503.
(10) Gnoth C, Godehardt E, Frank-Herrmann P, Friol K, Tigges J, Freundl G. Definition and prevalence of subfertility and infertility. *Human Reproduction*. 2005;20(5):1144-7.
(11) Ceelen M, van Weissenbruch MM, Prein J, Smit JJ, Vermeiden JP, Spreeuwenberg M, et al. Growth during infancy and early childhood in relation to blood pressure and body fat measures at age 8-18 years of IVF children and spontaneously conceived controls born to subfertile parents. *Human Reproduction*. 2009;24(11):2788-95.
(12) Ceelen M, van Weissenbruch MM, Vermeiden JP, van Leeuwen FE, Delemarre-van de Waal HA. Pubertal development in children and adolescents born after IVF and spontaneous conception. *Human Reproduction*. 2008;23(12):2791-8.
(13) Dumoulin JC, Land JA, Van Montfoort AP, Nelissen EC, Coonen E, Derhaag JG, et al. Effect of in vitro culture of human embryos on birthweight of newborns. *Human Reproduction*. 2010;25(3):605-12.
(14) Van Montfoort AP, Hanssen LL, de Sutter P, Viville S, Geraedts JP, de Boer P. Assisted reproduction treatment and epigenetic inheritance. *Human Reproduction Update*. 2012;18(2):171-97.
(15) Stormlund S, Sopa N, Zedeler A, Bogstad J, Prætorius L, Nielsen HS, et al. Freeze-all versus fresh blastocyst transfer strategy during in vitro fertilisation in women with regular menstrual cycles: multicentre randomised controlled trial. *British Medical Journal*. 2020;370:m2519.
(16) Sandin S, Nygren KG, Iliadou A, Hultman CM, Reichenberg A. Autism and Mental Retardation Among Offspring Born After In Vitro Fertilization. *Journal of the American Medical Association*. 2013;310(1):75-84.
(17) Bay B, Mortensen EL, Hvidtjørn D, Kesmodel US. Fertility treatment and risk of childhood and adolescent mental disorders: register based cohort study. *British Medical Journal*. 2013;347:f3978.
(18) Davies MJ, Moore VM, Willson KJ, Van Essen P, Priest K, Scott H, et al. Reproductive Technologies and the Risk of Birth Defects. *New England Journal of Medicine*. 2012;366(19):1803-13.

For peer review only

(19) Opdahl S, Henningsen A-KA, Bergh C, Gissler M, Romundstad LB, Petzold M, et al. Data Resource Profile: Committee of Nordic Assisted Reproductive Technology and Safety (CoNARTaS) cohort. *International Journal of Epidemiology*. 2019;49(2):365-6f.
(20) Henningsen AK, Romundstad LB, Gissler M, Nygren K-G, Lidegaard Ø, Skjaerven R, et al. Infant and maternal health monitoring using a combined Nordic database on ART and safety. *Acta Obstetricia et Gynecologica Scandinavica*. 2011;90(7):683-91.
(21) Wennberg AL, Opdahl S, Bergh C, Henningsen A-KA, Gissler M, Romundstad LB, et al. Effect of maternal age on maternal and neonatal outcomes after assisted reproductive technology. *Fertility and sterility*. 2016;106(5):1142-9. e14.
(22) Finnström O, Källén B, Lindam A, Nilsson E, Nygren KG, Olausson PO. Maternal and child outcome after in vitro fertilization--a review of 25 years of population-based data from Sweden. *Acta Obstetricia et Gynecologica Scandinavica*. 2011;90(5):494-500.
(23) Henningsen AK, Pinborg A, Lidegaard Ø, Vestergaard C, Forman JL, Andersen AN. Perinatal outcome of singleton siblings born after assisted reproductive technology and spontaneous conception: Danish national sibling-cohort study. *Fertility and Sterility*. 2011;95(3):959-63.
(24) Kalra SK, Ratcliffe SJ, Barnhart KT, Coutifaris C. Extended embryo culture and an increased risk of preterm delivery. *Obstetrics Gynecology*. 2012;120(1):69-75.
(25) Romundstad LB, Romundstad PR, Sunde A, von Düring V, Skjaerven R, Gunnell D, et al. Effects of technology or maternal factors on perinatal outcome after assisted fertilisation: a population-based cohort study. *Lancet*. 2008;372(9640):737-43.
(26) Lawlor DA, Tilling K, Davey Smith G. Triangulation in aetiological epidemiology. *International Journal of Epidemiology*. 2017;45(6):1866-86.
(27) Romundstad LB, Romundstad PR, Sunde A, von Düring V, Skjaerven R, Vatten LJ. Increased risk of placenta previa in pregnancies following IVF/ICSI; a comparison of ART and non-ART pregnancies in the same mother. *Human Reproduction*. 2006;21(9):2353-8.
(28) Human Fertilisation and Embryology Authority. *Fertility treatment 2014–2016 Trends and figures*. London: HFEA. 2018. URL: https://www.hfea.gov.uk/media/3188/hfea-fertility-trends-and-figures-2014-2016.pdf
(29) *Human Fertilisation and Embryology Act 1990* (UK). URL: https://www.legislation.gov.uk/ukpga/1990/37/contents
(30) *Human Fertilisation and Embryology Privacy Policy*. 2018. (UK) URL: https://www.hfea.gov.uk/privacy-policy-cookies-and-accessibility/privacy-policy/
(31) *Human Fertilisation & Embryology Authority* (2019). URL: https://www.hfea.gov.uk/.
(32) Templeton A, Morris JK, Parslow W. Factors that affect outcome of in-vitro fertilisation treatment. *Lancet*. 1996;348(9039):1402-6.
(33) Nelson SM, Lawlor DA. Predicting Live Birth, Preterm Delivery, and Low Birth Weight in Infants Born from In Vitro Fertilisation: A Prospective Study of 144,018 Treatment Cycles. *PLOS Medicine*. 2011;8(1):e1000386.
(34) Maheshwari A, Raja EA, Bhattacharya S. Obstetric and perinatal outcomes after either fresh or thawed frozen embryo transfer: an analysis of 112,432 singleton pregnancies recorded in the Human Fertilisation and Embryology Authority anonymized dataset. *Fertility and Sterility*. 2016;106(7):1703-8.
(35) Kamath MS, Antonisamy B, Selliah HY, Sunkara SK. Perinatal outcomes of singleton live births with and without vanishing twin following transfer of multiple embryos: analysis of 113 784 singleton live births. *Human Reproduction*. 2018;33(11):2018-22.
(36) Marconi N, Raja EA, Bhattacharya S, Maheshwari A. Perinatal outcomes in singleton live births after fresh blastocyst-stage embryo transfer: a retrospective analysis of 67 147 IVF/ICSI cycles. *Human Reproduction*. 2019;34(9):1716-25.
(37) Castillo CM, Johnstone ED, Horne G, Falconer DA, Troup SA, Cutting R, et al. Associations of IVF singleton birthweight and gestation with clinical treatment and laboratory factors: a multicentre cohort study. *Human Reproduction*. 2020;35(12):2860-70.

For peer review only

(38) Roberts S, McGowan L, Hirst W, Brison D, Vail A, Lieberman B. Towards single embryo transfer? Modelling clinical outcomes of potential treatment choices using multiple data sources: predictive models and patient perspectives. *Health Technology Assessment*. 2010;14(38):1-237.
(39) Hann M, Roberts SA, D'Souza SW, Clayton P, Macklon N, Brison DR. The growth of assisted reproductive treatment-conceived children from birth to 5 years: a national cohort study. BMC Medicine. 2018;16(1):224.
(40) *Human Fertilisation & Embryology Act 2008*. (UK). URL: https://www.legislation.gov.uk/ukpga/2008/22/contents
(41) Williams CL, Bunch KJ, Stiller CA, Murphy MFG, Botting BJ, Wallace WH, et al. Cancer Risk among Children Born after Assisted Conception. *New England Journal of Medicine*. 2013;369(19):1819-27.
(42) Human Fertilisation and Embryology Authority. *Fertility treatment 2018: trends and figures.* URL: https://www.hfea.gov.uk/about-us/publications/research-and-data/fertility-treatment-2018-trends-and figures/#:~:text=Birth%20rates%20from%20IVF%20have,HFEA%20was%20established%20in%201991.
(43) Human Fertilisation and Embryology Authority. *Fertility treatment 2018: trends and figures Quality and Methodology Report.* URL: https://www.hfea.gov.uk/about-us/publications/research-and-data/fertility-treatment-2018-trends-and-figures/fertility-treatment-2018-quality-and-methodology-report/
(44) Office for National Statistics. *User guide to birth statistics.*2019. URL: https://www.ons.gov.uk/peoplepopulationandcommunity/birthsdeathsandmarriages/livebirths/methodologies/userguidetobirthstatistics
(45) NHS Digital. *Hospital Episode Statistics*. URL: https://digital.nhs.uk/data-and-information/data-tools-and-services/data-services/hospital-episode-statistics.
(46) Great Britain. National Audit Office, Great Britain. Parliament. House of Commons. Healthcare across the UK: A comparison of the NHS in England, Scotland, Wales and Northern Ireland. The Stationery Office; 2012.
(47) The Private Healthcare Information Network. *Annual Report 2016-17.* URL: https://apicms.phin.org.uk/Content/Resource/158-PHIN_AR_2016-17.pdf
(48) Herbert A, Wijlaars L, Zylbersztejn A, Cromwell D, Hardelid P. Data Resource Profile: Hospital Episode Statistics Admitted Patient Care (HES APC). *International Journal of Epidemiology*. 2017;46(4):1093-i.
(49) Boyd A, Cornish R, Johnson L, Simmonds S, Syddall H, Westbury L, Cooper C, Macleod J. *Understanding Hospital Episode Statistics (HES)*. London, UK: CLOSER. 2017.
(50) Herbert A, Wijlaars L, Zylbersztejn A, Cromwell D, Hardelid P. Data Resource Profile: Hospital Episode Statistics Admitted Patient Care (HES APC). *International Journal of Epidemiology*. 2017;46(4):1093-i.
(51) Smith T, Noble M, Noble S, Wright G, McLennan D, Plunkett E. The English indices of deprivation 2015. London: Department for Communities and Local Government. 2015 Sep.
(52) Zegers-Hochschild F, Adamson GD, Dyer S, Racowsky C, de Mouzon J, Sokol R, et al. The International Glossary on Infertility and Fertility Care, 2017. *Fertility and Sterility*. 2017;108(3):393-406.
(53) Williams C, Jones M, Swerdlow A, Botting B, Davies M, Jacobs I, et al. Risks of ovarian, breast, and corpus uteri cancer in women treated with assisted reproductive technology in Great Britain, 1991-2010: data linkage study including 2.2 million person years of observation. *British Medical Journal*. 2018;362:k2644.
(54) Yokoyama Y, Shimizu T, Hayakawa K. Incidence of handicaps in multiple births and associated factors. *Acta geneticae medicae et gemellologiae: twin research*. 1995;44(2):81-91.
(55) Doyle P. The outcome of multiple pregnancy. *Human Reproduction*. 1996;11(suppl_4):110-20.

For peer review only

(56) Elm Ev, Altman DG, Egger M, Pocock SJ, Gøtzsche PC, Vandenbroucke JP. Strengthening the reporting of observational studies in epidemiology (STROBE) statement: guidelines for reporting observational studies. *British Medical Journal*. 2007;335(7624):806-8.
(57) Castillo CM, Horne G, Fitzgerald CT, Johnstone ED, Brison DR, Roberts SA. The impact of IVF on birthweight from 1991 to 2015: a cross-sectional study. *Human Reproduction*. 2019;34(5):920-31.
(58) Pelkonen S, Koivunen R, Gissler M, Nuojua-Huttunen S, Suikkari A-M, Hydén-Granskog C, et al. Perinatal outcome of children born after frozen and fresh embryo transfer: the Finnish cohort study 1995–2006. *Human Reproduction*. 2010;25(4):914-23.
(59) Roy TK, Bradley CK, Bowman MC, McArthur SJ. Single-embryo transfer of vitrified-warmed blastocysts yields equivalent live-birth rates and improved neonatal outcomes compared with fresh transfers. *Fertility and Sterility*. 2014;101(5):1294-301. e2.
(60) Litzky JF, Boulet SL, Esfandiari N, Zhang Y, Kissin DM, Theiler RN, et al. Effect of frozen/thawed embryo transfer on birthweight, macrosomia, and low birthweight rates in US singleton infants. *American Journal of Obstetrics and Gynecology*. 2018;218(4):433.e1-.e10.
(61) Berntsen S, Pinborg A. Large for gestational age and macrosomia in singletons born after frozen/thawed embryo transfer (FET) in assisted reproductive technology (ART). *Birth Defects Res*earch. 2018;110(8):630-43.
(62) Terho AM, Pelkonen S, Opdahl S, Romundstad LB, Bergh C, Wennerholm UB, et al. High birth weight and large-for-gestational-age in singletons born after frozen compared to fresh embryo transfer, by gestational week: a Nordic register study from the CoNARTaS group. *Human Reproduction*. 2021.
(63) Elias FTS, Weber-Adrian D, Pudwell J, Carter J, Walker M, Gaudet L, et al. Neonatal outcomes in singleton pregnancies conceived by fresh or frozen embryo transfer compared to spontaneous conceptions: a systematic review and meta-analysis. *Archives of Gynecology and Obstetrics*. 2020;302(1):31-45.
(64) Maheshwari A, Pandey S, Amalraj Raja E, Shetty A, Hamilton M, Bhattacharya S. Is frozen embryo transfer better for mothers and babies? Can cumulative meta-analysis provide a definitive answer? *Human Reproduction Update*. 2018;24(1):35-58.
(65) Sha T, Yin X, Cheng W, Massey IY. Pregnancy-related complications and perinatal outcomes resulting from transfer of cryopreserved versus fresh embryos in vitro fertilization: a meta-analysis. *Fertility and Sterility*. 2018;109(2):330-42.e9.
(66) Castillo CM, Harper J, Roberts SA, O'Neill HC, Johnstone ED, Brison DR. The impact of selected embryo culture conditions on ART treatment cycle outcomes: a UK national study. *Human Reproduction Open*. 2020;2020(1):hoz031.
(67) Berntsen S, Söderström-Anttila V, Wennerholm UB, Laivuori H, Loft A, Oldereid NB, et al. The health of children conceived by ART: 'the chicken or the egg?'. *Human Reproduction Update*. 2019;25(2):137-58.
(68) Luke B, Brown MB, Wantman E, Stern JE, Toner JP, Coddington CC, 3rd. Increased risk of large-for-gestational age birthweight in singleton siblings conceived with in vitro fertilization in frozen versus fresh cycles. *Journal of Assisted Reproduction and Genetics*. 2017;34(2):191-200.
(69) Henningsen AK, Pinborg A. Birth and perinatal outcomes and complications for babies conceived following ART. *Seminars in Fetal & Neonatal Medicine*. 2014;19(4):234-8.
(70) Pinborg A, editor To transfer fresh or thawed embryos? *Seminars in Reproductive Medicine*; 2012: Thieme Medical Publishers.
(71) Kimber SJ, Sneddon SF, Bloor DJ, El-Bareg AM, Hawkhead JA, Metcalfe AD, et al. Expression of genes involved in early cell fate decisions in human embryos and their regulation by growth factors. *Reproduction*. 2008;135(5):635-47.
(72) Goldstein H. Factors related to birth weight and perinatal mortality. *British Medical Bulletin*. 1981;37(3):259-64.
(73) Harron K, Dibben C, Boyd J, Hjern A, Azimaee M, Barreto ML, et al. Challenges in administrative data linkage for research. *Big Data & Society*. 2017;4(2):2053951717745678.

For peer review only

(74) Human Fertilisation and Embryology Authority. *How we manage your information*. https://www.hfea.gov.uk/choose-a-clinic/how-we-manage-your-information/#:~:text=Key%20facts%20Researchers%20need%20your,not%20publish%20your%20identifying%20information.
(75) Spencer SA, Davies MP. Hospital episode statistics: improving the quality and value of hospital data: a national internet e-survey of hospital consultants. *BMJ Open*. 2012;2(6).
(76) Shenfield F, de Mouzon J, Pennings G, Ferraretti AP, Andersen AN, de Wert G, et al. Cross border reproductive care in six European countries. *Human Reproduction*. 2010;25(6):1361-8.

For peer review only

FIGURE LEGENDS

Figure 1: Flowchart showing linkage 1 (HFEA-ONS)

Figure 2: Flowchart showing linkage 2 (HFEA-ONS-HES)

For peer review only

Figure 1: Flowchart showing linkage 1 (HFEA-ONS)

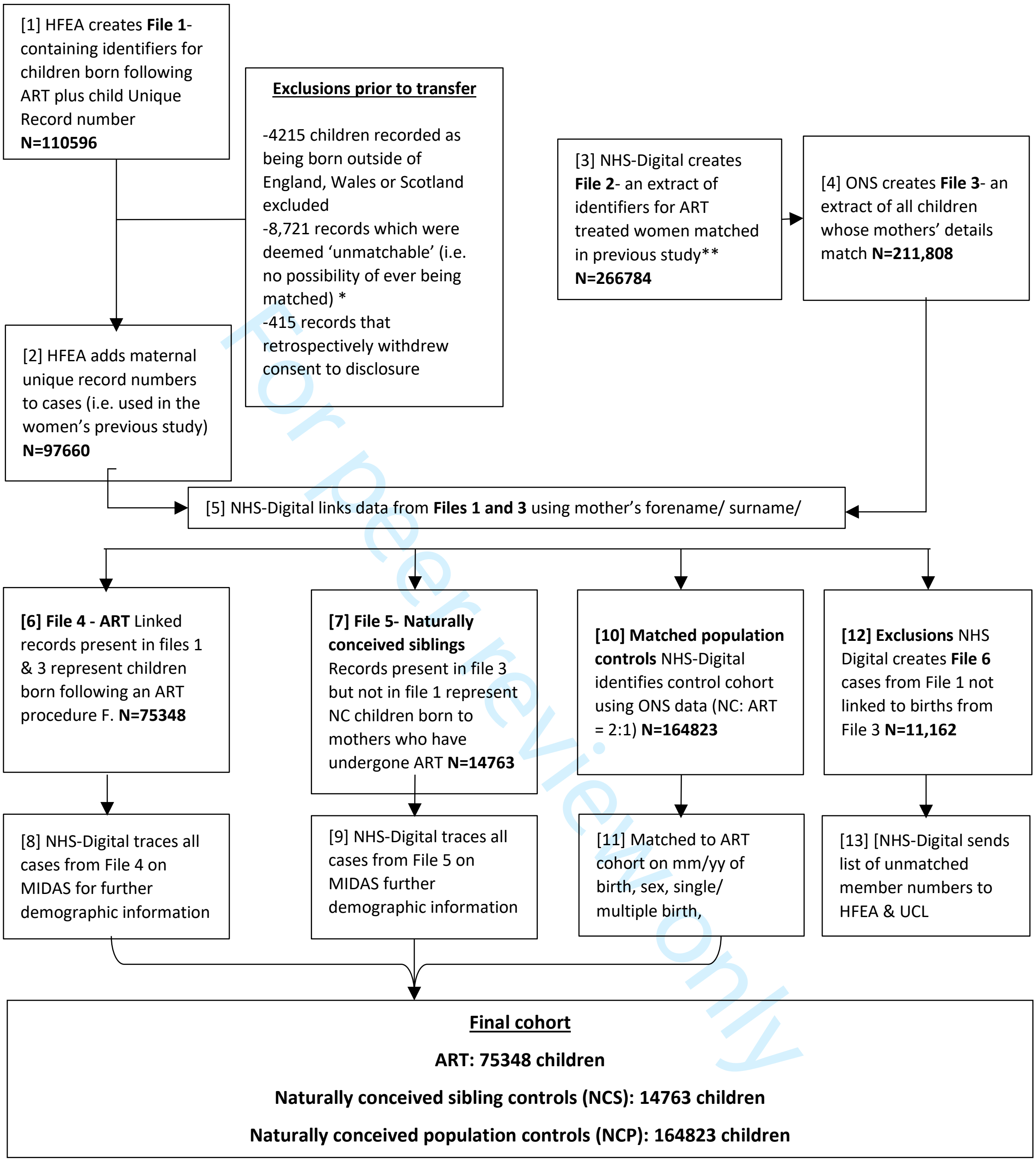

*as they were a) births outside of England/ Wales; b) births before 1993 (when ONS systems were automated and thus the date from which linkage is possible to ONS records); and c) to mothers which were not included in file 2 (as it was not possible to identify them on NHS-Digital systems previously- 'women's study').

** Please see Supplementary figure S2 for cohort flow

For peer review only

Figure 2: Flowchart showing linkage 2 (HFEA-ONS-HES)

[14] NHS Digital produces current status report on all ART, NCS and NCP controls (as produced in linkage 1) and provides de-identified output plus non identifiable deprivation score to UCL.

↓

[15] NHS Digital links cohort data to HES data and provides de-identified output to UCL

↓

[16] UCL interacts with HFEA to obtain fertility treatment clinical data and parental data per matched group member.

UCL match up NHS Digital and clinical HES information to HFEA information using unique record number.

↓

[17] UCL excludes records of children born before 1st April 1997 to coincide with start of HES monitoring.

UCL excludes triplets and higher order births from all groups (along with associated ART, siblings and matched controls)

↓

[18] NHS Digital produces file 4 for each group (identifiable details as below).

- For the ART group, file 4 will be securely transported to the HFEA and stored there securely with highly restricted access.
- For the NCS and NCP groups, file 4 will be securely stored at NHS Digital again with highly restricted access.

↓

Final sub-cohort

HES-ART: 63877

Naturally conceived siblings controls (HES-NCS): 11343

Naturally conceived population controls (HES-NCP): 127544

HES: Hospital Episode Statistics database; UCL: University College London; HFEA: Human Fertilization and Embryology Authority

For peer review only

Supplementary Files

S1: Completeness of variables (for the purpose of linkage and analysis) on the HFEA register

Variable	Useable* entry	Entry 'Null'	No entry	Other**	% useable out of total no. of entries
DOB	110,204	374	0	18	99.6%
Sex of Child<br>*Male*<br>*Female*	110,484<br>*56,265*<br>*54,219*	112	0	0	99.9%
BW	109, 157	534	0	905 records with BW <600g ***	98.7%
Child's Surname	12,332	97,671	0	593	11.2%
Child's Forename	11,402	97,670	0	1524	10.3%
Child's Town of Birth	70,738	39,689	61	108	64.0%
Child's District of Birth	18,833	91,452	298	13	17.0%
Child's Town ***or*** District of Birth	71,650	38,787	51	108	64.8%
Country of Birth	67554	38,861	0	4181 cases born outside of Great Britain	61.1%
Mother's Surname	110,596	0	0	0	100%
Mother's Forename	110,588	0	8	0	100.0%
Mother's Surname at Birth	51,237	58,714	209	436	46.3%
Mother's Forename at Birth	3,323	106,895	377	1	3.0%
Mother's DOB	110,569	1	0	26	100.0%
Father's Surname	110,450	138	8	0	99.9%
Father's Forename	110,384	202	8	2	99.8%
Father's DOB	110,282	257	0	57	99.7%
Mother's Town or District of Birth	62,505	47,045	0	1046	56.5%
Mother's Country of Birth	64,396	46,190	0	10	58.2%
Father's Town or District of Birth	61,143	48,354	0	1099	55.3%
Father's Country of Birth	63,100	47,485	0	11	57.1%
Treatment Cycle Start Date	110,596	0	0	0	100%
Treatment Centre No/ Name	110,594	0	0	2	100.0%

HFEA: Human Fertilization and Embryology Authority; DOB: date of birth; BW: birth weight

* 'Useable' data refers to records which contain data that appears useable in some analyses, including initial quality control analyses.

** 'Other' refers to unusable information for the variable in question, most commonly variations of 'Not known', 'Unknown' etc.

***905 records with documented BWs less than 600g were considered as invalid information for the purpose of deterministic linkage. Whilst a small number of these BWs (<600g) are likely to be correct, many more are likely to be data errors within the HFEA register. All actual recorded BWs were used when manually viewing potential matches.

This table was shared with us by HFEA at the start of the study.

For peer review only

S2- Flow diagram showing derivation of File 2 in Figure 1.

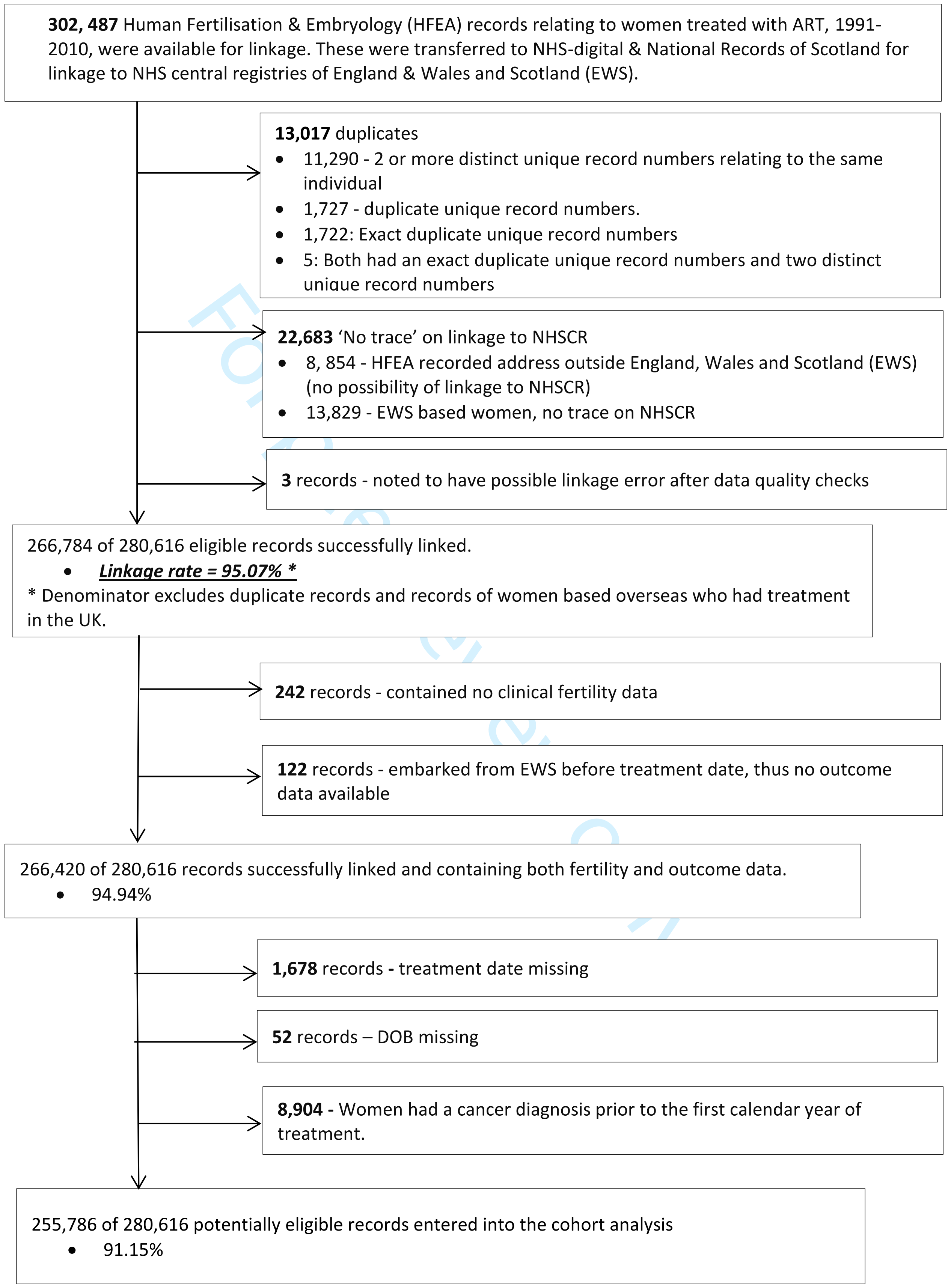

HFEA: Human Fertilization and Embryology Authority; DOB: date of birth; EWS: England, Wales & Scotland; NHSCR: National Health Service Central Register

S3: Order of pregnancy (HFEA-ONS cohort)

No. of pregnancies	Order of pregnancy	ART		Spontaneously conceived siblings	
		Singleton pregnancy	Twin Pregnancy	Singleton pregnancy	Twin Pregnancy
**2 (n=8441)**	1	5135	1182	2095	29
	2	1596	528	6161	156
**3 (n=1336)**	1	918	107	304	7
	2	453	96	777	10
	3	126	26	1155	29
**4 (n=125)**	1	83	9	33	0
	2	42	5	77	1
	3	19	6	99	1
	4	7	4	113	1
**5 (n=12)**	1	8	1	2	1
	2	5	1	6	0
	3	2	0	10	0
	4	0	0	12	0
	5	0	0	12	0
**6 (n=1)**	1	1	0	0	0
	2	0	0	1	0
	3	0	0	1	0
	4	0	0	1	0
	5	0	0	1	0
	6	0	0	1	0
**7 (n=1)**	1	0	1	0	0
	2	0	0	1	0
	3	0	0	1	1
	4	0	0	0	0
	5	0	0	1	0
	6	0	0	1	0
	7	0	0	1	0

HFEA: Human Fertilization and Embryology Authority; ONS: Office for National Statistics.

For peer review only

S4: Multiple regression coefficients for adjusted mean BW- ART vs NCP (HFEA-ONS)

Variables	BW Coefficient	95% Confidence interval
Embryo transfer status		
Fresh	-131.4	(-139.5 - -123.4)
Frozen	35.24	(18.97 - 51.52)
Sex		
Male	119.1	(112.7 - 125.5)
Maternal age group		
25-29	66.68	(55.89 - 77.48)
30-34	111.9	(102.2 - 121.6)
35-39	129.2	(118.7 - 139.7)
40-44	132.3	(118.0 - 146.5)
>=45	72.66	(-13.46 - 158.8)
**Constant**	3,215	(3,207 - 3,223)
**Observations**	129,304	
**R-squared**	0.021	

BW: birth weight; NCP: naturally conceived population controls; HFEA: Human Fertilization and Embryology Authority; ONS: Office for National Statistics.

For peer review only

S5: Multiple regression coefficients for adjusted mean BW- sART vs NCS (HFEA-ONS)

VARIABLES	BW coefficient	95% Confidence interval
Embryo transfer status		
Fresh	-54.69	(-72.64 - -36.73)
Frozen	138.6	(101.3 - 175.9)
Sex		
Male	115.6	(97.78 - 133.4)
Maternal age group		
25-29	-43.23	(-114.2 – 27.74)
30-34	-98.50	(-176.5 - -20.46)
35-39	-117.1	(-206.8 - -27.41)
40-44	-161.8	(-266.1 - -57.52)
>=45	-198.7	(-409.2 – 11.79)
Order of pregnancy		
2	165.6	(142.2 – 188.9)
>=3	237.0	(194.4 – 279.6)
**Constant**	3,337	(3,263 - 3,411)
**Observations**	22,086	
**R-squared**	0.081	

BW: birth weight; sART: ART with siblings; NCS- naturally conceived siblings; HFEA: Human Fertilization and Embryology Authority; ONS: Office for National Statistics.

For peer review only

S6: Multiple regression coefficients for adjusted mean BW- ART vs NCP (HFEA-ONS-HES cohort)

Variables	BW coefficient	95% confidence interval
Embryo transfer status		
Fresh	-152.2	(-162.2 - -142.3)
Frozen	2.855	(-16.61 - 22.32)
Sex		
Male	120.6	(112.7 - 128.6)
Maternal age group		
25-29	57.25	(43.52 - 70.99)
30-34	86.40	(73.80 - 99.00)
35-39	91.20	(77.62 - 104.8)
40-44	98.57	(80.37 - 116.8)
45-49	4.103	(-63.56 - 71.77)
Missing	94.69	(60.19 - 129.2)
IMD at earliest appointment		
2	25.95	(8.336 - 43.57)
3	56.66	(38.69 - 74.64)
4	58.78	(40.66 - 76.91)
5	75.13	(56.93 - 93.34)
6	93.33	(75.06 - 111.6)
7	93.21	(75.17 - 111.2)
8	111.8	(93.76 - 129.8)
9	117.3	(99.48 - 135.1)
10	134.7	(116.5 - 152.8)
Derived ethnicity		
Asian/Asian British	-247.8	(-264.2 - -231.4)
Black/African/Caribbean/Black British	-138.2	(-161.2 - -115.2)
Chinese	-120.1	(-195.7 - -44.43)
Mixed/multiple ethnic groups	-66.13	(-94.92 - -37.33)
Not stated/Not known	5.034	(-5.060 - 15.13)
Other ethnic group	-49.19	(-82.25 - -16.13)
**Constant**	3,177	(3,162 - 3,192)
**Observations**	85,366	
**R-squared**	0.040	

BW: birth weight; NCP- naturally conceived population controls; HFEA: Human Fertilization and Embryology Authority; ONS: Office for National Statistics; HES: Hospital Episode Statistics; IMD: Index of Multiple Deprivation

For peer review only

S7: Multiple regression coefficients for adjusted mean BW- sART vs NCS (HFEA-ONS-HES cohort)

Variables	BW coefficient	95% Confidence interval
Embryo transfer status		
Fresh	-57.01	(-75.79 - -38.23)
Frozen	152.1	(113.5 - 190.7)
Sex		
Male	115.6	(96.95 – 134.2)
Maternal age group		
25-29	-28.73	(-104.3 - -46.84)
30-34	-81.27	(-164.9 – 2.335)
35-39	-96.01	(-192.0 - -0.010)
40-44	-135.4	(-246.9 - -23.89)
>=45	-141.6	(-308.8 - -25.69)
Order of pregnancy		
2	152.2	(127.8 – 176.6)
>=3	229.6	(184.3 – 274.8)
**Constant**	3,332	(3,252 - 3,412)
**Observations**	19180	
**R-squared**	0.09	

BW: birth weight; sART: ART with siblings; NCS- naturally conceived siblings; HFEA: Human Fertilization and Embryology Authority; ONS: Office for National Statistics.

S8: Distribution of maternal age in records born before 1997 and not linked to HES

	ART	CONTROL	SIBLING
Maternal age group			
<=25	91 (0.79%)	5804 (15.57%)	84 (2.45%)
25-29	1360 (11.84%)	5496 (14.74%)	179 (5.22%)
30-34	4781 (41.62%)	7592 (20.37%)	636 (18.55%)
35-39	4318 (37.59%)	4738 (12.71%)	873 (25.46%)
40-44	921 (8.02%)	1160 (3.11%)	357 (10.41%)
>=45	11 (0.10%)	132 (0.35%)	27 (0.79%)
Missing	5 (0.04%)	12357 (33.15%)	1273 (37.12%)

HES: Hospital Episode Statistics

For peer review only

For peer review only

For peer review only

Reporting checklist for cohort study.

Based on the STROBE cohort guidelines.

Instructions to authors

Complete this checklist by entering the page numbers from your manuscript where readers will find each of the items listed below.

Your article may not currently address all the items on the checklist. Please modify your text to include the missing information. If you are certain that an item does not apply, please write "n/a" and provide a short explanation.

Upload your completed checklist as an extra file when you submit to a journal.

In your methods section, say that you used the STROBE cohort reporting guidelines, and cite them as:

von Elm E, Altman DG, Egger M, Pocock SJ, Gotzsche PC, Vandenbroucke JP. The Strengthening the Reporting of Observational Studies in Epidemiology (STROBE) Statement: guidelines for reporting observational studies.

		Reporting Item	Page Number
**Title and abstract**			
Title	#1a	Indicate the study's design with a commonly used term in the title or the abstract	1 & 2

For peer review only

Abstract	#1b	Provide in the abstract an informative and balanced summary of what was done and what was found	2
**Introduction**			
Background / rationale	#2	Explain the scientific background and rationale for the investigation being reported	4
Objectives	#3	State specific objectives, including any prespecified hypotheses	5, 6
**Methods**			
Study design	#4	Present key elements of study design early in the paper	6,7,8
Setting	#5	Describe the setting, locations, and relevant dates, including periods of recruitment, exposure, follow-up, and data collection	7, 8
Eligibility criteria	#6a	Give the eligibility criteria, and the sources and methods of selection of participants. Describe methods of follow-up.	7, 8
Eligibility criteria	#6b	For matched studies, give matching criteria and number of exposed and unexposed	7, 8

For peer review only

Variables	#7	Clearly define all outcomes, exposures, predictors, potential confounders, and effect modifiers. Give diagnostic criteria, if applicable	7, 8
Data sources / measurement	#8	For each variable of interest give sources of data and details of methods of assessment (measurement). Describe comparability of assessment methods if there is more than one group. Give information separately for for exposed and unexposed groups if applicable.	6,7
Bias	#9	Describe any efforts to address potential sources of bias	10<br>Creation of two control cohorts to minimize bias
Study size	#10	Explain how the study size was arrived at	N/A<br>Population-based study.
Quantitative variables	#11	Explain how quantitative variables were handled in the analyses. If applicable, describe which groupings were chosen, and why	11

For peer review only

Statistical methods	#12a	Describe all statistical methods, including those used to control for confounding	10, 11
Statistical methods	#12b	Describe any methods used to examine subgroups and interactions	11
Statistical methods	#12c	Explain how missing data were addressed	8-11
Statistical methods	#12d	If applicable, explain how loss to follow-up was addressed	N/A
Statistical methods	#12e	Describe any sensitivity analyses	11
**Results**			
Participants	#13a	Report numbers of individuals at each stage of study—eg numbers potentially eligible, examined for eligibility, confirmed eligible, included in the study, completing follow-up, and analysed. Give information separately for for exposed and unexposed groups if applicable.	11, 12, Figure 1 & 2
Participants	#13b	Give reasons for non-participation at each stage	N/A
Participants	#13c	Consider use of a flow diagram	Figure 1 & 2

For peer review only

Descriptive data	#14a	Give characteristics of study participants (eg demographic, clinical, social) and information on exposures and potential confounders. Give information separately for exposed and unexposed groups if applicable.	13, 14, 16 ,17
Descriptive data	#14b	Indicate number of participants with missing data for each variable of interest	13, 14, 16 ,17
Descriptive data	#14c	Summarise follow-up time (eg, average and total amount)	13, 14, 16 ,17
Outcome data	#15	Report numbers of outcome events or summary measures over time. Give information separately for exposed and unexposed groups if applicable.	13, 14, 16 ,17
Main results	#16a	Give unadjusted estimates and, if applicable, confounder-adjusted estimates and their precision (eg, 95% confidence interval). Make clear which confounders were adjusted for and why they were included	17, 18
Main results	#16b	Report category boundaries when continuous variables were categorized	13, 14, 16 ,17

For peer review only

Main results	#16c	If relevant, consider translating estimates of relative risk into absolute risk for a meaningful time period	N/A
Other analyses	#17	Report other analyses done—eg analyses of subgroups and interactions, and sensitivity analyses	N/A
**Discussion**			
Key results	#18	Summarise key results with reference to study objectives	18
Limitations	#19	Discuss limitations of the study, taking into account sources of potential bias or imprecision. Discuss both direction and magnitude of any potential bias.	20
Interpretation	#20	Give a cautious overall interpretation considering objectives, limitations, multiplicity of analyses, results from similar studies, and other relevant evidence.	18, 19
Generalisability	#21	Discuss the generalisability (external validity) of the study results	20
**Other Information**			

For peer review only

Funding	#22	Give the source of funding and the role of the funders for the present study and, if applicable, for the original study on which the present article is based	22

None The STROBE checklist is distributed under the terms of the Creative Commons Attribution License CC-BY. This checklist can be completed online using https://www.goodreports.org/, a tool made by the EQUATOR Network in collaboration with Penelope.ai

For peer review only
